# Age-Specific Analysis of the Effects of Intermittent Fasting on Body Composition and Cardiometabolic Markers in Healthy Adults and Individuals with Overweight or Obesity: A Systematic Review and Meta-Analysis of Randomized Controlled Trials

**DOI:** 10.3390/nu18111799

**Published:** 2026-06-03

**Authors:** Kaijun Xing, Ruihan Liu, Shenglin Peng, Xuanxuan Zi, Linxi Lian, Bowen Yang, Yangyang Cen, Yichao Li, Yi Zhao, Yannan Zhang

**Affiliations:** 1 School of Public Health, Ningxia Medical University, Yinchuan 750004, China; xkj0420@163.com (K.X.); 15774681897@163.com (R.L.); 15367227391@163.com (S.P.); 18238839267@163.com (X.Z.); 13598526169@163.com (L.L.); ybw230240310135@nxmu.edu.cn (B.Y.); 230240310137@nxmu.edu.cn (Y.C.); lyc02031@163.com (Y.L.); 20040017@nxmu.edu.cn (Y.Z.); 2 Ningxia Key Laboratory of Environmental Factors and Chronic Disease Control, Yinchuan 750004, China

**Keywords:** age-stratified effect, dietary intervention, weight management, metabolic regulation, intermittent fasting, meta-analysis

## Abstract

**Background**: Intermittent fasting (IF) is a popular dietary strategy for improving weight and cardiometabolic health. However, its effectiveness and potential risks across different adult age trajectories remain unclear. This systematic review and meta-analysis evaluated the age-specific effects of IF on body composition and cardiometabolic markers. **Methods**: Following PRISMA 2020 guidelines, PubMed, Scopus, and Web of Science were searched for randomized controlled trials (RCTs) up to September 2025. Participants were stratified into three cohorts: <30 years, 30–44 years, and ≥45 years. Random-effects meta-analyses and leave-one-out sensitivity analyses were conducted on body composition, lipid profiles, glycemic markers, and blood pressure. Additionally, a conservative methodological sensitivity analysis (imputed correlation r = 0.5) and subgroup analyses by fasting modality (TRF vs. intermittent energy restriction) were performed. Risk of bias was assessed using the RoB 2 tool. **Results**: Analysis of 28 RCTs (N = 1833) demonstrated that IF significantly reduced body weight and BMI across all age groups. Notably, subgroup analyses revealed comparable physiological responses between TRF and intermittent energy restriction modalities. Cardiometabolic adaptations were highly age-dependent. Young adults exhibited significant reductions in fasting insulin and HOMA-IR, alongside a robust reduction in fat mass. However, a significant loss of fat-free mass (FFM) was observed in both young and older cohorts. While middle-aged and older adults experienced the most pronounced improvements in triglycerides, systolic blood pressure, and insulin sensitivity, our conservative sensitivity analysis unmasked a significant elevation in low-density lipoprotein cholesterol (LDL-C) in this group, mirroring the robust LDL-C increase observed in young adults. Early middle-aged adults exhibited highly variable responses with no significant overall improvements in cardiometabolic parameters. **Conclusions**: IF is an effective weight-management tool, but elicits distinct, age-specific metabolic trajectories. While middle-aged and older adults derive pronounced cardiometabolic benefits, they face critical risks of lean mass depletion, necessitating a combined “IF+” strategy (adequate protein and resistance training). Crucially, the age-specific risk of LDL-C elevation dictates a mandate for vigilant lipid monitoring. Given that the certainty of evidence was rated as low to very low per GRADE criteria, these age-specific patterns should be interpreted as hypothesis-generating, warranting validation in future large-scale trials.

## 1. Introduction

Intermittent fasting (IF), a prevalent dietary intervention model, has garnered extensive attention due to its potential in weight management and metabolic health improvement. As an alternative to traditional calorie restriction, IF—along with meal timing adjustments aimed at promoting weight loss and enhancing cardiometabolic health—may offer better adherence [1,2,3]. IF involves periods of fasting or strict energy restriction, interspersed with periods of ad libitum eating. Its modalities include Alternate-Day Fasting (ADF), modified ADF, the 5:2 diet, Time-Restricted Feeding (TRF), and Periodic Fasting (PF) [1,4,5,6]. Among these, TRF, ADF, and the 5:2 diet are the most well-known. TRF is a dietary approach that permits energy intake only within a predefined daily time window [7]. In ADF, individuals alternate between energy-restricted days and ad libitum eating days [8,9]. The 5:2 diet entails two consecutive or non-consecutive energy-restricted days per week, with habitual energy intake maintained on the remaining five days [8].

A growing body of evidence suggests that IF may be an effective approach for weight loss and fat reduction, and it also contributes to improvements in cardiometabolic health markers. Multiple meta-analyses have demonstrated clinical improvements in lipid profiles, blood pressure, and insulin resistance associated with IF [10,11,12,13,14], a critical methodological limitation persists: the vast majority of existing clinical trials and subsequent meta-analyses have treated adult populations as a homogenous group, thereby masking critical age-specific therapeutic windows or potential metabolic risks. Biologically, chronological aging is inextricably linked with progressive shifts in neuroendocrine regulation, metabolic rate, and body composition. Across the adult lifespan, predictable declines in growth hormone secretion, alterations in sex steroids (such as estrogen and testosterone), and a steady reduction in basal metabolic rate collectively modify the threshold for adiposity and insulin sensitivity. These age-dependent biological baselines strongly imply that dietary interventions, such as IF, may not elicit uniform physiological adaptations, but rather trigger highly divergent metabolic responses depending on the individual’s position along the aging trajectory. Despite this biological rationale, individual randomized controlled trials (RCTs) often suffer from small sample sizes and are generally underpowered to conduct robust age-stratified analyses. Therefore, there is an urgent need to comprehensively evaluate existing studies through systematic evidence synthesis to clarify whether the health effects of IF vary with age.

This systematic review and meta-analysis aims to elucidate the age-specific efficacy of intermittent fasting regimens on body composition and cardiometabolic health. Crucially, reflecting the demographic most frequently targeted by IF interventions, this study focuses specifically on healthy individuals and those with overweight or obesity. The investigation is structured around the PICO framework, with the central research question defined as follows: In generally healthy adults and individuals with overweight or obesity, stratified into young adults (<30 years), early middle-aged adults (30–44 years), and middle-aged and older adults (≥45 years) (P), how do structured intermittent fasting regimens—including TRF, ADF, and the 5:2 diet (I)—compare to regular, unrestricted dietary patterns (C) in modulating a comprehensive set of primary outcomes? These outcomes encompass body composition parameters—specifically body weight, fat mass (FM), fat-free mass (FFM), and body mass index (BMI)—and key cardiometabolic markers, including the lipid profile (total cholesterol, triglycerides, high-density lipoprotein cholesterol, low-density lipoprotein cholesterol), glycemic control indices (fasting glucose, fasting insulin, HOMA-IR), and systolic and diastolic blood pressure (SBP/DBP) (O).

Ultimately, this analysis seeks to synthesize age-stratified evidence to inform more targeted, practical, and personalized dietary guidance for implementing IF across the adult lifespan.

Ultimately, this analysis seeks to synthesize age-stratified evidence to inform more targeted and practical dietary guidance for implementing IF across the adult lifespan.

## 2. Materials and Methods

### 2.1. Research Design and Registration

This systematic review and meta-analysis were conducted in strict accordance with the Preferred Reporting Items for Systematic Reviews and Meta-Analyses (PRISMA) guidelines (Supplemental Files S1a and S1b) and the Cochrane Handbook for Systematic Reviews of Interventions to ensure methodological rigor and full transparency of the research process [15,16]. The review protocol was prospectively registered on the International Prospective Register of Systematic Reviews (PROSPERO) under registration number CRD420251028745, thereby minimizing the risks of design bias and selective outcome reporting. During the research process, we did not make any modifications to the research plan.

### 2.2. Search Strategy

A comprehensive search was conducted across multiple electronic databases, including PubMed, Scopus, and Web of Science, from database inception to September 2025 to ensure extensive literature coverage. Keywords were developed around three core themes: “intermittent fasting”, “body composition”, and “cardiometabolic health”. Specific terms related to intermittent fasting included “time-restricted feeding,” “intermittent fasting,” and “alternate-day fasting,” among others. The search was restricted to English-language publications and human studies. The detailed search strategy for each database is provided in Supplementary File S2. All identified records were imported into EndNote 21 for automatic duplicate removal. The screening process consisted of two phases: initially, articles were screened based on titles and abstracts; subsequently, the full texts of potentially eligible studies were thoroughly assessed to determine final inclusion. In addition to the electronic database searches, a manual screening of the reference lists of included studies was performed to identify any potentially eligible articles that might have been overlooked. All screening steps were performed independently by two reviewers to ensure accuracy and consistency.

### 2.3. Inclusion Criteria

P (Population): Adults aged ≥18 years, including both generally healthy individuals and those with overweight or obesity (typically defined as BMI ≥ 25 kg/m^2^). Crucially, to isolate the physiological effects of the dietary intervention from disease-specific confounders or medication interactions, participants were required to be free of diagnosed clinical chronic conditions, such as diabetes mellitus, cardiovascular disease, or overt metabolic syndrome.

I (Intervention): Any form of intermittent fasting, including alternate-day fasting (ADF), the 5:2 diet, time-restricted feeding (TRF), and the 16:8 protocol.

C (Comparison): Regular diet as the control.

O (Outcomes): At least one body composition indicator (e.g., body weight, fat mass) or cardiometabolic parameter (e.g., fasting blood-glucose, blood lipids, blood pressure).

S (Study design): Parallel-group randomized controlled trials (RCTs), published in full-text, peer-reviewed, and in English.

### 2.4. Exclusion Criteria

Non-original studies, animal studies, non-RCTs, and studies involving participants with diagnosed clinical diseases (e.g., diabetes mellitus) were excluded.

### 2.5. Data Extraction and Synthesis

Data extraction was performed independently by two reviewers, with any discrepancies resolved through team consensus. The extracted data included basic study information (first author, publication year, study design), participant characteristics (sample size, sex, age, BMI, health status), intervention details (IF/CON protocol, duration), and outcome data (pre- and post-intervention means, standard deviations [SDs], and sample sizes). When studies reported only pre- and post-intervention means and SDs, the mean changes and their SDs were calculated using formulas recommended in the Cochrane Handbook, assuming a standard correlation coefficient of 0.8. To ensure the robustness of our findings and to address the potential false narrowing of confidence intervals caused by overly optimistic correlation assumptions, we conducted a methodological sensitivity analysis by recalculating the effect sizes for primary outcomes using a highly conservative correlation coefficient of r = 0.5. If data were presented as standard errors, medians (with interquartile ranges), or confidence intervals, they were converted to means and SDs using the methods described by Wan et al. (2014) [17]. To prevent unit-of-analysis errors, for multi-arm trials or studies providing multiple relevant subgroups that shared a single control group, we halved the sample size of the shared control group in our meta-analysis while leaving the means and SDs unchanged. In cases of multiple publications from the same cohort, only unique outcome data were extracted. If critical data were missing, corresponding authors were contacted via email. To ensure analytical consistency, lipid profiles and glycemic parameters were converted to uniform units using standard conversion formulas [11,18].

### 2.6. Risk of Bias Assessment

The risk of bias (RoB) for each included study was assessed using the Cochrane Risk of Bias 2 (RoB 2) tool for parallel-group trials [19]. The RoB 2 tool evaluates five domains: (1) bias arising from the randomization process; (2) bias due to deviations from intended interventions; (3) bias due to missing outcome data; (4) bias in measurement of the outcome; and (5) bias in the selection of the reported result. Each domain was rated as having a ‘low risk of bias’, ‘some concerns’, or ‘high risk of bias’. An overall RoB judgment for each study was then determined based on the RoB 2 algorithmic guidance. The quality assessment was conducted independently by two reviewers. The final risk of bias assessment results were visualized as traffic light and summary plots using R 4.5.2 [20].

### 2.7. Statistical Methods for Meta-Analysis

For continuous outcome parameters, the mean change from baseline to post-intervention in the IF group was compared against that of the control group, and pooled estimates were computed as mean differences (MDs) with 95% confidence intervals (CIs). Furthermore, to rigorously evaluate the clinical feasibility and participant adherence to the fasting interventions, we analyzed overall and age-stratified dropout rates as a proxy for compliance. For this dichotomous outcome, pooled estimates were calculated as risk ratios (RRs) with 95% CIs, where an RR greater than 1.0 would indicate a higher risk of dropout in the IF group. All pooled estimates were generated using a random-effects model due to anticipated clinical and methodological heterogeneity across studies, and the detailed study-level results for each individual outcome were visualized using comprehensive forest plots. Furthermore, to provide an intuitive and overarching synthesis of the complex, age-stratified effect sizes and their statistical significance, an aggregated color-coded summary heatmap was generated.

To systematically evaluate potential sources of clinical heterogeneity, predefined subgroup analyses were conducted based on participant age and intermittent fasting modalities. To systematically evaluate potential sources of clinical heterogeneity, predefined subgroup analyses were conducted based on participant age and intermittent fasting modalities. First, to capture distinct metabolic and chronological lifecycles, participants were stratified into three precise cohorts: young adults (<30 years), early middle-aged adults (30–44 years), and middle-aged and older adults (≥45 years). These cutoffs are robustly justified by established physiological milestones and molecular evidence of metabolic aging: (1) distinct biological transition points, as systemic proteomic aging clocks and growth hormone axes demonstrate a critical inflection and accelerated shift in the third and fourth decades, whereas clinical phenotypes of progressive vascular stiffening and systemic insulin resistance typically accelerate after the age of 45 [21,22]; (2) epidemiological trajectories defining cardiometabolic risk transitions across lifespans [23]; and (3) statistical feasibility, ensuring an optimal, balanced distribution of randomized controlled trials (RCTs) across each distinct stratum to maintain sufficient statistical power. To determine whether different fasting strategies elicit distinct physiological responses, interventions were categorized by IF protocol type into time-restricted feeding (TRF) and intermittent energy restriction (IER, encompassing alternate-day fasting and the 5:2 diet). Subgroup differences were assessed using the test for subgroup interactions.

Between-study heterogeneity was assessed using the I^2^ statistic and interpreted as low (I^2^ < 25%), moderate (25% ≤ I^2^ < 75%), or high (I^2^ ≥ 75%) heterogeneity. To explore potential sources of heterogeneity, random-effects meta-regression analyses were conducted (e.g., investigating the association between intervention duration and LDL-C changes), and the results were visually represented using bubble plots, where the size of each bubble corresponds to the statistical weight of the respective study. Publication bias was explored via contour-enhanced funnel plots and Egger’s test. To systematically evaluate the robustness of our pooled estimates, we conducted a comprehensive leave-one-out sensitivity analysis across all outcomes and age subgroups. The fluctuation ranges of the MDs and the stability of statistical significance were meticulously recorded to ensure that findings were not disproportionately driven by extreme outliers. All standard analyses were performed using Review Manager (RevMan) version 5.4 (The Cochrane Collaboration) and the meta package in R 4.5.2.

### 2.8. Grading the Quality of Evidence

The “Grades of Recommendations, Assessment, Development, and Evaluation” (GRADE) tool was utilized to determine the certainty of the evidence. Each outcome was rated as having high-, moderate-, low-, or very low-certainty evidence based on study design, risk of bias, inconsistency, indirectness, imprecision, and publication bias.

## 3. Results

### 3.1. Study Selection

**Figure 1:**
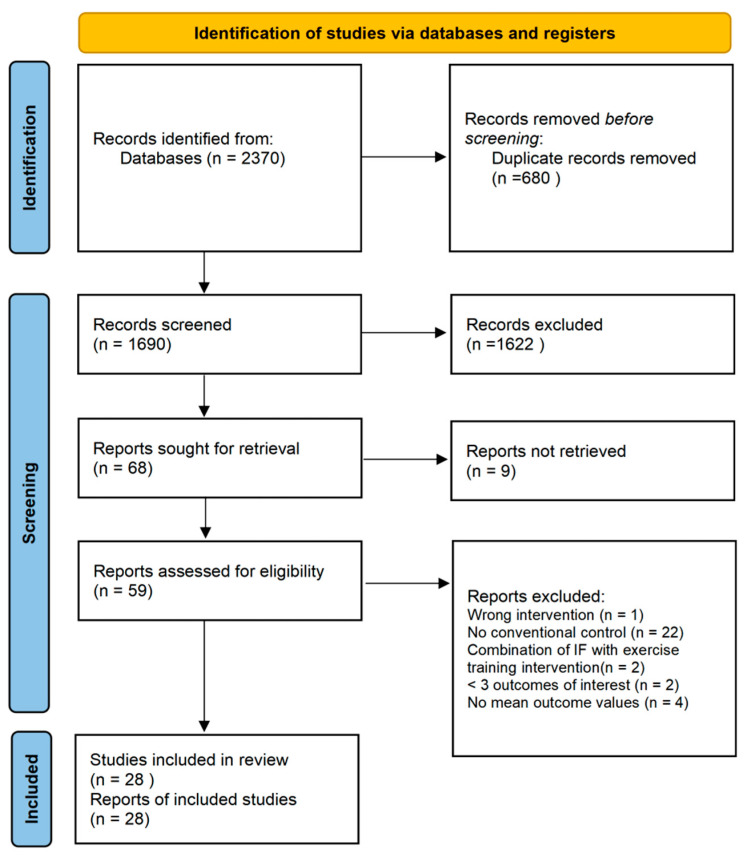
PRISMA flow diagram for the selection process and inclusion of the human randomized controlled trials. IF, intermittent fasting; PRISMA, preferred reporting items for systematic reviews and meta-analyses.

Figure 1 illustrates the study selection process. Initially, 2370 records were identified through database searches. After removing 680 duplicates, 1690 unique records remained. Screening of titles and abstracts led to the exclusion of 1622 articles. Nine reports could not be retrieved. The full texts of the remaining 59 articles were assessed for eligibility. The excluded studies along with the reasons for exclusion are available in Supplemental Table S1 [24,25,26,27,28,29,30,31,32,33,34,35,36,37,38,39,40,41,42,43,44,45,46,47,48,49,50,51,52,53,54]. Ultimately, 28 studies met the inclusion criteria.

### 3.2. Study Characteristics

**Figure 2:**
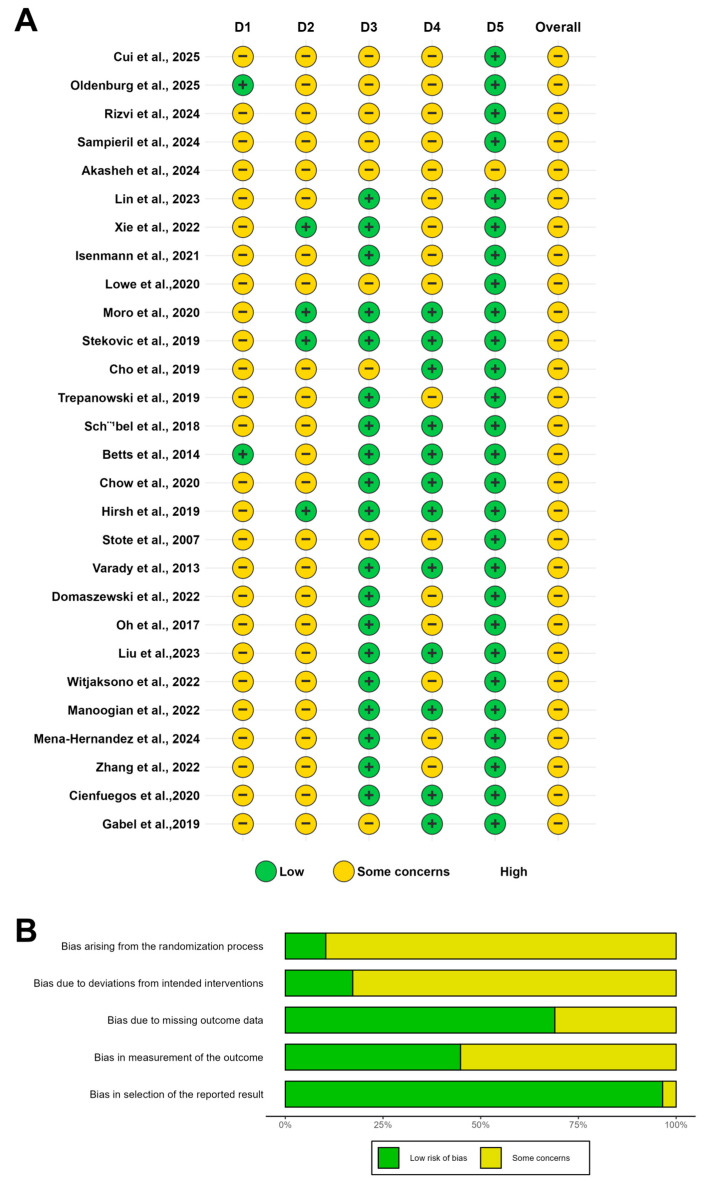
Risk of bias assessment for the included randomized controlled trials. The assessment was conducted using the Cochrane Risk of Bias 2 (RoB 2) tool. (**A**) Traffic light plot detailing the domain-level and overall risk of bias judgments for each individual study [55,56,57,58,59,60,61,62,63,64,65,66,67,68,69,70,71,72,73,74,75,76,77,78,79,80,81,82]. (**B**) Summary plot illustrating the aggregated proportion of studies categorized as having a low risk, some concerns, or high risk of bias across each domain. Green circles/bars represent a low risk of bias, yellow indicate some concerns, and red represent a high risk of bias. Assessed domains include: D1, bias arising from the randomization process; D2, bias due to deviations from intended interventions; D3, bias due to missing outcome data; D4, bias in measurement of the outcome; D5, bias in selection of the reported result.

The combined studies included 1833 participants, comprising healthy adults and those with overweight or obesity, with mean ages and body mass index (BMI) ranging from 21 to 70 years and 20.3 to 38.0 kg/m^2^, respectively (Table 1). Crucially, all participants were free of diagnosed clinical conditions such as prediabetes, type 2 diabetes, metabolic syndrome, non-alcoholic fatty liver disease, or gestational diabetes. Except for one study that enrolled only female participants [55] and another including only males [56], all other studies included both sexes. The intervention durations varied from 4 weeks to 12 months. The majority of studies employed time-restricted feeding (TRF) [55,56,57,58,59,60,61,62,63,64,65,66,67,68,69,70,71,72,73], while alternate-day fasting (ADF) [70,74,75,76,77,78,79] and the 5:2 diet [80,81] were used in the remaining trials. A summary of the risk of bias assessment for the included studies is presented in Figure 2 and Supplementary Table S4.

### 3.3. Age-Specific Analysis of the Effects of Intermittent Fasting on Body Composition

The meta-analysis results, including pooled effect sizes, confidence intervals, and heterogeneity across the three age cohorts, are detailed in Table 2. These age-stratified effects on all 13 parameters are visually synthesized in a heatmap (Figure 3), with individual forest plots for each outcome provided in the Supplementary Materials (Supplementary Figures S22–S34).

A total of nine [56,57,62,63,69,70,71,74,80], seven [55,58,59,67,72,77,79], and five [61,64,66,68,73] trials were analyzed for body weight in the age groups of <30, 30–44, and ≥45 years, respectively. Specifically, weight loss was robust in young adults (<30 years: nine studies [11 comparisons], N = 373; MD = −1.80 kg, 95% CI [−2.57, −1.03], *p* < 0.001), early middle-aged adults (30–44 years: seven studies [nine comparisons], N = 453; MD = −1.47 kg, 95% CI [−2.44, −0.49], *p* = 0.003), and middle-aged and older adults (≥45 years: five studies [six comparisons], N = 253; MD = −2.16 kg, 95% CI [−3.67, −0.65], *p* = 0.005). Parallel significant reductions were also observed in BMI across all three cohorts (all *p* ≤ 0.009). Leave-one-out sensitivity analyses confirmed that these weight and BMI reductions were exceptionally robust, with statistical significance unaffected by the successive omission of any single trial (Supplementary Table S2, Supplementary Figures S22 and S23).

However, adaptations in fat mass (FM) and fat-free mass (FFM) exhibited distinct, age-dependent patterns. Significant reductions in FM were exclusively observed in the young (MD = −1.09 kg, *p* < 0.001) and older (MD = −1.49 kg, *p* < 0.001) cohorts, whereas the reduction in early middle-aged adults was marginally non-significant (MD = −1.22 kg, 95% CI [−2.55, 0.10], *p* = 0.070). Crucially, IF induced significant FFM (lean muscle) loss in both young adults (MD = −0.98 kg, *p* = 0.020) and middle-aged and older adults (MD = −0.98 kg, *p* < 0.001), but not in early middle-aged participants (MD = −0.60 kg, *p* = 0.154, Supplementary Figures S24 and S25). Despite moderate-to-high heterogeneity in some subgroups, sensitivity analyses demonstrated that all these body composition outcomes remained highly robust.

### 3.4. Age-Specific Effects of Intermittent Fasting on Blood Lipids

The impact of IF on lipid profiles varied markedly by age. Total cholesterol (TC) and high-density lipoprotein cholesterol (HDL-C) levels were not significantly altered by IF in any of the age groups (all *p* > 0.05, Supplementary Figures S29 and S31). Notably, the leave-one-out analysis identified the pooled TC estimate for middle-aged and older adults as statistically fragile, indicating high sensitivity to individual study variations. In contrast, triglycerides (TG) were significantly reduced only in the older adult cohort (MD = −7.83 mg/dL, 95% CI [−12.47, −3.20], *p* < 0.001, Supplementary Figure S30), an effect that was confirmed as highly robust.

The most striking age-related divergence was observed in low-density lipoprotein cholesterol (LDL-C). In the young cohort (<30 years), IF provoked a substantial and highly significant increase in LDL-C (MD = 6.75 mg/dL, 95% CI [3.15, 10.34], *p* < 0.001, Supplementary Figure S32). Conversely, the standard analysis showed no significant effect on LDL-C in early middle-aged (MD = 1.44 mg/dL, *p* = 0.424) and middle-aged and older adults (MD = 0.19 mg/dL, *p* = 0.934). To further explore this age-dependent phenomenon, a random-effects meta-regression was conducted. The analysis yielded a regression coefficient of −0.65 (95% CI [−1.61, 0.31], *p* = 0.180). Although statistical significance was not reached—likely due to the limited number of studies available for this specific regression (k = 6)—the accompanying bubble plot (Supplementary Figure S16) visualized a clear inverse trend: younger mean ages strongly correlated with pronounced LDL-C elevations, a metabolic response that diminished progressively with advancing age.

### 3.5. Age-Specific Analysis of the Effects of Intermittent Fasting on Glycemic Metabolism Parameters

IF demonstrated potential, albeit inconsistent, benefits on glycemic metabolism. Significant reductions in fasting insulin (FINS) were driven by both the young (*p* = 0.004) and older adult cohorts (*p* = 0.045); however, a significant improvement in HOMA-IR was observed only in the young cohort (*p* = 0.035), with older adults showing a borderline reduction (*p* = 0.071). Early middle-aged adults demonstrated no significant changes in either marker. Fasting blood glucose (FBG) remained unaffected across all standard subgroup analyses (all *p* > 0.05. Supplementary Figures S26–S28).

Importantly, our comprehensive leave-one-out sensitivity analysis revealed that the glycemic improvements observed in the older adult cohort (≥45 years) were statistically fragile (Supplementary Table S2). Successive omission of specific heavily weighted trials altered the significance of FINS, FBG, and HOMA-IR estimates in this age group. This fragility underscores that glycemic adaptations in older populations are highly heterogeneous and largely driven by individual trial characteristics.

### 3.6. Age-Specific Analysis of the Effects of Intermittent Fasting on Blood Pressure

Blood pressure adaptations to IF were most prominent in middle-aged and older adults. IF significantly reduced systolic blood pressure (SBP) in the ≥45 years cohort (MD = −4.86 mmHg, 95% CI [−7.94, −1.78], *p* = 0.002), an effect that proved highly robust in sensitivity testing. However, no significant SBP reductions were observed in young (*p* = 0.191) or early middle-aged adults (*p* = 0.240). Furthermore, IF did not significantly lower diastolic blood pressure (DBP) across any of the age strata (all *p* > 0.05, Supplementary Figures S33 and S34). Sensitivity analysis identified the DBP estimate in middle-aged and older adults and the SBP estimate in young adults as fragile.

### 3.7. Subgroup Analysis by Intermittent Fasting Modality

To determine whether specific fasting modalities distinctly influence clinical outcomes, we performed predefined subgroup analyses comparing time-restricted feeding (TRF) with intermittent energy restriction protocols (IER, comprising ADF and the 5:2 diet) (Supplementary Figure S18–S21).

Regarding anthropometric outcomes, both TRF and IER elicited robust and significant reductions in body weight and fat mass (FM). Specifically for weight loss, TRF (MD = −1.58 kg, 95% CI [−2.16, −0.99]) and IER (MD = −3.02 kg, 95% CI [−5.63, −0.40]) were both highly effective, with no significant difference observed between the two modalities (*P_interaction_* = 0.290). A parallel efficacy was observed for FM reduction, where both TRF (MD = −1.30 kg, 95% CI [−1.49, −1.12]) and IER (MD = −1.79 kg, 95% CI [−2.27, −1.31]) successfully decreased adiposity (*P_interaction_* = 0.060).

For fat-free mass (FFM), both fasting modalities demonstrated significant lean mass loss. TRF interventions were associated with a statistically significant reduction in FFM (MD = −0.96 kg, 95% CI [−1.09, −0.82]), and a similarly significant reduction was observed in the IER cohort (MD = −1.39 kg, 95% CI [−1.85, −0.93]). This indicates that the risk of muscle mass depletion is a generalized response to intermittent fasting, irrespective of whether the caloric restriction is daily or periodic (*P_interaction_* = 0.080).

In terms of lipid profiles, both TRF and IER protocols were associated with significant elevations in low-density lipoprotein cholesterol (LDL-C). The magnitude of the LDL-C increase was 2.30 mg/dL (95% CI [0.48, 4.11]) for TRF and 1.15 mg/dL (95% CI [0.18, 2.12]) for IER. This robust consistency suggests that the adverse lipid adaptation is a generalized physiological response to fasting interventions rather than being specific to a single modality (*P_interaction_* = 0.280).

### 3.8. Methodological Sensitivity Analysis (Conservative Correlation Imputation)

To address potential methodological bias, we conducted a rigorous sensitivity analysis, re-calculating missing standard deviations for changes from baseline using a highly conservative correlation coefficient (r = 0.5). The detailed results are provided in Supplementary Table S5. Remarkably, this conservative assumption not only corroborated the robustness of our primary weight-loss findings but also unmasked a statistically significant increase in LDL-C among middle-aged and older adults (MD = 1.09 mg/dL, 95% CI [0.18, 2.00], *p* = 0.020). This previously obscured adverse lipid adaptation emerged after the conservative algorithm effectively down-weighted lower-quality trials, highlighting the critical need for lipid monitoring in older practitioners of IF.

### 3.9. Feasibility and Intervention Adherence (Dropout Rates)

To quantitatively evaluate the clinical feasibility and tolerability of IF across different age groups, we analyzed study dropout rates as a proxy for intervention adherence. The meta-analysis (20 studies, N = 1070) revealed no significant difference in the risk of dropout between the IF and control groups overall (Risk Ratio [RR] = 1.03, 95% CI [0.76, 1.41], *p* = 0.840; Supplementary Figure S17). Heterogeneity was negligible (I^2^ = 0.0%), and subgroup analysis demonstrated consistent adherence profiles across young, early middle-aged, and middle-aged and older adults (*P_interaction_* = 0.939). This indicates that IF protocols are generally as feasible and tolerable as conventional dietary regimens, regardless of age.

### 3.10. Publication Bias and Certainty of Evidence (GRADE)

Visual assessment of contour-enhanced funnel plots (Supplementary Figures S10–S15) and subsequent Egger’s regression tests (all *p* > 0.05) revealed no statistically significant evidence of publication bias across the assessed outcomes.

However, the GRADE assessment revealed that the certainty of evidence for the effects of IF was predominantly low to very low across all three age groups (Supplementary Table S3). The evidence was universally downgraded due to serious risks of bias, stemming from methodological concerns regarding randomization and the inherent lack of blinding in dietary trials. Evidence certainty was further downgraded across various cardiometabolic outcomes due to serious inconsistency (unexplained statistical heterogeneity) and imprecision (wide confidence intervals). These assessments emphasize that while IF presents distinct age-specific patterns of benefit and risk, these findings should be interpreted as hypothesis-generating and warrant confirmation through high-quality, large-scale RCTs.

## 4. Discussion

This study, via a rigorous age-stratified meta-analysis, is the first to systematically reveal significant age-specificity in the effects of intermittent fasting (IF) on body composition and cardiometabolic health. Our in-depth analyses demonstrate that age is a critical, yet previously overlooked, effect modifier determining the magnitude, direction, and heterogeneity of IF’s effects. These findings challenge the traditional “one-size-fits-all” paradigm of IF application and provide pivotal evidence for advancing precision nutritional interventions. Crucially, our subgroup analyses by fasting modality revealed that time-restricted feeding (TRF) and intermittent energy restriction (IER) elicited largely comparable physiological responses, suggesting that the observed metabolic adaptations are generalized effects of fasting rather than protocol-specific artifacts. It is important to emphasize that while our meta-analysis of clinical trials establishes robust associations, the biological mechanisms proposed herein are intended to provide a plausible theoretical framework and warrant further validation in basic science and translational research.

### 4.1. Body Composition: Age-Modulated Efficacy and the Risk of Sarcopenia

The present study confirms that IF effectively reduces body weight and BMI across all age groups, consistent with prevailing consensus [18,82]. However, age stratification uncovers distinct physiological adaptations and potential risks. In young adults, efficient metabolic adaptability enables significant fat reduction without profound heterogeneity. The underlying biological basis may involve highly sensitive metabolic reprogramming in youthful livers, such as the signaling pathway mediating preferential lipid oxidation recently elucidated in Nature [83].

Conversely, the most attenuated responses were observed in early middle-aged adults. This blunted efficacy likely stems from a complex interplay of lifestyle and hormonal factors unique to this cohort. Early middle-aged individuals frequently experience higher occupational stress and sleep disruption, which elevate cortisol levels and promote insulin resistance. Furthermore, chronic stress often triggers compensatory overeating during the designated eating windows, effectively neutralizing the intended caloric deficit [84]. At the same time, this age bracket is characterized by a period of relative reproductive and metabolic homeostasis, where preserved sex steroid baselines cushion the acute energy stresses imposed by caloric restriction. In contrast, the profound cardiometabolic shifts observed in the middle-aged and older adult cohort (≥45 years)—such as significant triglyceride and blood pressure reductions—align physiologically with the onset of the perimenopausal and postmenopausal transitions in women, alongside gradual andropause-related alterations in men. This distinct endocrine decline post 45 years accelerates visceral adiposity and cardiovascular vulnerability, rendering this older demographic highly sensitive to the energy-sensing and lipid-mobilizing pathways stimulated by intermittent fasting.

In middle-aged and older adults, IF exhibited pronounced fat-loss efficacy, likely because their higher baseline insulin resistance provides a greater margin for metabolic improvement [85]. Importantly, however, our findings highlight a critical risk associated with IF: the significant depletion of fat-free mass (FFM) in this aging cohort. While weight loss is generally metabolically favorable for populations with overweight or obesity, the concomitant loss of lean muscle represents a severe clinical hazard, as it accelerates the onset of age-related sarcopenia, physical frailty, and metabolic dysfunction. This phenomenon likely results from age-related anabolic resistance, which blunts the muscle-protective effects typically conferred by IF-induced growth hormone pulses [86,87,88]. To mitigate this risk, the implementation of IF in older demographics should not be performed in isolation. We strongly advocate for an “IF+” multimodal clinical strategy. Clinical guidelines should mandate that middle-aged and older adults undertaking IF must simultaneously ensure a daily protein intake of ≥1.2–1.5 g/kg to leverage the muscle protein synthesis effect, engage in resistance training targeting major muscle groups at least 2–3 times per week, and utilize dual-energy X-ray absorptiometry (DXA) for precise body composition monitoring rather than relying solely on total body weight scales [89,90]. This comprehensive approach ensures that weight loss is primarily driven by adiposity reduction while preserving vital lean mass.

### 4.2. Lipid Profiles: Unmasking the Universal Risk of Ldl-C Elevation

Our rigorous sensitivity analyses revealed unexpected and highly significant age-dependent lipid adaptations. While IF effectively reduced triglycerides (TG) in middle-aged and older adults—aligning with previous reports on the benefits of long-term IF [18]—a highly concerning trend of elevated low-density lipoprotein cholesterol (LDL-C) was identified, which was statistically significant specifically in the young (<30 years) and middle-aged and older (≥45 years) cohorts.

In young adults, IF consistently and robustly increased LDL-C. Mechanistically, this may reflect a robust “rebound” effect in metabolically flexible young livers. While lipid mobilization is efficient during fasting periods, the refeeding phase—particularly if characterized by high-glycemic carbohydrates or saturated fats—may trigger a compensatory surge in VLDL synthesis and subsequent LDL-C elevation. More strikingly, our conservative methodological sensitivity analysis (r = 0.5) successfully ‘de-noised’ the pooled data and unmasked a statistically significant increase in LDL-C among middle-aged and older adults as well, an adverse trend that was previously obscured. This physiological reality aligns with the age-related decline in hepatic LDL receptor expression and diminished lipid clearance capacity. These findings strongly dictate a critical clinical recommendation: regardless of age, IF implementation must prioritize dietary quality during eating windows and mandate regular lipid monitoring to mitigate latent cardiometabolic risks.

### 4.3. Glycemic Regulation and Blood Pressure: Heterogeneity and Fragility

A distinct “U-shaped” age effect was observed for glycemic parameters: IF significantly improved fasting insulin in young and middle-aged and older adults, but not in the early middle-aged cohort. This pattern provides a key clue for resolving contradictions in the existing literature (e.g., Wang et al. [11] vs. Pureza et al. [91]). Our analyses suggest that when pooling all age groups, the positive glycemic effects in young and middle-aged and older adults are likely diluted by the “null effect” in early middle-aged adults. As the largest beneficiaries, middle-aged and older adults may amplify the benefits of IF due to their pronounced baseline metabolic dysfunction. However, our leave-one-out sensitivity analysis explicitly identified these glycemic improvements in middle-aged and older adults as statistically fragile. This fragility indicates that glycemic adaptations in older populations are heavily context-dependent and driven by specific trial protocols (e.g., varying baseline medication use and baseline insulin resistance), precluding sweeping generalizations.

Regarding hemodynamics, IF significantly reduced both systolic and diastolic blood pressure exclusively in the older adult subgroup, which proved to be a robust finding. This pronounced reduction may be partially driven by baseline characteristics. As vascular stiffening naturally progresses with age, older participants are more likely to present with higher, or ‘high-normal’, baseline blood pressure compared to younger cohorts. Consequently, the significant improvements among older adults might reflect a degree of regression to the mean. Additionally, their age-stiffened vasculature renders them highly responsive to both the fluid and sodium shifts inherently associated with fasting interventions, as well as the pleiotropic antihypertensive mechanisms of IF, which encompass weight loss, enhanced insulin sensitivity, and reduced systemic inflammation [92].

### 4.4. Limitations

This meta-analysis has several critical limitations that warrant consideration. First, regarding methodological constraints, our reliance on study-level mean age for stratification introduces the potential for an ecological fallacy. A study with a mean age of 40 may seamlessly group participants aged 25 to 55, blurring the true metabolic distinctions between discrete age cohorts. This constraint underscores why our age-stratified conclusions should be viewed as hypothesis-generating. Second, as evidenced by our rigorous sensitivity and subgroup analyses, several outcomes exhibited substantial statistical fragility and residual heterogeneity, which could not be entirely resolved due to the limited number of studies within specific strata. Third, the generalizability of our findings is constrained by the predominantly healthy, albeit overweight/obese, nature of the enrolled cohorts, limiting direct extrapolation to populations with severe cardiometabolic comorbidities. Fourth, a notable limitation of our meta-analysis—and of the current IF literature at large—is the inability to ascertain sex-specific efficacy. The vast majority of included RCTs enrolled mixed-gender cohorts without providing sex-disaggregated outcome data. Consequently, we could not systematically isolate the independent influence of biological sex. Given the well-established dimorphism in regional fat deposition and hormonal regulation (e.g., the protective metabolic effects of estrogen in premenopausal women), it is highly probable that male and female bodies adapt differently to IF. Future RCTs must prioritize reporting sex-stratified data to fully elucidate these divergent physiological responses.

In summary, while this analysis reveals compelling age-dependent metabolic trajectories, the certainty of evidence for most outcomes was rated as low to very low according to GRADE criteria, primarily due to serious risks of bias (inherent in dietary trials lacking blinding) and statistical inconsistency. Therefore, these findings should not be interpreted as definitive clinical directives. Future research must prioritize high-quality, long-term individual patient data (IPD) meta-analyses to confirm these age-specific effects, refine the “IF+” paradigm, and elucidate the precise molecular mechanisms governing fasting-induced physiological adaptations.

## 5. Conclusions

In conclusion, this comprehensive, age-stratified meta-analysis suggests that intermittent fasting (IF) presents a viable intervention for weight management, yet its effects on body composition and cardiometabolic health are profoundly age-dependent. Young adults demonstrate efficient fat reduction; early middle-aged adults exhibit attenuated and variable responses, highlighting the necessity for highly personalized protocols; and middle-aged and older adults achieve the most pronounced fat loss and glycemic improvements, but face a critical risk of lean mass depletion, necessitating our proposed “IF+” strategy (combining adequate protein intake with resistance training). Importantly, our rigorous sensitivity analyses unmasked a generalized risk of LDL-C elevation across age cohorts, underscoring that IF must be coupled with vigilant lipid monitoring regardless of age.

However, according to GRADE criteria, the certainty of the current evidence remains low to very low, primarily constrained by inherent methodological limitations and statistical heterogeneity within the primary trials. Therefore, these compelling age-specific patterns should be interpreted as hypothesis-generating rather than definitive clinical directives. Future research must prioritize high-quality, long-term individual patient data (IPD) meta-analyses to validate these distinct metabolic trajectories, elucidate the underlying molecular mechanisms, and ultimately guide precision nutritional practice.

## Figures and Tables

**Figure 3:**
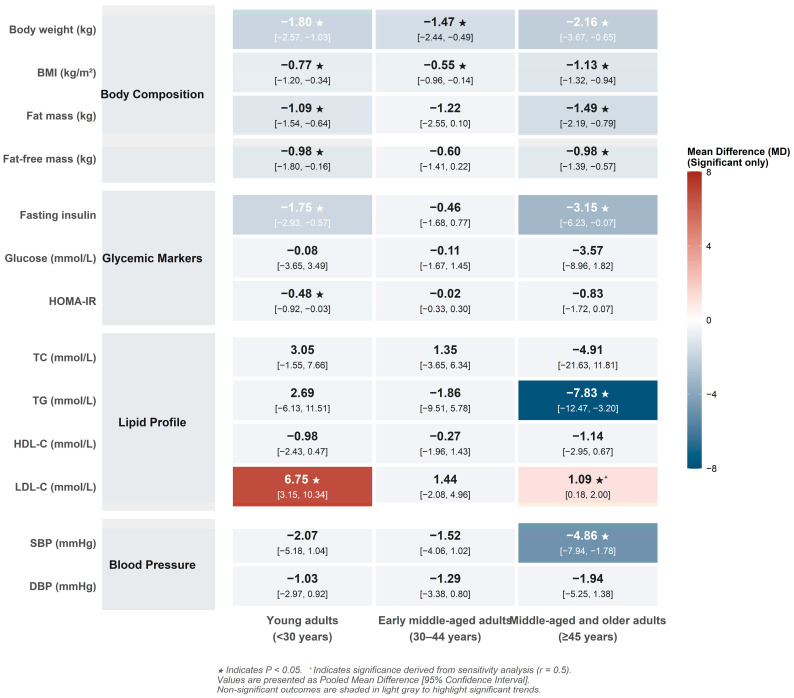
Comprehensive heatmap summarizing the age-stratified effects of intermittent fasting on body composition and cardiometabolic parameters. Cells display the pooled mean difference (MD) and 95% confidence intervals (CI) for each outcome across young (<30 years), early middle-aged (30–44 years), and middle-aged and older adults (≥45 years) cohorts. The color gradient quantitatively maps the magnitude and direction of statistically significant effect sizes: blue indicates a reduction, while red indicates an elevation (darker shades represent larger absolute effects). To intuitively highlight robust clinical trends, non-significant outcomes are intentionally muted in light gray. Stars (★) denote statistical significance (*p* < 0.05). The cross symbol (⁺) designates the significant LDL-C elevation in middle-aged and older adults unmasked during the conservative methodological sensitivity analysis (imputed correlation coefficient r = 0.5).

**Table 1:** Characteristics of participants and interventions.

Reference, Year	Sample Size (Biological Sex)	Population	Age (Years)	BMI (kg/m^2^)	Duration (Weeks)	IF Protocol	CON Protocol	Outcome	Main Findings
Cui et al., 2025 [57]	54 (F and M)	Overweight/Obese	TRF: 20.0 ± 1.1 CON: 20.0 ± 1.0	TRF: 26.3 ± 1.4 CON: 27.3 ± 3.4	8	10 h TRF	Usual diet	BW, FM, FFM, SBP, DBP	BW ↓, BMI ↓
Oldenburg et al., 2025 [58]	88 (F and M)	Overweight/Obese	TRF: 44.0 ± 11.5 CON: 43.4 ± 10.7	TRF: 35.8 ± 5.7 CON: 36.4 ± 4.1	12	8 h TRF (Self-selected window)	Usual diet	BW, FM, FFM, TG, HDL, LDL, FBG, FINS, HOMA-IR, SBP, DBP	BMI ↓
Rizvi et al., 2024 [60]	90 (F and M)	Overweight/Obese	TRF: 48.0 ± 8.3 CON: 43.4 ± 10.7	TRF: 29.95 ± 3.47 CON: 31.04 ± 4.68	12	8 h TRF (13:00–21:00)	Usual diet	BMI, TC, TG, HDL, LDL, SBP, DBP	TC ↓, LDL-C ↓
Sampieri et al., 2024 [59]	41 (F and M)	Healthy	16:8 diet: 41.5 ± 13.2 14:10 diet: 44.4 ± 15.3 12:12 diet: 38.1 ± 13.3 CON: 41.7 ± 13.8	16:8 diet: 26.8 ± 5.2 14:10 diet: 25.0 ± 3.0 12:12 diet: 24.7 ± 2.6 CON: 24.5 ± 3.8	8	16:8 TRF (10:00–18:00)/ 14:10 TRF (09:00–19:00)/ 12:12 TRF (08:00–20:00)	Usual diet	BW, FM, TC, TG, HDL, LDL, FBG, FINS, HOMA-IR	BW ↓, FINS ↑
Akasheh et al., 2024 [73]	49 (F and M)	Overweight/Obese	TRF: 47.2 ± 12.6 CON: 44.9 ± 20	TRF: 37.4 ± 6.3 CON: 35.9 ± 9.8	8	4 h TRF (15:00–19:00)/ 6 h TRF (13:00–19:00)	Usual diet	BW, BMI, FM, FFM, FBG, FINS, HOMA-IR	BW ↓, BMI ↓, FM ↓, FFM ↓, FINS ↓, HOMA-IR ↓
Lin et al., 2023 [55]	77 (F and M)	Overweight/Obese	TRF: 40.0 ± 12.0 CON: 44.0 ± 13.0	TRF: 37.0 ± 6.0 CON: 38.0 ± 5.0	48	8 h TRF (12:00–20:00)	Usual diet	BW, BMI, FM, FFM, TC, TG, HDL, LDL, FBG, FINS, HOMA-IR, SBP, DBP	BW ↓, BMI ↓, FM ↓
Xie et al., 2022 [62]	90 (F and M)	Healthy	eTRF: 28.7 ± 9.7 mTRF: 31.1 ± 8.4 CON: 33.57 ± 11.6	eTRF: 22.7 ± 3.1 mTRF: 21.4 ± 2.2 CON: 21.5 ± 2.9	5	8 h eTRF (06:00–15:00)/ 8 h mTRF (11:00–20:00)	Usual diet	BW, TC, TG, LDL, HDL, FBG, HOMA-IR, SBP, DBP	BW ↓, FM ↓, SBP ↓, DBP ↓
Isenmann et al., 2021 [63]	35 (FandM)	Healthy	TRF: 27.9 ± 5.3 CON: 27.4 ± 5.8	TRF: 26.3 ± 3.0 CON: 25.7 ± 3.3	8	8 h TRF	Usual diet	BW, BMI, FM	-
Lowe et al., 2020 [64]	116 (F and M)	Overweight/Obese	TRF: 46.8 ± 10.9 CMT: 46.1 ± 10.3	TRF: 32.9 ± 4.9 CMT: 32.6 ± 3.4	12	8 h TRF (12:00–20:00)	Usual diet	BW, FM, FFM, TC, TG, HDL, LDL, FBG, FINS, HOMA-IR, SBP, DBP	-
Moro et al., 2020 [56]	16 (M)	Healthy	TRF: 19.4 ± 2.4 CON: 19.4 ± 1.6	TRF: 21.9 ± 1.7 CON: 22.5 ± 1.8	4	8 h TRF (10:00–18:00)	Usual diet	BW, TC, TG, FBG, FINS	FBG ↓
Stekovic et al., 2019 [78]	90 (F and M)	Healthy	35–65	ADF: 25.5 ± 1.3 CON: 25.7 ± 1.3	4	Strict ADF (36 h fasting/12 h ad libitum)	Usual diet	BW, BMI, FM, FFM	BMI ↓, FM ↓, FFM ↓, SBP ↓
Cho et al., 2019 [77]	112 (F and M)	Overweight/Obese	ADF: 33.5 ± 5.0 CON: 42.6 ± 10.6	ADF: 27.8 ± 3.4 CON: 25.8 ± 3.4	8	ADF	Usual diet	BW, BMI, FBG, FINS, HOMA-IR	BW ↓, BMI ↓, FM ↓
Trepanowski et al., 2019 [82]	89 (F and M)	Overweight/Obese	ADF: 46.0 ± 2.0 CON: 44.0 ± 2.0	ADF: 34 ± 1 CON: 34 ± 1	12	Modified ADF (25% energy on fast days/125% on feast days)	Usual diet	FBG, FINS, HOMA-IR	-
Schübel et al., 2018 [75]	150 (F and M)	Overweight/Obese	ICR: 49.4 ± 9.0 CON: 5.7 ± 7.1	ICR: 32.0 ± 3.8 CON: 31.1 ± 3.6	12	5:2 Diet (25% energy on 2 non-consecutive days/week)	Usual diet	TC, TG, HDL, LDL, FBG, FINS, HOMA-IR	TC ↓, TG ↓, LDL-C ↑, FINS ↑, FBG ↑, HOMA-IR ↑
Betts et al., 2014 [67]	33 (F and M)	Healthy	IF: 36.0 ± 11.0 CON: 36.0 ± 11.0	IF: 22.8 ± 2.3 CON: 22.0 ± 2.2	6	Morning Fasting (No intake before 12:00)	Usual diet	BW, TC, TG, HDL, LDL, FBG, FINS, HOMA-IR	-
Chow et al., 2020 [66]	20 (F and M)	Overweight/Obese	TRE: 46.5 ± 12.4 CON: 44.2 ± 12.3	TRE: 33.8 ± 7.6 CON: 34.4 ± 7.8	12	8 h TRF (Self-selected window)	Usual diet	BW, FM, FFM, TG, HDL, LDL, FBG, FINS, HOMA-IR, SBP, DBP	TG ↓
Hirsh et al., 2019 [81]	22 (F and M)	Overweight/Obese	IER: 43.4 ± 13.0 CON: 39.0 ± 10.7	IER: 26.7 ± 1.9 CON: 27.7 ± 3.1	7	5:2 Diet (730 kcal/day on 2 days/week)	Usual diet	BW, TC, TG, HDL-C, LDL-C, FINS, SBP, DBP	FINS ↓
Stote et al., 2007 [68]	15 (F and M)	Healthy	TRF: 45.0 ± 2.7 CON: 45.0 ± 2.7	TRF: 23.4 ± 1.9 CON: 23.4 ± 1.9	8	OMAD/4 h TRF (17:00–21:00)	Usual diet	BW, FM, FFM, TG, TC, HDL, LDL, SBP, DBP	TC ↑, LDL-C ↑
Varady et al., 2013 [76]	30 (F and M)	Overweight/Obese	ADF: 47.0 ± 3.0 CON: 48.0 ± 2.0	ADF: 26.0 ± 1.0 CON: 26.0 ± 1.0	12	Modified ADF (25% energy on fast days)	Usual diet	BW, BMI, FM, FFM, TG, TC, HDL, LDL, SBP, DBP	TC ↓, TG ↓, SBP ↓
Domaszewski et al., 2022 [61]	116 (F and M)	Overweight/Obese	TRF: F: 69.7 ± 3.1 M: 68.1 ± 3.8 CON: F: 68.8 ± 3.45 M: 68.8 ± 3.4	TRF: F: 28.7 ± 4.0 M: 27.8 ± 1.8 CON: F: 27.3 ± 3.8 M: 27.9 ± 1.7	6	8 h TRF (12:00–20:00)	Usual diet	BW, BMI	BMI ↓
Oh et al., 2017 [74]	45 (F and M)	Overweight/Obese	ADCR: 32.9 ± 7.3 CON: 40.6 ± 10.0	ADCR: 27.6 ± 2.8 CON: 26.3 ± 3.0	8	Modified ADF (400–500 kcal on 3 alternate days/week)	Usual diet	BW, BMI, FM, TG, TC, HDL, FBG, FINS, HOMA-IR, SBP, DBP	BMI ↓
Liu et al., 2023 [69]	77 (F)	Overweight/Obese	TRF: 20.3 ± 1.8 CON: 20.1 ± 1.8	TRF: 21.6 ± 1.2 CON: 20.3 ± 1.1	8	8 h TRF (10:00–18:00)	Usual diet	BW, BMI, TC, TG, HDL, LDL, SBP, DBP	BW ↓, BMI ↓, TG ↑, LDL-C ↑
Witjaksono et al., 2022 [80]	50 (F and M)	Overweight/Obese	5:2 diet: 32.9 ± 8.4 CON: 32.1 ± 8.1	5:2 diet: 31.9 ± 4.2 CON: 31.5 ± 4.9	8	5:2 Diet (14 h fasting from dawn to dusk, 2 days/week)	Usual diet	BW, BMI, FM, FFM	-
Manoogian et al., 2022 [72]	150 (F and M)	Healthy	TRF: 41.1 ± 8.7 CON: 39.6 ± 9.4	TRF: 27.8 ± 3.7 CON: 27.7 ± 3.9	12	10 h TRF (Self-selected window)	Usual diet	BW, BMI, TC, LDL-C, HDL-C, FBG, HOMA-IR, SDP, DBP	-
Mena-Hernández et al., 2024 [71]	17 (F and M)	Overweight/Obese	TRF: 25.7 ± 10.0 CON: 25.7 ± 10.0	TRF: 32.0 ± 6.3 CON: 32.0 ± 6.3	4	8 h eTRF (07:00–15:00)	Usual diet	BW, BMI, FM, FFM, TC, TG, HDL, LDL, FBG, FINS, HOMA-IR, SBP, DBP	FBG ↑
Zhang et al., 2022 [70]	60 (F and M)	Overweight/Obese	eTRF: 23.8 ± 0.6 ITRF: 23.2 ± 0.5 CON: 22.1 ± 0.4	eTRF: 27.1 ± 0.7 ITRF: 28.5 ± 0.8 CON: 27.8 ± 0.8	8	6 h eTRF (07:00–13:00)	Usual diet	BW, BMI, FFM, TC, TG, HDL, LDL, FINS, FBG, HOMA-IR, SBP, DBP	BW ↓, BMI ↓, FM ↓, FFM ↓, FINS ↓, HOMA-IR ↓, SBP ↓,
Cienfuegos et al., 2020 [65]	58 (F and M)	Overweight/Obese	4-hTRF: 45.0 ± 2.0 6-hTRF: 46.0 ± 3.0 CON: 49.0 ± 2.0	4-hTRF: 36.0 ± 1.0 6-hTRF: 37.0 ± 1.0 CON: 36.0 ± 1.0	8	4 h TRF (15:00–19:00)/ 6 h TRF (13:00–19:00)	Usual diet	BW, BMI, FM, FFM, TC, TG, HDL, LDL, SBP, DBP	FM ↓, FFM ↓, TG ↓, HDL-C ↓, LDL-C ↑, FINS ↓, FBG ↓, HOMA-IR ↓, SBP ↓, DBP ↓
Gabel et al., 2019 [79]	43 (F and M)	Overweight/Obese	ADF: 43.0 ± 3.0 CON: 41.0 ± 3.0	ADF: 34.0 ± 1.0 CON: 35.0 ± 1.0	48	Modified ADF (25% energy on fast days/125% on feast days)	Usual diet	BW, BMI, FM, FFM, TC, TG, HDL, LDL, FINS, FBG, HOMA-IR, SBP, DBP	BW ↓, HOMA-IR ↓

Note: ↑ indicates a statistically significant increase; ↓ indicates a statistically significant decrease; - indicates no significant changes or not applicable.

**Table 2:** Summary of meta-analysis.

	Outcome	Trials	MD (95% CI)	*p* Value
IF vs. CON (<30 years)				
Body composition	Body weight	9	−1.80 [−2.57, −1.03]	<0.001
	BMI	7	−0.77 [−1.11, −0.44]	<0.001
	Fat mass	8	−1.09 [−1.54, −0.64]	<0.001
	Fat free mass	5	−0.98 [−1.80, −0.16]	0.020
Lipid profile	TC	6	3.05 [−1.55, 7.66]	0.194
	TG	6	2.69 [−6.13, 11.51]	0.550
	HDL-C	5	−0.98 [−2.43, 0.47]	0.187
	LDL-C	4	6.75 [3.15, 10.34]	<0.001
Glycemic markers	FINS	4	−1.75 [−2.93, −0.57]	0.004
	FBG	4	−0.08 [−3.65, 3.49]	0.964
	HOMA-IR	3	−0.48 [−0.92, −0.03]	0.035
Blood pressure	SBP	6	−2.07 [−5.18, 1.04]	0.191
	DBP	6	−1.03 [−2.97, 0.92]	0.301
IF vs. CON (30–44 years)				
Body composition	Body weight	7	−1.47 [−2.44, −0.49]	0.003
	BMI	6	−0.55 [−0.96, −0.14]	0.009
	Fat mass	5	−1.22 [−2.55, 0.10]	0.070
	Fat free mass	3	−0.60 [−1.41, 0.22]	0.154
Lipid profile	TC	6	1.35 [−3.65, 6.34]	0.598
	TG	7	−1.86 [−9.51, 5.78]	0.633
	HDL-C	8	−0.27 [−1.96, 1.43]	0.757
	LDL-C	8	1.44 [−2.08, 4.96]	0.424
Glycemic markers	FINS	7	−0.46 [−1.68, 0.77]	0.464
	FBG	8	−0.11 [−1.67, 1.45]	0.888
	HOMA-IR	9	−0.02 [−0.33, 0.30]	0.908
Blood pressure	SBP	5	−1.52 [−4.06, 1.02]	0.240
	DBP	5	−1.29 [−3.38, 0.80]	0.227
IF vs. CON (≥45 years)				
Body composition	Body weight	5	−2.16 [−3.67, −0.65]	0.005
	BMI	4	−1.13 [−1.32, −0.94]	<0.001
	Fat mass	6	−1.49 [−2.19, −0.79]	<0.001
	Fat free mass	6	−0.98 [−1.39, −0.57]	<0.001
Lipid profile	TC	4	−4.91 [−21.63, 11.81]	0.565
	TG	6	−7.83 [−12.47, −3.20]	<0.001
	HDL-C	6	−1.14 [−2.95, 0.67]	0.218
	LDL-C	6	0.19 [−4.32, 4.70]	0.934
Glycemic markers	FINS	4	−3.15 [−6.23, −0.07]	0.045
	FBG	4	−3.57 [−8.96, 1.82]	0.194
	HOMA-IR	4	−0.83 [−1.72, 0.07]	0.071
Blood pressure	SBP	6	−4.86 [−7.94, −1.78]	0.002
	DBP	5	−1.94 [−5.25, 1.38]	0.252

## Data Availability

No new data were created or analyzed in this study. Data sharing is not applicable to this article.

## Supplementary Materials

The following supporting information can be downloaded at https://www.mdpi.com/article/10.3390/nu18111799/s1, File S1a. PRISMA 2020 Main Checklist. File S1b. PRISMA 2020 for Abstracts Checklist. File S2. Search strategy. Table S1. Studies excluded after full text reading with the reason for exclusion [24,25,26,27,28,29,30,31,32,33,34,35,36,37,38,39,40,41,42,43,44,45,46,47,48,49,50,51,52,53,54]. Table S2. Leave-one-out sensitivity analyses. Table S3. Summary of findings based on GRADE assessment. Table S4. Risk of bias assessment table. Figure S1. Leave-one-out sensitivity analysis for body weight. Table S5. Methodological sensitivity analysis comparing standard versus conservative correlation coefficient assumptions. Figure S2. Leave-one-out sensitivity analysis for body mass index (BMI). Figure S3. Leave-one-out sensitivity analysis for fat mass (FM). Figure S4. Leave-one-out sensitivity analysis for fat-free mass (FFM). Figure S5. Leave-one-out sensitivity analysis for blood lipid profiles in middle-aged and older adults (≥45 years). Figure S6. Leave-one-out sensitivity analysis for fasting insulin (FINS). Figure S7. Leave-one-out sensitivity analysis for fasting blood glucose (FBG). Figure S8. Leave-one-out sensitivity analysis for HOMA-IR. Figure S9. Leave-one-out sensitivity analysis for blood pressure. Figure S10. Funnel plot of body weight for people aged < 30 years. Figure S11. Funnel plot of Fat mass for people aged < 30 years. Figure S12. Funnel plot of HDL-C for people aged 30–44 years. Figure S13. Funnel plot of LDL-C for people aged 30–44 years. Figure S14. Funnel plot of fasting glucose for people aged 30–44 years. Figure S15. Funnel plot of fasting insulin for people aged 30–44 years. Figure S16. Bubble plot of LDL-C for people aged < 30 years. Figure S17. Forest plot comparing dropout rates between intermittent fasting (IF) and control groups stratified by age. Figure S18. Subgroup analysis of intermittent fasting on body weight by intervention modality. Figure S19. Subgroup analysis of intermittent fasting on fat mass (FM) by intervention modality. Figure S20. Subgroup analysis of intermittent fasting on fat-free mass (FFM) by intervention modality. Figure S21. Subgroup analysis of intermittent fasting on low-density lipoprotein cholesterol (LDL-C) by intervention modality. Figure S22. Age-stratified forest plot of the effect of intermittent fasting on body weight. Figure S23. Age-stratified forest plot of the effect of intermittent fasting on body mass index (BMI). Figure S24. Age-stratified forest plot of the effect of intermittent fasting on fat mass (FM). Figure S25. Age-stratified forest plot of the effect of intermittent fasting on fat-free mass (FFM). Figure S26. Age-stratified forest plot of the effect of intermittent fasting on total cholesterol (TC). Figure S27. Age-stratified forest plot of the effect of intermittent fasting on triglycerides (TG). Figure S28. Age-stratified forest plot of the effect of intermittent fasting on high-density lipoprotein cholesterol (HDL-C). Figure S29. Age-stratified forest plot of the effect of intermittent fasting on low-density lipoprotein cholesterol (LDL-C). Figure S30. Age-stratified forest plot of the effect of intermittent fasting on fasting insulin (FINS). Figure S31. Age-stratified forest plot of the effect of intermittent fasting on fasting blood glucose (FBG). Figure S32. Age-stratified forest plot of the effect of intermittent fasting on homeostatic model assessment of insulin resistance (HOMA-IR). Figure S33. Age-stratified forest plot of the effect of intermittent fasting on systolic blood pressure (SBP). Figure S34. Age-stratified forest plot of the effect of intermittent fasting on diastolic blood pressure (DBP).

## Abbreviations

ADF, alternate-day fasting; BMI, body mass index; CI, confidence interval; CMT, consistent meal timing; CON, control; DBP, diastolic blood pressure; DXA, Dual-energy X-ray absorptiometry; F, female; FBG, fasting blood glucose; FFM, fat-free mass; FINS, fasting insulin; FM, fat mass; HDL-C, high-density lipoprotein cholesterol; HOMA-IR, homeostasis model assessment of insulin resistance; IF, intermittent fasting; IPD, individual patient data; LDL-C, low-density lipoprotein cholesterol; M, male; OMAD, one meal a day; RCT, randomized controlled trial; RoB2, Risk-of-Bias 2 tool; SBP, systolic blood pressure; SMD, standardized mean difference; TC, cholesterol; TG, triglyceride; TRF, time-restricted feeding (eTRF: early, mTRF: mid-day).
