# Supplementary material for: Age-Specific Analysis of the Effects of Intermittent Fasting on Body Composition and Cardiometabolic Markers in Healthy Adults and Individuals with Overweight or Obesity: A Systematic Review and Meta-Analysis of Randomized Controlled Trials
Source: Nutrients. 2026 Jun 3;18(11):1799. doi: 10.3390/nu18111799 (PMC13259446; doi:10.3390/nu18111799)

Age-Specific Analysis of the Effects of Intermittent Fasting on Body Composition and Cardiometabolic Markers in Healthy Adults and Individuals with Overweight or Obesity: A Systematic Review and Meta-Analysis of Randomized Controlled Trials

Kaijun Xing[1,2], Ruihan Liu[1,2], Shenglin Peng[1,2], Xuanxuan Zi[1,2], Linxi Lian[1,2], Bowen Yang[1,2],Yangyang Cen[1,2], Yichao Li[1,2], Yi Zhao[1,2,] and Yannan Zhang[1,2,*]

[1] School of Public Health, Ningxia Medical University, Yinchuan 750004, China.

[2] Ningxia Key Laboratory of Environmental Factors and Chronic Disease Control, Yinchuan 750004, China.

***** Correspondence: Yannan Zhang (yannan_jn@163.com)

Index:

ID	Subheading	Page number (s)
File S1a	PRISMA 2020 Main Checklist	1–3
File S1b	PRISMA 2020 for Abstracts Checklist	4
File S2	Search strategy for each database	5
Table S1	Studies excluded after full text reading with the reason for exclusion.	6–8
Table S2	Sensitivity analysis	9–11
Table S3	Summary of findings based on GRADE assessment.	12–22
Figures S1–S9	Forest plot after sensitivity analysis	23–29
Figures S10–S15	Funnel plot	30–35
Figure S16	Bubble plot	36
Figure S17	Forest plot comparing dropout rates	37
Table S4	Risk of bias assessment table	38–39
Figures S18–S21	Subgroup Analysis by Intermittent Fasting Protocol	40–43
Table S5	Methodological sensitivity analysis	44
Figures S22–S34	Age-stratified forest plot of the effect of intermittent fasting	44–57

File S1a. PRISMA 2020 Main Checklist.

Section and Topic	Item #	Checklist item	Location where item is reported
**TITLE**			
Title	1	Identify the report as a systematic review.	Title
**ABSTRACT**			
Abstract	2	See the PRISMA 2020 for Abstracts checklist.	Abstract
**INTRODUCTION**			
Rationale	3	Describe the rationale for the review in the context of existing knowledge.	2
Objectives	4	Provide an explicit statement of the objective(s) or question(s) the review addresses.	2
**METHODS**			
Eligibility criteria	5	Specify the inclusion and exclusion criteria for the review and how studies were grouped for the syntheses.	3–4
Information sources	6	Specify all databases, registers, websites, organisations, reference lists and other sources searched or consulted to identify studies. Specify the date when each source was last searched or consulted.	3
Search strategy	7	Present the full search strategies for all databases, registers and websites, including any filters and limits used.	3,Sup. Materials
Selection process	8	Specify the methods used to decide whether a study met the inclusion criteria of the review, including how many reviewers screened each record and each report retrieved, whether they worked independently, and if applicable, details of automation tools used in the process.	3
Data collection process	9	Specify the methods used to collect data from reports, including how many reviewers collected data from each report, whether they worked independently, any processes for obtaining or confirming data from study investigators, and if applicable, details of automation tools used in the process.	3–4
Data items	10a	List and define all outcomes for which data were sought. Specify whether all results that were compatible with each outcome domain in each study were sought (e.g. for all measures, time points, analyses), and if not, the methods used to decide which results to collect.	3
	10b	List and define all other variables for which data were sought (e.g. participant and intervention characteristics, funding sources). Describe any assumptions made about any missing or unclear information.	3, Table 1
Study risk of bias assessment	11	Specify the methods used to assess risk of bias in the included studies, including details of the tool(s) used, how many reviewers assessed each study and whether they worked independently, and if applicable, details of automation tools used in the process.	4, Figure 2
Effect measures	12	Specify for each outcome the effect measure(s) (e.g. risk ratio, mean difference) used in the synthesis or presentation of results.	4
Synthesis methods	13a	Describe the processes used to decide which studies were eligible for each synthesis (e.g. tabulating the study intervention characteristics and comparing against the planned groups for each synthesis (item #5)).	3–4

Section and Topic	Item #	Checklist item	Location where item is reported
	13b	Describe any methods required to prepare the data for presentation or synthesis, such as handling of missing summary statistics, or data conversions.	3–4
	13c	Describe any methods used to tabulate or visually display results of individual studies and syntheses.	4, Figure 3, Table 2
	13d	Describe any methods used to synthesize results and provide a rationale for the choice(s). If meta-analysis was performed, describe the model(s), method(s) to identify the presence and extent of statistical heterogeneity, and software package(s) used.	4–5
	13e	Describe any methods used to explore possible causes of heterogeneity among study results (e.g. subgroup analysis, meta-regression).	4–5
	13f	Describe any sensitivity analyses conducted to assess robustness of the synthesized results.	4–5
Reporting bias assessment	14	Describe any methods used to assess risk of bias due to missing results in a synthesis (arising from reporting biases).	5
Certainty assessment	15	Describe any methods used to assess certainty (or confidence) in the body of evidence for an outcome.	5
**RESULTS**			
Study selection	16a	Describe the results of the search and selection process, from the number of records identified in the search to the number of studies included in the review, ideally using a flow diagram.	5–6, Figure 1
	16b	Cite studies that might appear to meet the inclusion criteria, but which were excluded, and explain why they were excluded.	5, Table S1
Study characteristics	17	Cite each included study and present its characteristics.	6–7, Table 1
Risk of bias in studies	18	Present assessments of risk of bias for each included study.	7, Figure 2
Results of individual studies	19	For all outcomes, present, for each study: (a) summary statistics for each group (where appropriate) and (b) an effect estimate and its precision (e.g. confidence/credible interval), ideally using structured tables or plots.	13–16, Table 2, Figures S22–S34
Results of syntheses	20a	For each synthesis, briefly summarise the characteristics and risk of bias among contributing studies.	13–16
	20b	Present results of all statistical syntheses conducted. If meta-analysis was done, present for each the summary estimate and its precision (e.g.	13–16, Table 2,

Section and Topic	Item #	Checklist item	Location where item is reported
		confidence/credible interval) and measures of statistical heterogeneity. If comparing groups, describe the direction of the effect.	Figures S22–34
	20c	Present results of all investigations of possible causes of heterogeneity among study results.	17
	20d	Present results of all sensitivity analyses conducted to assess the robustness of the synthesized results.	17, Table S2, Figure S1–S9
Reporting biases	21	Present assessments of risk of bias due to missing results (arising from reporting biases) for each synthesis assessed.	17
Certainty of evidence	22	Present assessments of certainty (or confidence) in the body of evidence for each outcome assessed.	17
**DISCUSSION**			
Discussion	23a	Provide a general interpretation of the results in the context of other evidence.	17–19
	23b	Discuss any limitations of the evidence included in the review.	19–20
	23c	Discuss any limitations of the review processes used.	17–19
	23d	Discuss implications of the results for practice, policy, and future research.	20
**OTHER INFORMATION**			
Registration and protocol	24a	Provide registration information for the review, including register name and registration number, or state that the review was not registered.	2–3
	24b	Indicate where the review protocol can be accessed, or state that a protocol was not prepared.	3
	24c	Describe and explain any amendments to information provided at registration or in the protocol.	3
Support	25	Describe sources of financial or non-financial support for the review, and the role of the funders or sponsors in the review.	21
Competing interests	26	Declare any competing interests of review authors.	21

Section and Topic	Item #	Checklist item	Location where item is reported
Availability of data, code and other materials	27	Report which of the following are publicly available and where they can be found: template data collection forms; data extracted from included studies; data used for all analyses; analytic code; any other materials used in the review.	21

File S1b. PRISMA 2020 for Abstracts Checklist.

Section and Topic	Item #	Checklist item	Reported (Yes/No)
**TITLE**			
Title	1	Identify the report as a systematic review.	Yes
**BACKGROUND**			
Objectives	2	Provide an explicit statement of the main objective(s) or question(s) the review addresses.	Yes
**METHODS**			
Eligibility criteria	3	Specify the inclusion and exclusion criteria for the review.	Yes
Information sources	4	Specify the information sources (e.g. databases, registers) used to identify studies and the date when each was last searched.	Yes
Risk of bias	5	Specify the methods used to assess risk of bias in the included studies.	Yes
Synthesis of results	6	Specify the methods used to present and synthesise results.	Yes
**RESULTS**			
Included studies	7	Give the total number of included studies and participants and summarise relevant characteristics of studies.	Yes
Synthesis of results	8	Present results for main outcomes, preferably indicating the number of included studies and participants for each. If meta-analysis was done, report the summary estimate and confidence/credible interval. If comparing groups, indicate the direction of the effect (i.e. which group is favoured).	Yes
**DISCUSSION**			
Limitations of evidence	9	Provide a brief summary of the limitations of the evidence included in the review (e.g. study risk of bias, inconsistency and imprecision).	Yes
Interpretation	10	Provide a general interpretation of the results and important implications.	Yes
**OTHER**			
Funding	11	Specify the primary source of funding for the review.	No
Registration	12	Provide the register name and registration number.	Yes

File S2. Search strategy

Search strategy for PubMed search

(((Intermittent fasting[Title/Abstract] OR Intermittent energy intake[Title/Abstract] OR Intermittent energy restriction[Title/Abstract] OR Time restricted feeding[Title/Abstract] OR Time-restricted feeding[Title/Abstract] OR Time restricted eating[Title/Abstract] OR Time-restricted eating[Title/Abstract] OR Periodic fasting[Title/Abstract] OR Alternate-day fasting[Title/Abstract] OR Alternate day fasting[Title/Abstract])) AND ((Cardiometabolic risk factors[Title/Abstract] OR Body composition[Title/Abstract] OR Body weight[Title/Abstract] OR Waist circumference[Title/Abstract] OR Glucose[Title/Abstract] OR Insulin[Title/Abstract] OR Obesity[Title/Abstract] OR Blood pressure[Title/Abstract] OR Cholesterol, ldl[Title/Abstract] OR Cholesterol, hdl[Title/Abstract] OR Cholesterol[Title/Abstract]))) AND ((Randomized controlled trial[Title/Abstract] OR Comparative study[Title/Abstract] OR randomized controlled trial[Title/Abstract])))

Search strategy for Scopus

(TITLE-ABS-KEY (Intermittent fasting) OR TITLE-ABS-KEY (Intermittent energy intake) OR TITLE-ABS-KEY (Intermittent energy restriction) OR TITLE-ABS-KEY (Time restricted feeding) OR TITLE-ABS-KEY (Time-restricted feeding) OR TITLE-ABS-KEY (Time restricted eating) OR TITLE-ABS-KEY (Time-restricted eating) OR TITLE-ABS-KEY (Periodic fasting) OR TITLE-ABS-KEY (Alternate-day fasting) OR TITLE-ABS-KEY (Alternate day fasting) AND TITLE-ABS-KEY (Cardiometabolic risk factors) OR TITLE-ABS-KEY (Body composition) OR TITLE-ABS-KEY (Body weight) OR TITLE-ABS-KEY (Waist circumference) OR TITLE-ABS-KEY (Glucose) OR TITLE-ABS-KEY (Insulin) OR TITLE-ABS-KEY (Obesity) OR TITLE-ABS-KEY (Blood pressure) OR TITLE-ABS-KEY (Low density lipoprotein cholesterol) OR TITLE-ABS-KEY (High density lipoprotein cholesterol) OR TITLE-ABS-KEY (Cholesterol) AND TITLE-ABS-KEY (Randomized controlled trial) OR TITLE-ABS-KEY (Comparative study)) AND (LIMIT-TO (LANGUAGE , "English")))

Search strategy for Web of Science

(TS=((Intermittent fasting OR Intermittent energy intake OR Intermittent energy restriction OR Time restricted feeding OR Time-restricted feeding OR Time restricted eating OR Time-restricted eating OR Periodic fasting OR Alternate-day fasting OR Alternate day fasting OR Caloric restriction OR Continuous energy restriction OR Continuous caloric restriction OR Daily energy restriction)) AND TS=((Cardiometabolic risk factors OR Body composition OR Body weight OR Waist circumference OR Glucose OR Insulin OR Obesity OR Blood pressure OR Cholesterol, ldl OR Cholesterol, hdl OR Cholesterol)) AND TS=((Randomized controlled trial OR Comparative study))AND LA==("ENGLISH")))

Table S1. Studies excluded after full text reading with the reason for exclusion.

ID	Title	author	Reason for exclusion
1	Effects of early, late and self-selected time-restricted eating on visceral adipose tissue and cardiometabolic health in participants with overweight or obesity: a randomized controlled trial	Dote-Montero et al., 2025 [24]	Wrong intervention
2	Effects of Different Types of Intermittent Fasting Interventions on Metabolic Health in Healthy Individuals (EDIF): A Randomised Trial with a Controlled-Run in Phase	Herz et al., 2024 [25]	No mean outcome values
3	Effects of intermittent (5:2) or continuous energy restriction on basal and postprandial metabolism: a randomised study in normal weight, young participants	Gao et al., 2021 [26]	No conventional control
4	Calorie restriction with or without time-restricted eating in weight loss	Deying Liu et al., 2022 [27]	No conventional control
5	Intermittent energy restriction ameliorates adipose tissue-associated inflammation in adults with obesity: A randomised controlled tria	Inês Castela et al., 2022 [28]	No conventional control
6	Effect of time-restricted feeding on body composition and cardio metabolic risk in middle-aged women in Taiwan	Lin et al., 2021 [29]	Combination of IF with exercise training intervention
7	Intermittent and continuous energy restriction result in similar weight loss, weight loss maintenance, and body composition changes in a 6 month randomized pilot study	Steger et al., 2021 [30]	Combination of IF with exercise training intervention
8	Effectiveness of Early Time-Restricted Eating for Weight Loss,<br>Fat Loss, and Cardiometabolic Health in Adults With Obesity<br>A Randomized Clinical Trial	Jamshed et al., 2022 [31]	No conventional control
9	Dietary recommendations for fasting days in an alternate-day intermittent fasting pattern: a randomized controlled trial	Xu et al., 2022 [32]	No conventional control
10	A randomized controlled trial to isolate the effects of fasting and energy restriction on weight loss and metabolic health in lean adults	Templeman et al., 2021 [33]	No conventional control
11	Time-restricted eating and concurrent exercise training reduces fat mass and increases lean mass in overweight and obese adults	Kotarsky et al., 2021 [34]	< 3 outcomes of interest
12	Matched Weight Loss Through Intermittent<br>or Continuous Energy Restriction Does Not Lead To Compensatory Increases in Appetite<br>and Eating Behavior in a Randomized Controlled Trial in Women with Overweight and	Beaulieu et al., 2020 [35]	No conventional control

ID	Title	author	Reason for exclusion
	Obesity		
13	Intermittent energy restriction is comparable to continuous energy restriction for cardiometabolic health in adults with central obesity: a randomized controlled trial	Pinto et al., 2020 [36]	No conventional control
14	Compensatory mechanisms activated with intermittent energy restriction: a randomized control trial	Sílvia Ribeiro Coutinho et al., 2018 [37]	No conventional control
15	Intermittent v. continuous energy restriction: differential effects on postprandial glucose and lipid metabolism following matched weight loss in overweight/obese participants	Antoni et al., 2018 [38]	No conventional control
16	Effect of Alternate-Day Fasting on Weight Loss, Weight Maintenance, and Cardioprotection Among Metabolically Healthy Obese Adults: A Randomized Clinical Trial	John F. Trepanowski et al., 2017 [39]	No mean outcome values
17	A randomized pilot study comparing zero-calorie alternate-day fasting to daily caloric restriction in adults with obesity	Catenacci et al., 2016 [40]	No conventional control
18	Similar body composition outcomes following volumetric diet and time-restricted eating in middle-aged individuals: a 12-week randomized controlled trial	Cloos et al., 2025 [41]	No conventional control
19	Effects of time-restricted feeding on body weight, body composition and vital signs in low-income women with obesity: a 12-month randomized clinical trial	Pureza et al., 2022 [42]	No mean outcome values
20	Effect of intermittent compared to continuous energy restriction on weight loss and weight maintenance after 12 months in healthy overweight or obese adults	Headland et al., 2018 [43]	No conventional control
21	Comparative study of time-restricted eating on body composition and metabolic parameters in climacteric women with obesity: analysis of a pre-post intervention	Araújo et al., 2025 [44]	No conventional control
22	Intermittent fasting and protein pacing are superior to caloric restriction for weight and visceral fat loss	Arciero et al., 2023 [45]	No conventional control
23	The Influence of Intermittent Fasting on Selected Human Anthropometric Parameters	Čermáková et al., 2024 [46]	No conventional control
24	The effect of intermittent fasting diets on body weight and composition	Erdem et al., 2022 [47]	< 3 outcomes of interest
25	The effect of intermittent energy and carbohydrate restriction v. daily energy restriction on weight loss and metabolic disease risk markers in overweight women	Harvie et al., 2013 [48]	No conventional control

ID	Title	author	Reason for exclusion
26	The effects of intermittent or continuous energy restriction on weight loss and metabolic disease risk markers: a randomised trial in young overweight women	Harvie rt al., 2011 [49]	No conventional control
27	Alternate-day modified fasting diet improves weight loss, subjective sleep quality and daytime dysfunction in women with obesity or overweight: a randomized, controlled trial	Hooshiar et al., 2023 [50]	No conventional control
28	Effect of intermittent versus continuous calorie restriction on body weight and cardiometabolic risk markers in subjects with overweight or obesity and mild-to-moderate hypertriglyceridemia: a randomized trial	Maroofi et al., 2020 [51]	No conventional control
29	The effects of three weight management methods on body composition and serum lipids of overweight and obese people	Cai et al., 2022 [52]	No mean outcome values
30	The effect of intermittent energy restriction on weight loss and<br>diabetes risk markers in women with a history of gestational diabetes:<br>a 12-month randomized control trial	Gray et al., 2021 [53]	No conventional control
31	Is two days of intermittent energy restriction<br>per week a feasible weight loss approach in obese males? A randomised pilot study	Conley et al., 2018 [54]	No conventional control

Table S2. Leave-one-out sensitivity analyses.

Outcome	Age_Group	I2_Heterogeneity	Original_Pooled_MD	Original_P_value	Sensitivity_MD_Range	Robustness
Weight	Young (<30 y)	63.70%	–1.80 [–2.57, –1.03]	0	–2.06 to –1.60	Robust (No change)
Weight	Early middle–aged (30–44 y)	73.80%	–1.47 [–2.44, –0.49]	0.003	–1.86 to –1.09	Robust (No change)
Weight	Middle–aged and older (≥45 y)	55.50%	–2.16 [–3.67, –0.65]	0.005	–3.27 to –1.26	Robust (No change)
BMI	Young (<30 y)	60.60%	–0.77 [–1.11, –0.44]	0	–0.98 to –0.70	Robust (No change)
BMI	Early middle–aged (30–44 y)	74.70%	–0.55 [–0.96, –0.14]	0.009	–0.83 to –0.36	Robust (No change)
BMI	Middle–aged and older (≥45 y)	0.00%	–1.13 [–1.32, –0.94]	0	–1.17 to –0.89	Robust (No change)
FM	Young (<30 y)	28.60%	–1.09 [–1.54, –0.64]	0	–1.19 to –0.96	Robust (No change)
FM	Early middle–aged (30–44 y)	56.40%	–1.22 [–2.55, 0.10]	0.07	–1.63 to –0.82	Fragile (Significance altered)
FM	Middle–aged and older (≥45 y)	85.40%	–1.49 [–2.19, –0.79]	0	–1.72 to –1.29	Robust (No change)
FFM	Young (<30 y)	23.90%	–0.98 [–1.80, –0.16]	0.02	–1.17 to –0.56	Fragile (Significance altered)
FFM	Early middle–aged (30–44 y)	0.00%	–0.60 [–1.41, 0.22]	0.154	–0.82 to –0.24	Robust (No change)
FFM	Middle–aged and older (≥45 y)	75.50%	–0.98 [–1.39, –0.57]	0	–1.20 to –0.87	Robust (No change)
TC	Young (<30 y)	6.70%	3.05 [–1.55, 7.66]	0.194	2.01 to 4.91	Fragile (Significance altered)
TC	Early middle–aged (30–44 y)	0.00%	1.35 [–3.65, 6.34]	0.598	–0.92 to 2.04	Robust (No change)
TC	Middle–aged and older (≥45 y)	91.30%	–4.91 [–21.63, 11.81]	0.565	–14.09 to 2.24	Robust (No change)
TG	Young (<30 y)	9.20%	2.69 [–6.13, 11.51]	0.55	–2.86 to 5.22	Robust (No change)
TG	Early middle–	0.00%	–1.86 [–9.51, 5.78]	0.633	–3.51 to –	Robust (No

Outcome	Age_Group	I2_Heterogeneity	Original_Pooled_MD	Original_P_value	Sensitivity_MD_Range	Robustness
	aged (30–44 y)				1.01	change)
TG	Middle–aged and older (≥45 y)	69.80%	–7.83 [–12.47, –3.20]	0.001	–10.13 to –6.19	Robust (No change)
HDL–C	Young (<30 y)	4.60%	–0.98 [–2.43, 0.47]	0.187	–1.46 to –0.59	Robust (No change)
HDL–C	Early middle–aged (30–44 y)	5.80%	–0.27 [–1.96, 1.43]	0.757	–0.61 to 0.17	Robust (No change)
HDL–C	Middle–aged and older (≥45 y)	72.00%	–1.14 [–2.95, 0.67]	0.218	–1.92 to –0.50	Fragile (Significance altered)
LDL–C	Young (<30 y)	0.00%	6.75 [3.15, 10.34]	0	6.12 to 7.32	Robust (No change)
LDL–C	Early middle–aged (30–44 y)	0.00%	1.44 [–2.08, 4.96]	0.424	0.59 to 2.23	Robust (No change)
LDL–C	Middle–aged and older (≥45 y)	79.40%	0.19 [–4.32, 4.70]	0.934	–1.03 to 1.84	Robust (No change)
FINS	Young (<30 y)	0.00%	–1.75 [–2.93, –0.57]	0.004	–2.47 to –1.38	Robust (No change)
FINS	Early middle–aged (30–44 y)	53.60%	–0.46 [–1.68, 0.77]	0.464	–0.79 to –0.00	Robust (No change)
FINS	Middle–aged and older (≥45 y)	91.80%	–3.15 [–6.23, –0.07]	0.045	–4.98 to –2.34	Fragile (Significance altered)
FBG	Young (<30 y)	68.70%	–0.08 [–3.65, 3.49]	0.964	–1.59 to 1.15	Robust (No change)
FBG	Early middle–aged (30–44 y)	0.00%	–0.11 [–1.67, 1.45]	0.888	–0.35 to 0.13	Robust (No change)
FBG	Middle–aged and older (≥45 y)	95.70%	–3.57 [–8.96, 1.82]	0.194	–5.82 to –2.32	Fragile (Significance altered)
HOMA–IR	Young (<30 y)	0.00%	–0.48 [–0.92, –0.03]	0.035	–0.57 to –0.26	Fragile (Significance altered)
HOMA–IR	Early middle–aged (30–44 y)	54.40%	–0.02 [–0.33, 0.30]	0.908	–0.10 to 0.05	Robust (No change)
HOMA–IR	Middle–aged and older (≥45 y)	89.20%	–0.83 [–1.72, 0.07]	0.071	–1.43 to –0.08	Fragile (Significance altered)
SBP	Young (<30 y)	67.30%	–2.07 [–5.18, 1.04]	0.191	–3.00 to –1.28	Robust (No change)

Outcome	Age_Group	I2_Heterogeneity	Original_Pooled_MD	Original_P_value	Sensitivity_MD_Range	Robustness
SBP	Early middle–aged (30–44 y)	0.00%	–1.52 [–4.06, 1.02]	0.24	–2.26 to –1.33	Robust (No change)
SBP	Middle–aged and older (≥45 y)	71.50%	–4.86 [–7.94, –1.78]	0.002	–6.45 to –3.60	Fragile (Significance altered)
DBP	Young (<30 y)	34.00%	–1.03 [–2.97, 0.92]	0.301	–1.59 to –0.50	Robust (No change)
DBP	Early middle–aged (30–44 y)	0.00%	–1.29 [–3.38, 0.80]	0.227	–2.33 to –0.72	Robust (No change)
DBP	Middle–aged and older (≥45 y)	80.80%	–1.94 [–5.25, 1.38]	0.252	–3.69 to –0.74	Fragile (Significance altered)

Table S3. Summary of findings based on GRADE assessment.

≤ 30 years:

Certainty assessment							№ of patients		Effect		Certainty	Importance
**№ of studies**	**Study design**	**Risk of bias**	**Inconsistency**	**Indirectness**	**Imprecision**	**Other considerations**	**IF**	**Control**	**Relative (95% CI)**	**Absolute (95% CI)**		
**Body weight**												
11	randomised trials	serious[a]	serious[b]	not serious	not serious	none	216	206	-	MD **1.8 lower** (2.5 lower to 1.1 lower)	⊕⊕◯◯ Low[a,b]	CRITICAL
**FM**												
8	randomised trials	serious[a]	serious[b]	not serious	not serious	none	208	198	-	MD **1.13 lower** (1.59 lower to 0.67 lower)	⊕⊕◯◯ Low[a,b]	CRITICAL
**FFM**												

Certainty assessment							№ of patients		Effect		Certainty	Importance
№ of studies	Study design	Risk of bias	Inconsistency	Indirectness	Imprecision	Other considerations	IF	Control	Relative (95% CI)	Absolute (95% CI)		
5	randomised trials	serious[a]	not serious	not serious	not serious	none	117	111	-	MD **0.98 lower** (1.82 lower to 0.14 lower)	⊕⊕⊕◯ Moderate[a]	CRITICAL
**TG**												
6	randomised trials	serious[a]	not serious	not serious	very serious[c]	none	156	150	-	MD **1.87 higher** (6.37 lower to 10.12 higher)	⊕◯◯◯ Very low[a,c]	IMPORTANT
**LDL-C**												
4	randomised trials	serious[a]	not serious	not serious	serious[d]	none	135	132	-	MD **6.53 higher** (3.49 higher to 9.58 higher)	⊕⊕◯◯ Low[a,d]	CRITICAL
**FINS**												

Certainty assessment							№ of patients		Effect		Certainty	Importance
**№ of studies**	**Study design**	**Risk of bias**	**Inconsistency**	**Indirectness**	**Imprecision**	**Other considerations**	**IF**	**Control**	**Relative (95% CI)**	**Absolute (95% CI)**		
4	randomised trials	serious[a]	not serious	not serious	not serious	none	77	71	-	MD **1.92 lower** (3.01 lower to 0.83 lower)	⨁⨁⨁◯ Moderate[a]	CRITICAL
**SBP**												
8	randomised trials	serious[a]	very serious[b]	not serious	very serious[c]	none	165	156	-	MD **2.25 lower** (5.4 lower to 0.9 higher)	⨁◯◯◯ Very low[a,b,c]	CRITICAL

CI: confidence interval; **MD:** mean difference

Explanations

a. Most studies had methodological limitations in the randomization process and/or intervention adherence (overall risk of bias: some concerns).

b. The pooled analysis revealed significant statistical heterogeneity

c. The 95% confidence interval for the effect estimate is wide, and it crosses the non-clinically significant threshold.

d. The 95% CI for the effect estimate is relatively wide.

30-45 years:

Certainty assessment							№ of patients		Effect		Certainty	Importance
**№ of studies**	**Study design**	**Risk of bias**	**Inconsistency**	**Indirectness**	**Imprecision**	**Other considerations**	**IF**	**Control**	**Relative (95% CI)**	**Absolute (95% CI)**		
**Body weight**												
7	randomised trials	serious[a]	serious[b]	not serious	not serious	none	231	242	-	MD **1.44 lower** (2.4 lower to 0.49 lower)	⊕⊕◯◯ Low[a,b]	CRITICAL
**FM**												

Certainty assessment							№ of patients		Effect		Certainty	Importance
**№ of studies**	**Study design**	**Risk of bias**	**Inconsistency**	**Indirectness**	**Imprecision**	**Other considerations**	**IF**	**Control**	**Relative (95% CI)**	**Absolute (95% CI)**		
5	randomised trials	serious[a]	serious[b]	not serious	very serious[c]	none	126	136	-	MD **1.18 lower** (2.41 lower to 0.06 higher)	⊕◯◯◯ Very low[a,b,c]	CRITICAL
**FFM**												
3	randomised trials	serious[a]	not serious	not serious	very serious[c]	none	117	120	-	MD **0.6 lower** (1.41 lower to 0.22 higher)	⊕◯◯◯ Very low[a,c]	CRITICAL
**TG**												

Certainty assessment							№ of patients		Effect		Certainty	Importance
№ of studies	Study design	Risk of bias	Inconsistency	Indirectness	Imprecision	Other considerations	IF	Control	Relative (95% CI)	Absolute (95% CI)		
7	randomised trials	serious[a]	not serious	not serious	very serious[c]	none	231	232	-	MD **2.49 lower** (9.72 lower to 4.74 higher)	⊕◯◯◯ Very low[a,c]	IMPORTANT

LDL-C

8	randomised trials	serious[a]	not serious	not serious	very serious[c]	none	240	244	-	MD **1.33 higher** (2.14 lower to 4.8 higher)	⊕◯◯◯ Very low[a,c]	CRITICAL

FINS

Certainty assessment							№ of patients		Effect		Certainty	Importance
**№ of studies**	**Study design**	**Risk of bias**	**Inconsistency**	**Indirectness**	**Imprecision**	**Other considerations**	**IF**	**Control**	**Relative (95% CI)**	**Absolute (95% CI)**		
7	randomised trials	serious[a]	serious[b]	not serious	very serious[c]	none	276	278	-	MD **0.4 lower** (1.63 lower to 0.83 higher)	⨁◯◯◯ Very low[a,b,c]	CRITICAL
**SBP**												
5	randomised trials	serious[a]	not serious	not serious	very serious[c]	none	181	181	-	MD **1.52 lower** (4.06 lower to 1.02 higher)	⨁◯◯◯ Very low[a,c]	CRITICAL

CI: confidence interval; **MD:** mean difference

Explanations
a. Most studies had methodological limitations in the randomization process and/or intervention adherence (overall risk of bias: some concerns).

b. The pooled analysis revealed significant statistical heterogeneity.

c. The 95% confidence interval for the effect estimate crosses the non-clinically significant threshold.

≥ 45 years

Certainty assessment							№ of patients		Effect		Certainty	Importance
**№ of studies**	**Study design**	**Risk of bias**	**Inconsistency**	**Indirectness**	**Imprecision**	**Other considerations**	**IF**	**Control**	**Relative (95% CI)**	**Absolute (95% CI)**		
**Body weight**												
5	randomised trials	serious[a]	serious[b]	not serious	not serious	none	199	178	-	MD **2.12 lower** (3.48 lower to 0.75 lower)	⨁⨁◯◯ Low[a,b]	CRITICAL
**FM**												

Certainty assessment							№ of patients		Effect		Certainty	Importance
№ of studies	Study design	Risk of bias	Inconsistency	Indirectness	Imprecision	Other considerations	IF	Control	Relative (95% CI)	Absolute (95% CI)		
6	randomised trials	serious[a]	very serious[b]	not serious	not serious	none	148	150	-	MD **1.51 lower** (2.24 lower to 0.78 lower)	⊕◯◯◯ Very low[a,b]	CRITICAL
**FFM**												
6	randomised trials	serious[a]	very serious[b]	not serious	not serious	none	148	150	-	MD **0.98 lower** (1.4 lower to 0.56 lower)	⊕◯◯◯ Very low[a,b]	CRITICAL
**TG**												
6	randomised trials	serious[a]	serious[b]	not serious	serious[c]	none	155	149	-	MD **7.71 lower** (12.23 lower to 3.18 lower)	⊕◯◯◯ Very low[a,b,c]	IMPORTANT

Certainty assessment							№ of patients		Effect		Certainty	Importance
№ of studies	Study design	Risk of bias	Inconsistency	Indirectness	Imprecision	Other considerations	IF	Control	Relative (95% CI)	Absolute (95% CI)		
**LDL-C**												
6	randomised trials	serious[a]	very serious[b]	not serious	serious[d]	none	155	149	-	MD **0.22 higher** (4.03 lower to 4.48 higher)	⨁◯◯◯ Very low[a,b,d]	CRITICAL
**FINS**												
4	randomised trials	serious[a]	very serious[b]	not serious	not serious	none	130	103	-	MD **3.2 lower** (5.93 lower to 0.46 lower)	⨁◯◯◯ Very low[a,b]	CRITICAL
**SBP**												

Certainty assessment							№ of patients		Effect		Certainty	Importance
**№ of studies**	**Study design**	**Risk of bias**	**Inconsistency**	**Indirectness**	**Imprecision**	**Other considerations**	**IF**	**Control**	**Relative (95% CI)**	**Absolute (95% CI)**		
6	randomised trials	serious[a]	serious[b]	not serious	not serious	none	136	157	-	MD **5.09 lower** (7.95 lower to 2.24 lower)	⊕⊕◯◯ Low[a,b]	CRITICAL

CI: confidence interval; **MD:** mean difference

Explanations

a. Most studies had methodological limitations in the randomization process and/or intervention adherence (overall risk of bias: some concerns).

b. The pooled analysis revealed significant statistical heterogeneity.

c. The 95% confidence interval for the effect estimate is wide.

d. The 95% confidence interval for the effect estimate crosses the non-clinically significant threshold.

Figure S1. Leave-one-out sensitivity analysis for body weight. Forest plots demonstrate the robustness of the pooled mean differences across three age cohorts: young adults (<30 years), early middle-aged adults (30–44 years) and middle-aged and older adults (≥45 years)

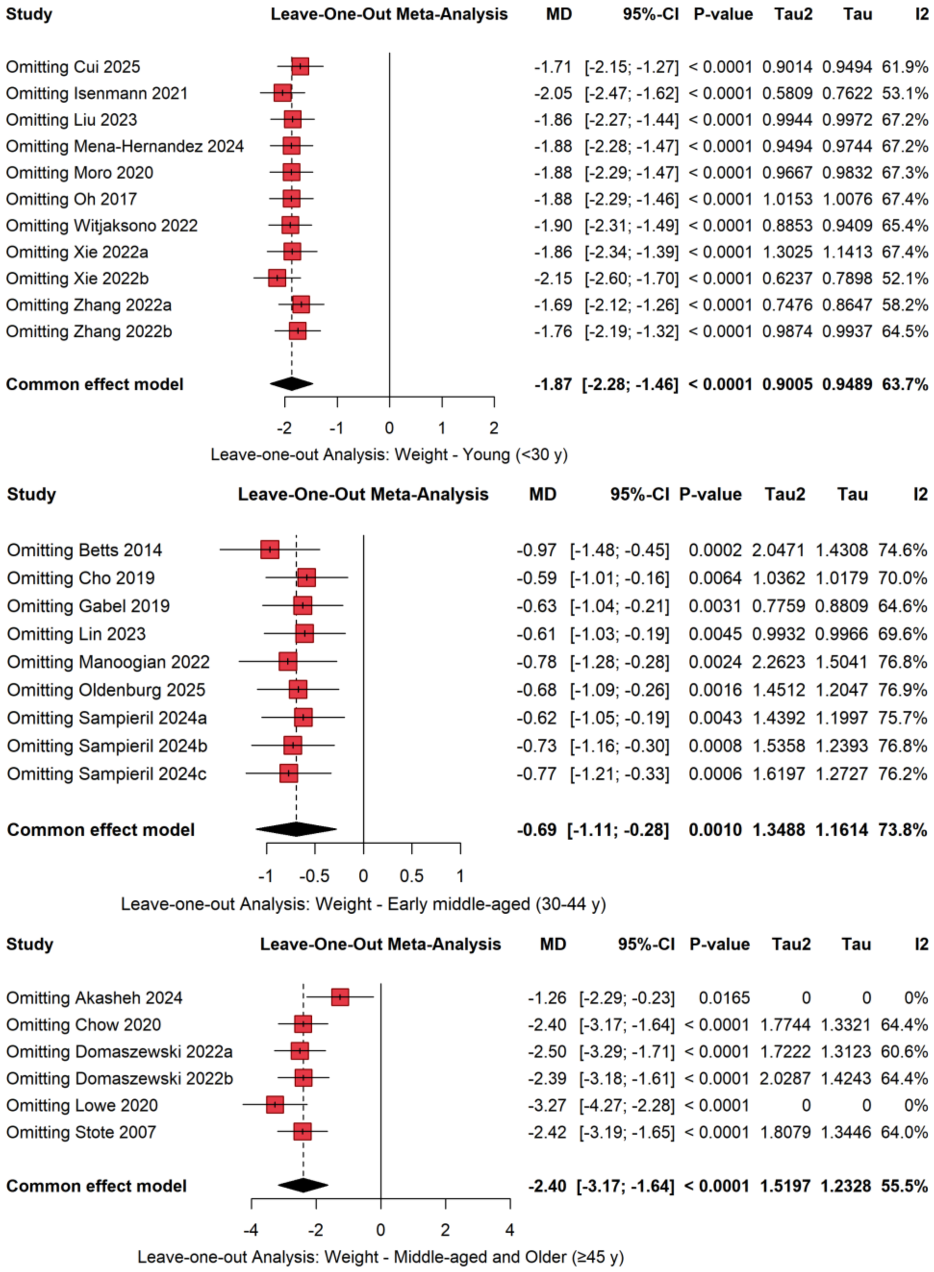

Figure S2. Leave-one-out sensitivity analysis for body mass index (BMI). Forest plots illustrate the sensitivity analysis for BMI reduction in young adults (<30 years) and early middle-aged adults (30–44 years).

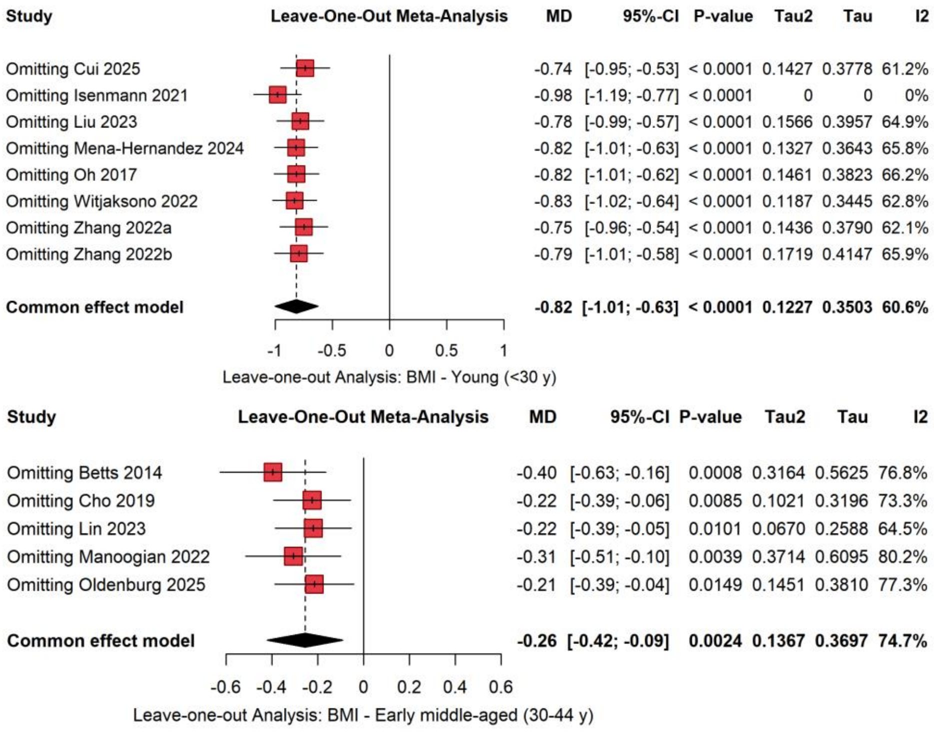

Figure S3. Leave-one-out sensitivity analysis for fat mass (FM). Forest plots assess the stability of the pooled effect on FM in early middle-aged adults (30–44 years) and middle-aged and older adults (≥45 years).

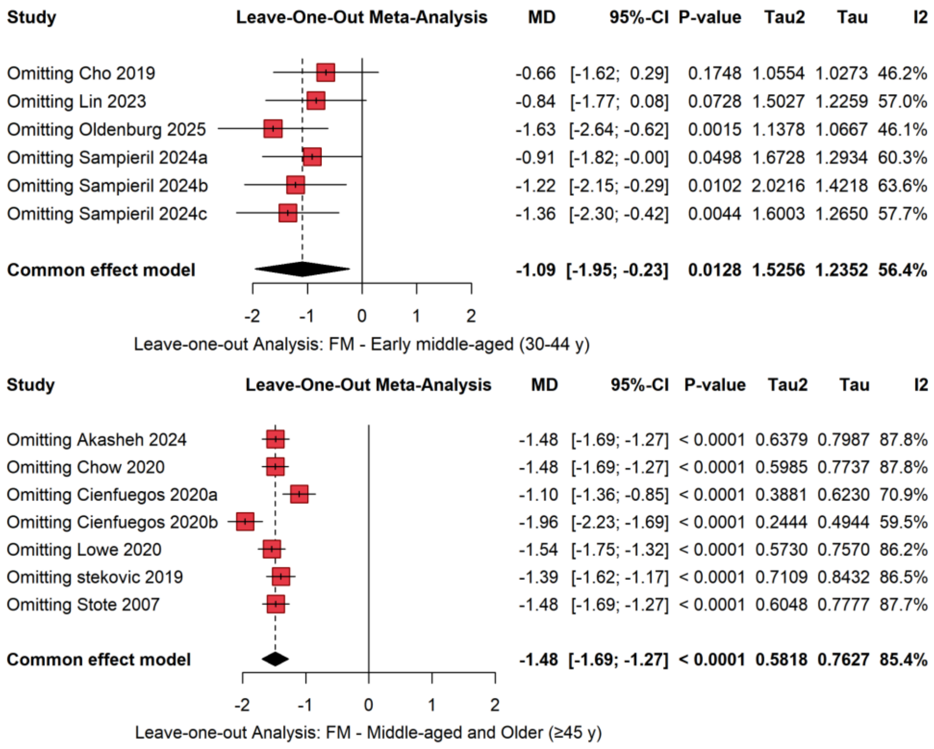

Figure S4. Leave-one-out sensitivity analysis for fat-free mass (FFM). Forest plot depicting the sensitivity analysis for FFM reduction in middle-aged and older adults (≥45 years).

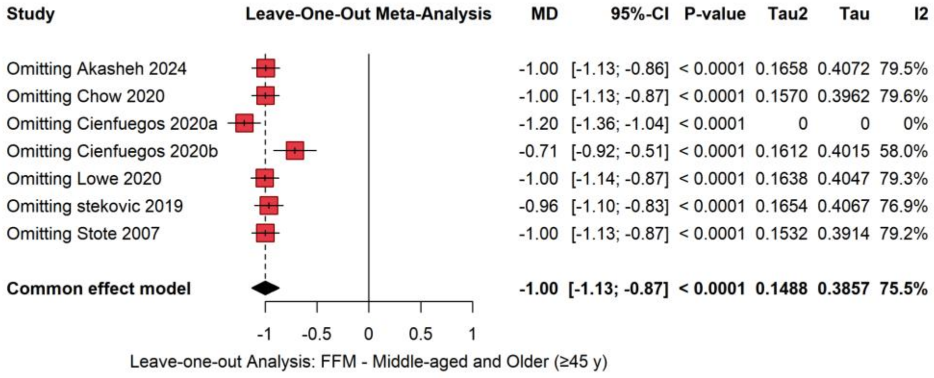

Figure S5. Leave-one-out sensitivity analysis for blood lipid profiles in middle-aged and older adults (≥45 years). Forest plots evaluate the stability of pooled estimates for various lipid markers: Total cholesterol (TC), Triglycerides (TG), High-density lipoprotein cholesterol (HDL-C), and Low-density lipoprotein cholesterol (LDL-C).

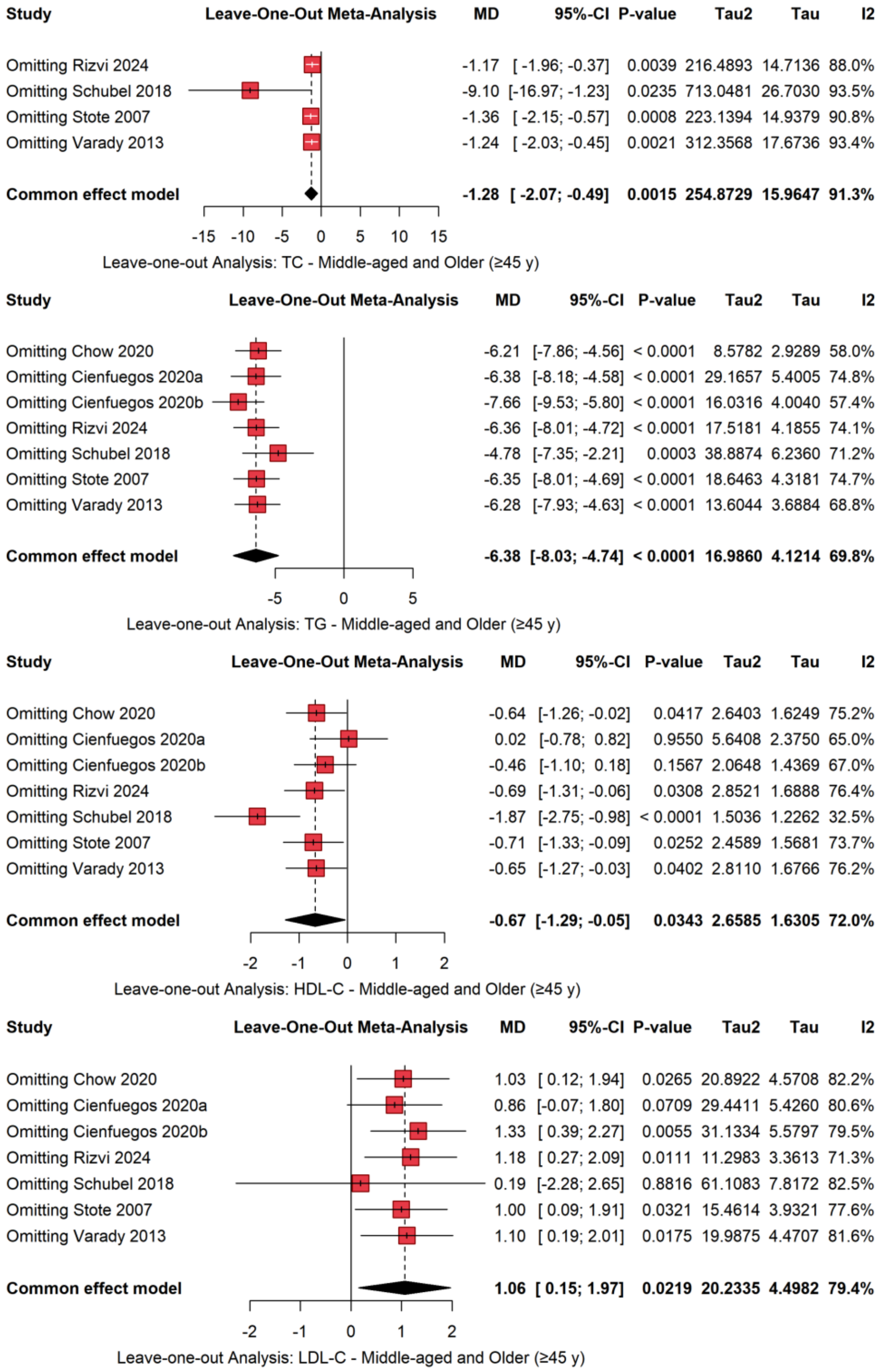

Figure S6. Leave-one-out sensitivity analysis for fasting insulin (FINS). Forest plots outline the sensitivity analysis for FINS in early middle-aged adults (30–44 years) and middle-aged and older adults (≥45 years).

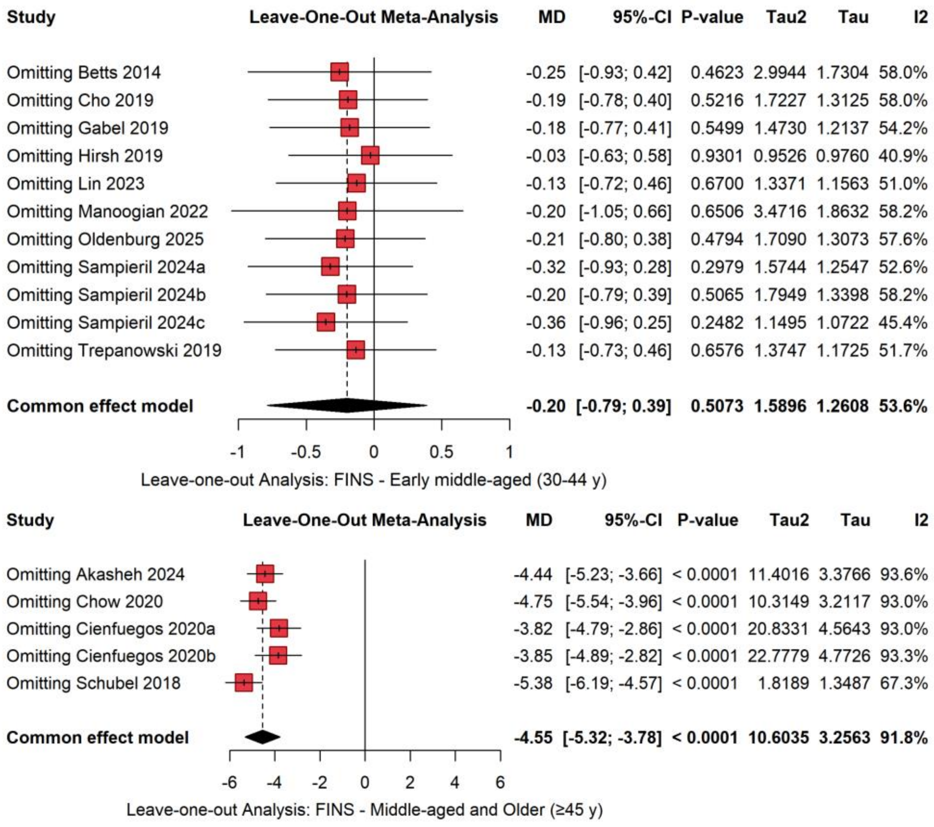

Figure S7. Leave-one-out sensitivity analysis for fasting blood glucose (FBG). Forest plots displaying the sensitivity analysis for FBG in young adults (<30 years) and middle-aged and older adults (≥45 years).

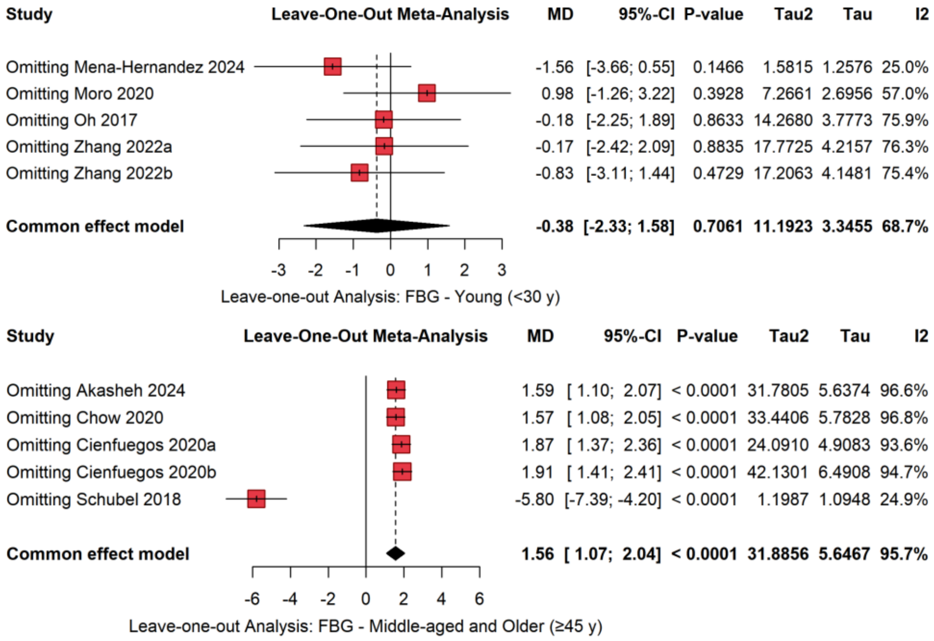

Figure S8. Leave-one-out sensitivity analysis for HOMA-IR. Forest plots indicating the robustness of HOMA-IR outcomes in early middle-aged adults (30–44 years) and middle-aged and older adults (≥45 years).

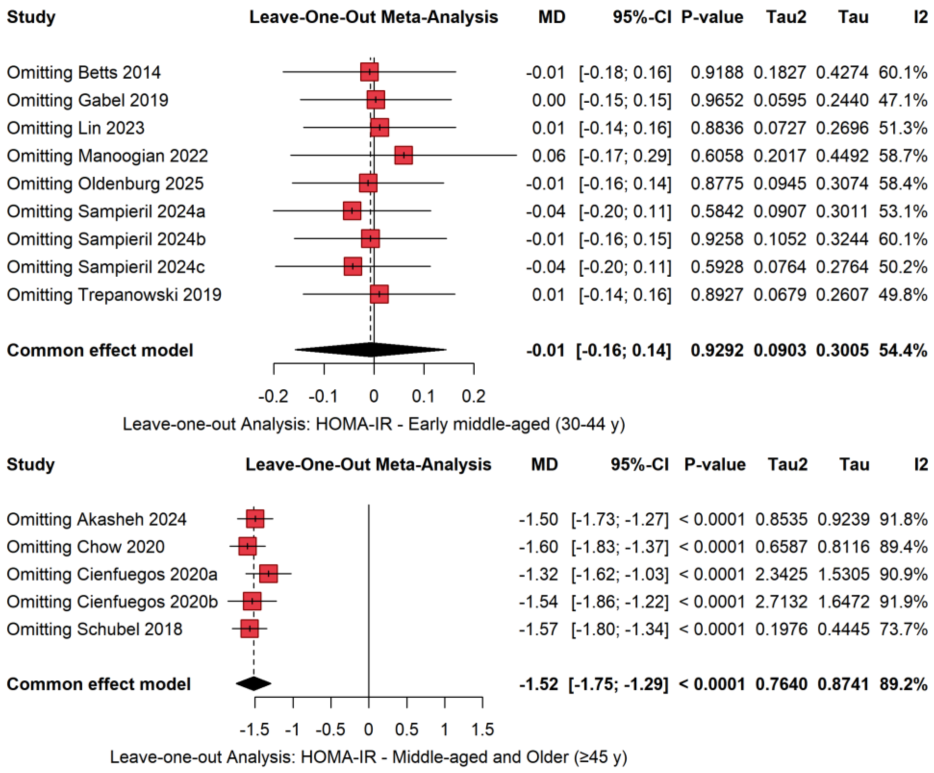

Figure S9. Leave-one-out sensitivity analysis for blood pressure. Forest plots summarizing the sensitivity analysis for Systolic blood pressure (SBP) in young adults (<30 years), SBP in middle-aged and older adults (≥45 years), and Diastolic blood pressure (DBP) in middle-aged and older adults (≥45 years).

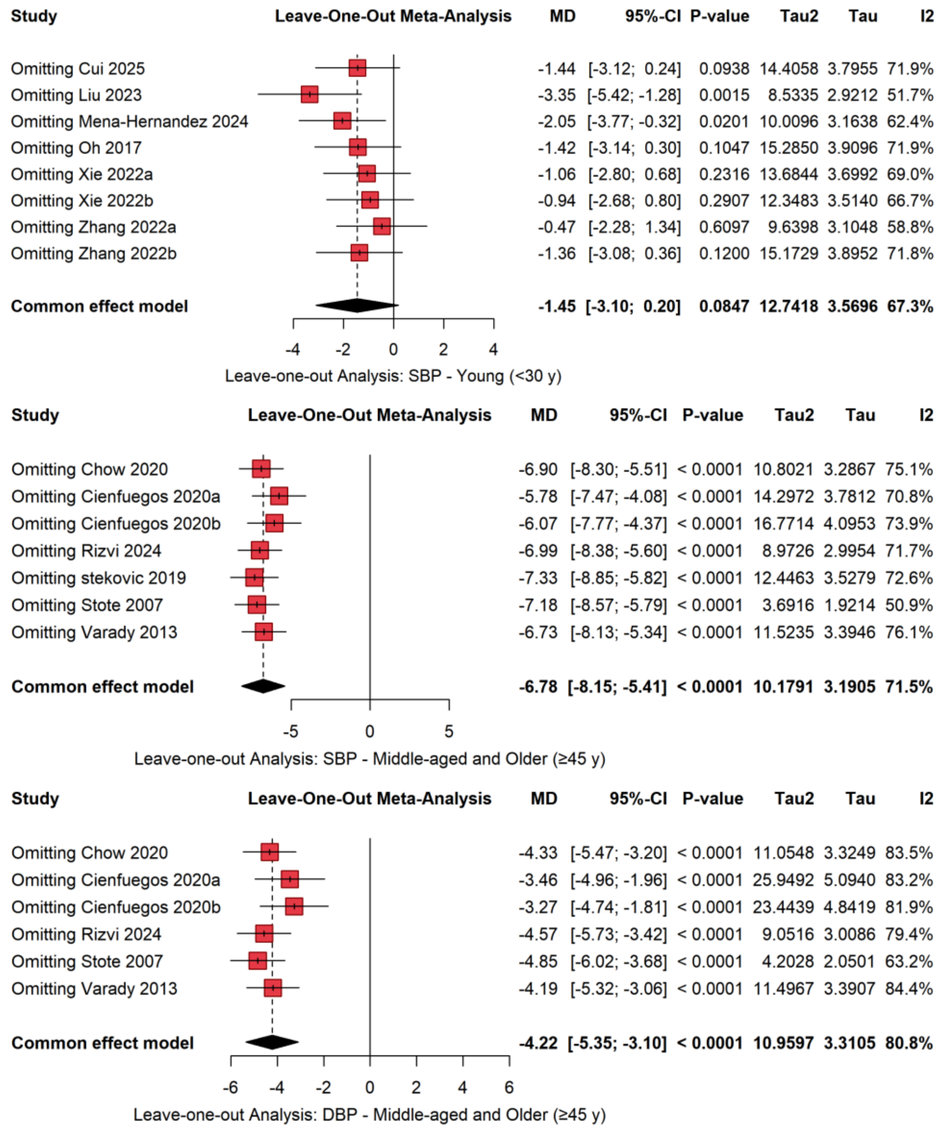

Figure S10. Funnel plot of body weight for people aged < 30 years.

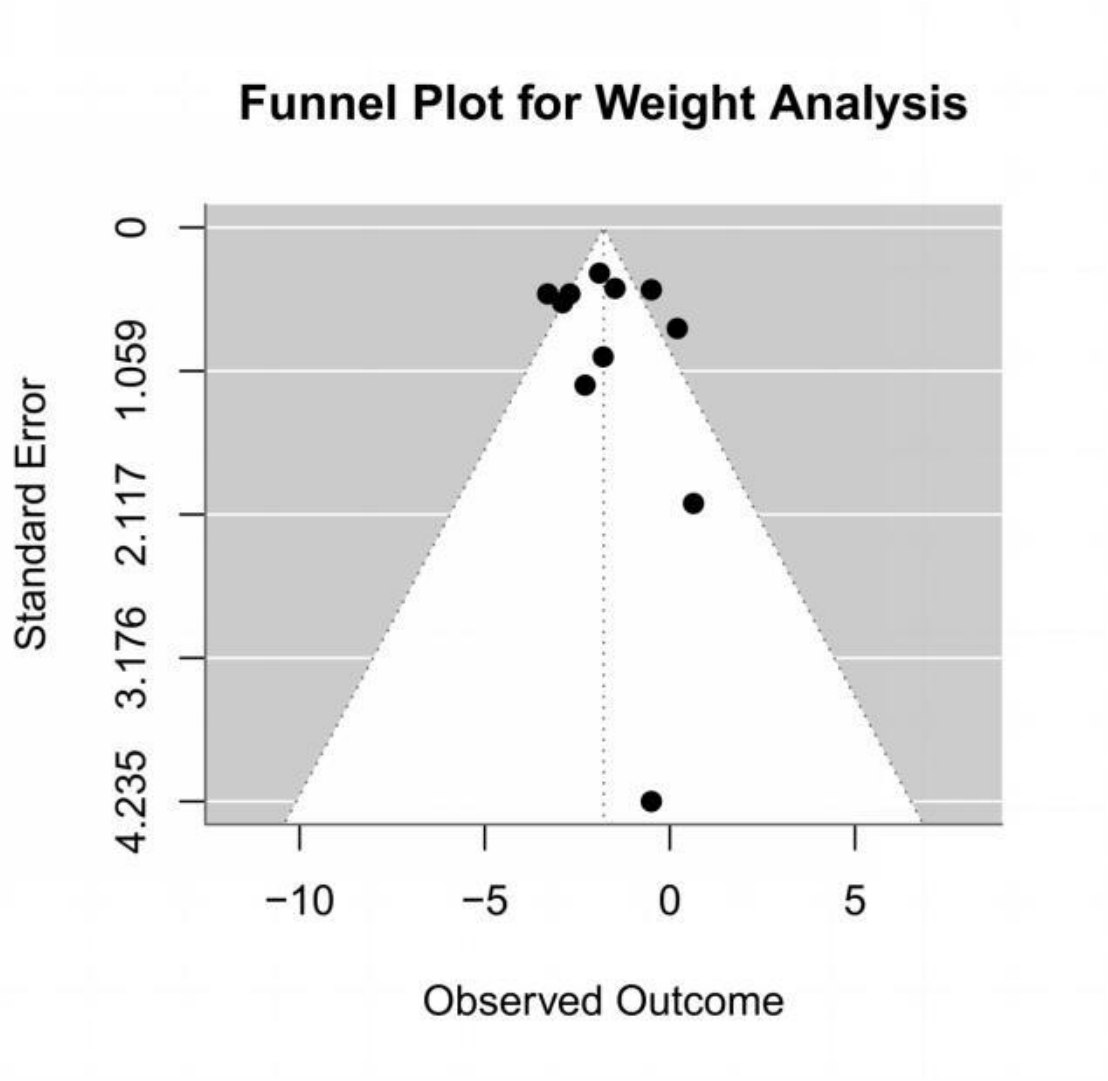

Figure S11. Funnel plot of Fat mass for people aged < 30 years.

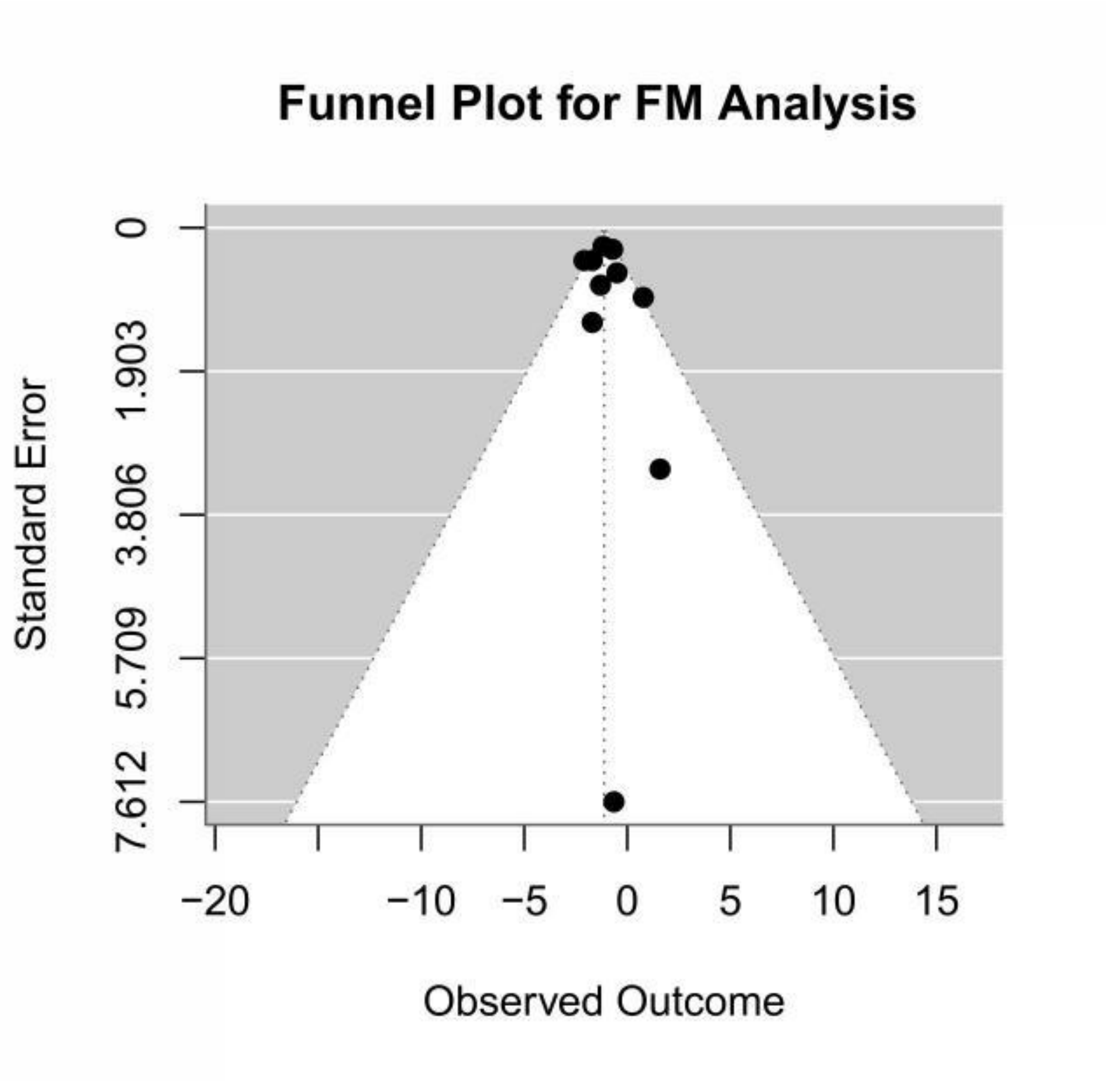

Figure S12. Funnel plot of HDL-C for people aged 30–44 years.

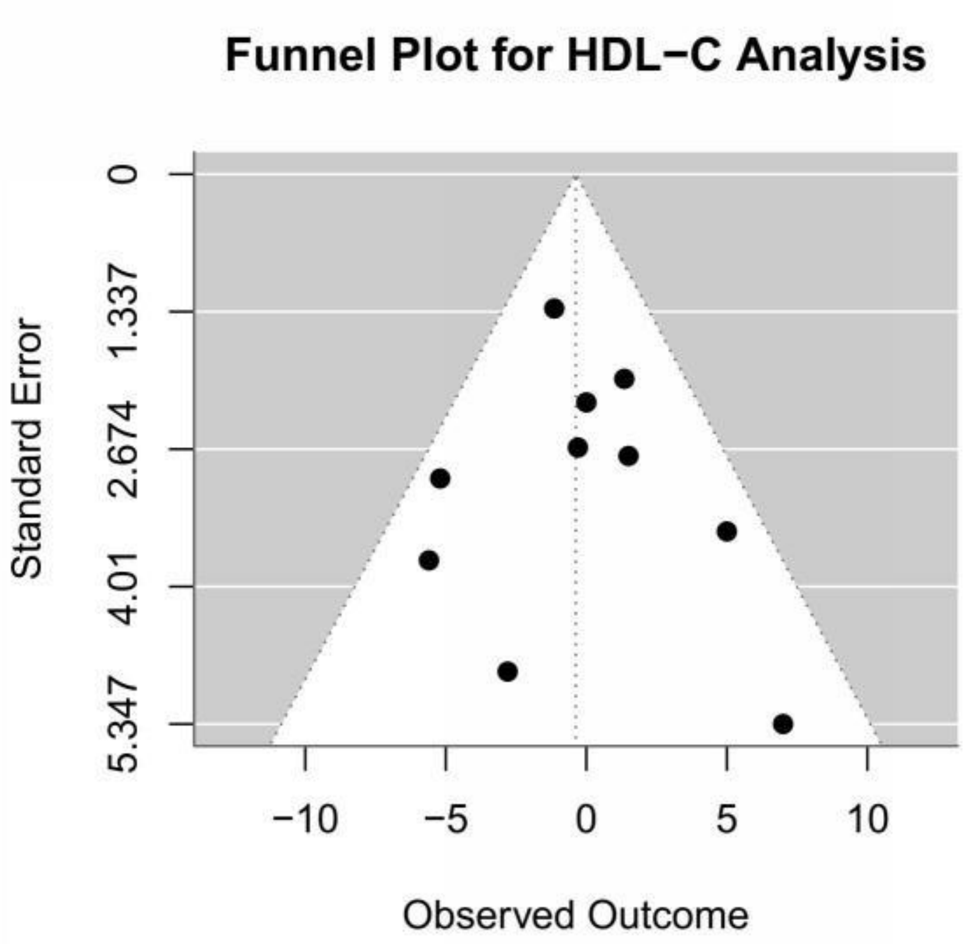

Figure S13. Funnel plot of LDL-C for people aged 30–44 years.

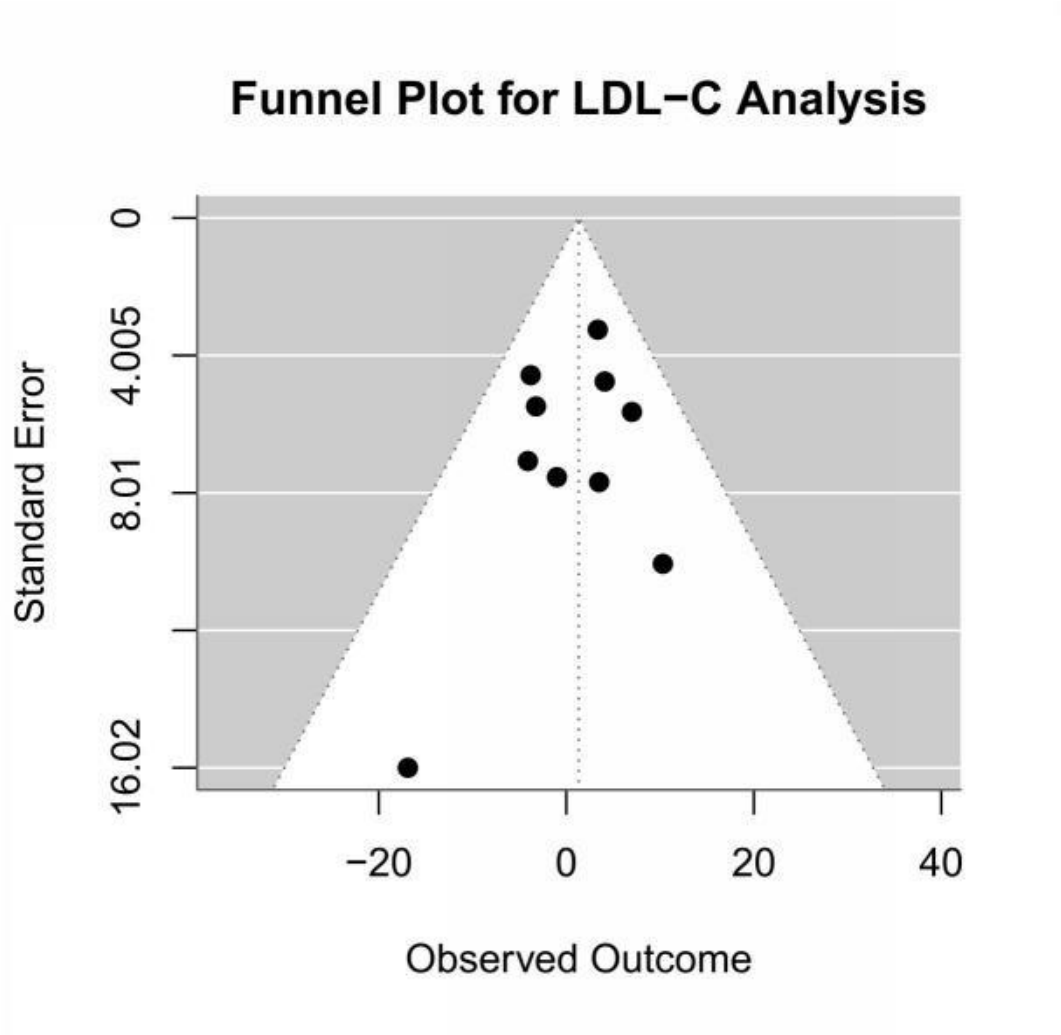

Figure S14. Funnel plot of fasting glucose for people aged 30–44 years.

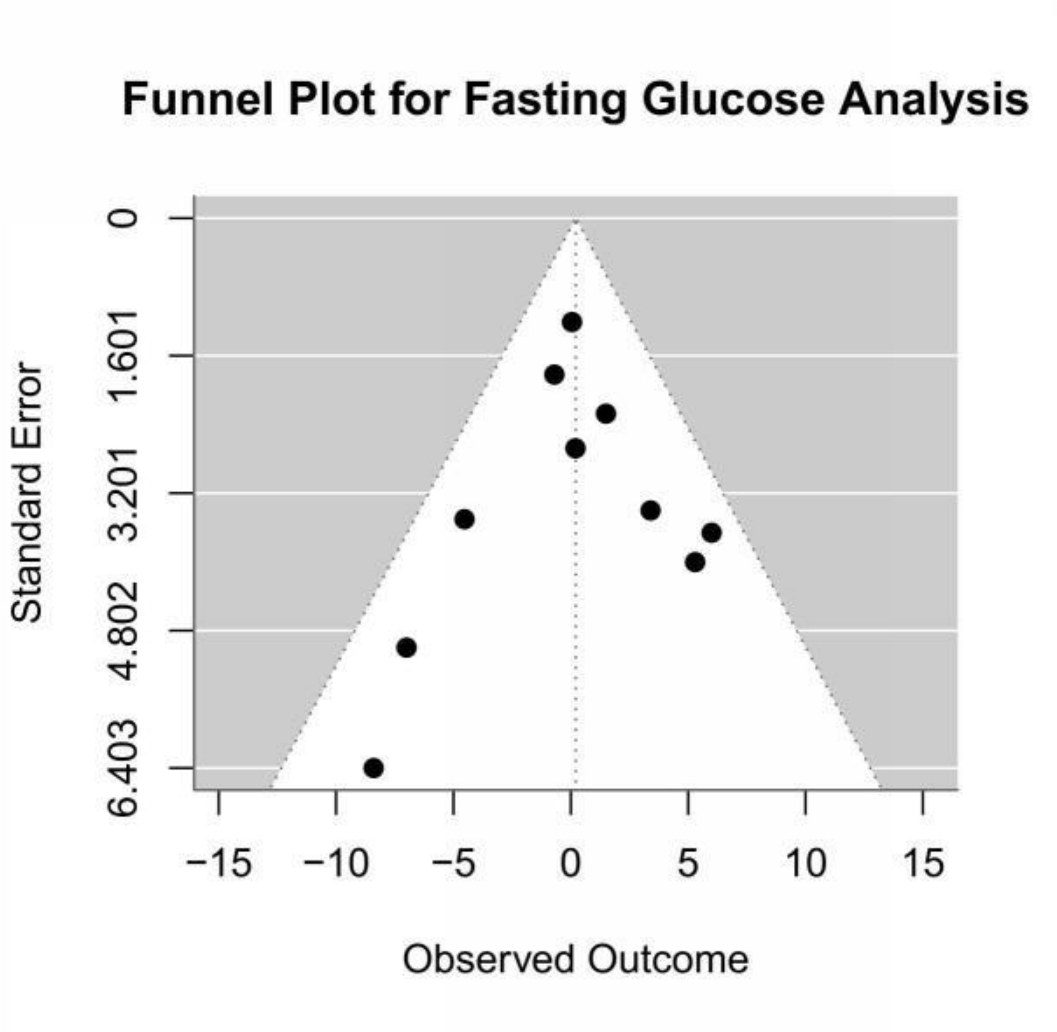

Figure S15. Funnel plot of fasting insulin for people aged 30–44 years.

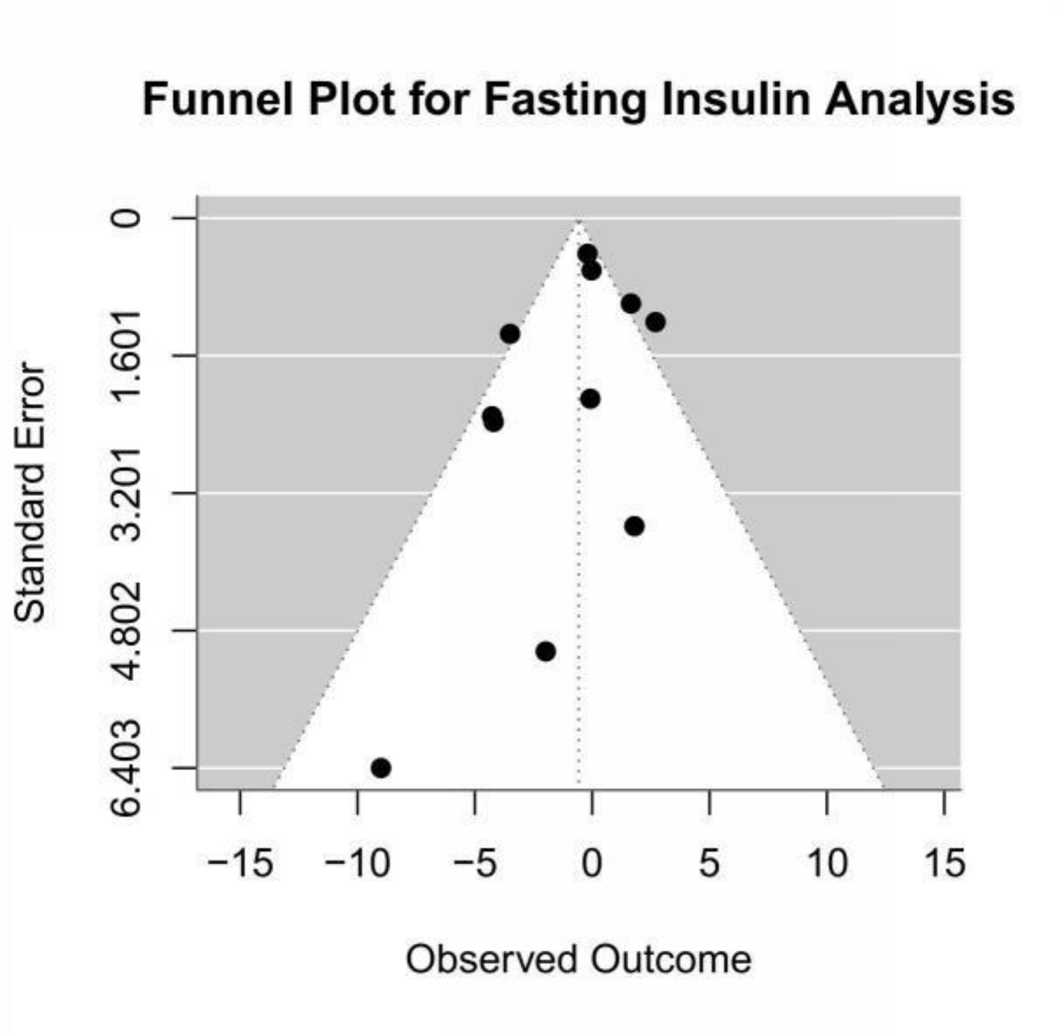

Figure S16. Bubble plot of LDL-C for people aged < 30 years.

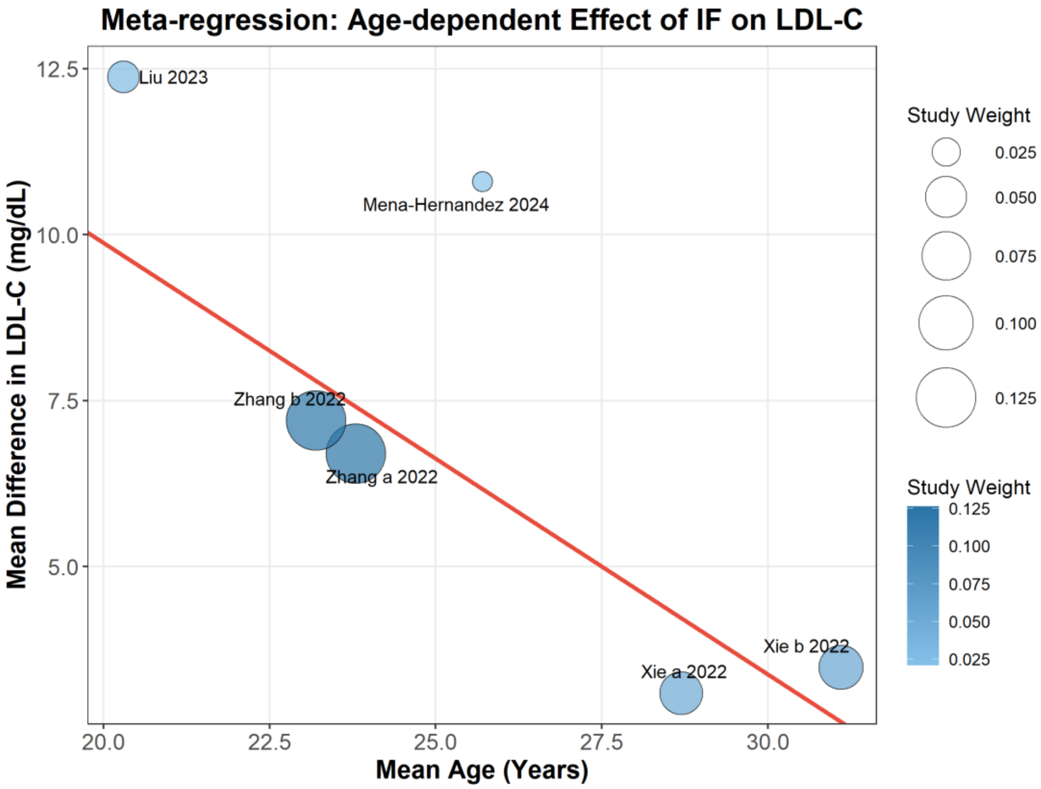

Figure S17. Forest plot comparing dropout rates between intermittent fasting (IF) and control groups stratified by age.

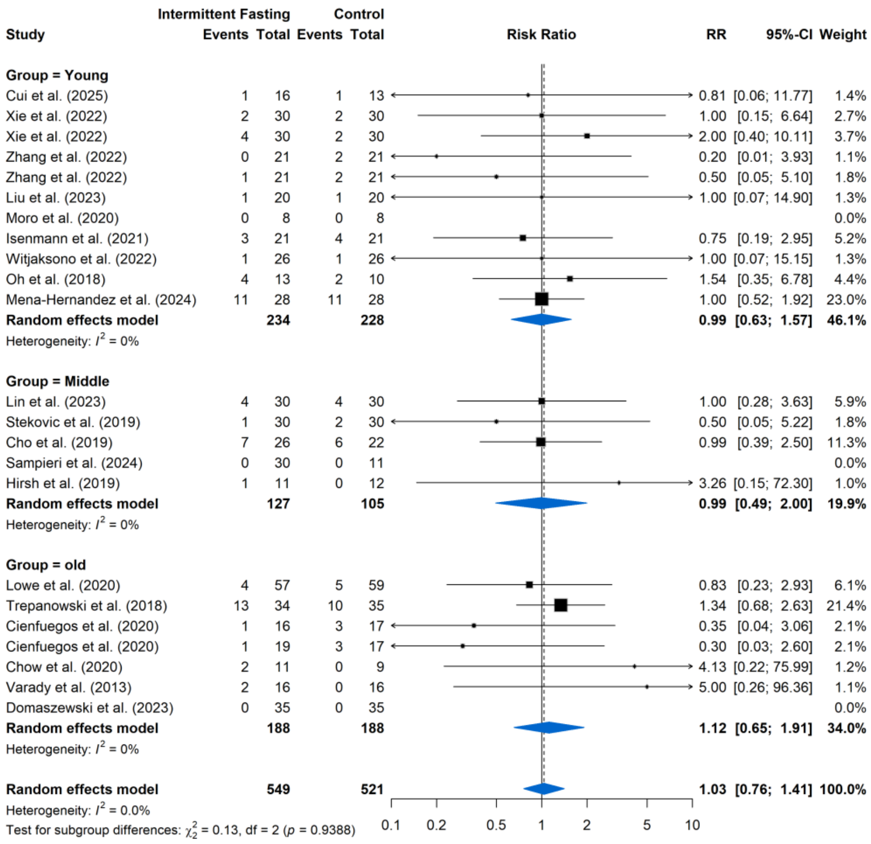

Table S4. Risk of bias assessment table.

reference, year	Randomisation process	Deviations from intended interventions	Missing outcome data	Measurements of the outcome	Selection of the reported results	Overall bias
Cui et al., 2025	Some concerns	Some concerns	Some concerns	Some concerns	Low	Some concerns
Oldenburg et al., 2025	Low	Some concerns	Some concerns	Some concerns	Low	Some concerns
Rizvi et al., 2024	Some concerns	Some concerns	Some concerns	Some concerns	Low	Some concerns
Sampieril et al., 2024	Some concerns	Some concerns	Some concerns	Some concerns	Low	Some concerns
Akasheh et al., 2024	Some concerns	Some concerns	Some concerns	Some concerns	Some concerns	Some concerns
Lin et al., 2023	Some concerns	Some concerns	Low	Some concerns	Low	Some concerns
Xie et al., 2022	Some concerns	Low	Low	Some concerns	Low	Some concerns
Isenmann et al., 2021	Some concerns	Some concerns	Low	Some concerns	Low	Some concerns
Lowe et al.,2020	Some concerns	Some concerns	Some concerns	Some concerns	Low	Some concerns
Moro et al., 2020	Some concerns	Low	Low	Low	Low	Some concerns
Stekovic et al., 2019	Some concerns	Low	Low	Low	Low	Some concerns
Cho et al., 2019	Some concerns	Some concerns	Some concerns	Low	Low	Some concerns
Trepanowski et al., 2019	Some concerns	Some concerns	Low	Some concerns	Low	Some concerns
Schübel et al., 2018	Some concerns	Some concerns	Low	Low	Low	Some concerns
Betts et al., 2014	Low	Some concerns	Low	Low	Low	Some concerns
Chow et al., 2020	Some concerns	Some concerns	Low	Low	Low	Some concerns

Hirsh et al., 2019	Some concerns	Low	Low	Low	Low	Some concerns
Stote et al., 2007	Some concerns	Some concerns	Some concerns	Some concerns	Low	Some concerns
Varady et al., 2013	Some concerns	Some concerns	Low	Low	Low	Some concerns
Domaszewski et al., 2022	Some concerns	Some concerns	Low	Some concerns	Low	Some concerns
Oh et al., 2017	Some concerns	Some concerns	Low	Some concerns	Low	Some concerns
Liu et al.,2023	Some concerns	Some concerns	Low	Low	Low	Some concerns
Witjaksono et al., 2022	Some concerns	Some concerns	Low	Some concerns	Low	Some concerns
Manoogian et al., 2022	Some concerns	Some concerns	Low	Low	Low	Some concerns
Mena-Hernández et al., 2024	Some concerns	Some concerns	Low	Some concerns	Low	Some concerns
Zhang et al., 2022	Some concerns	Some concerns	Low	Some concerns	Low	Some concerns
Cienfuegos et al.,2020	Some concerns	Some concerns	Low	Low	Low	Some concerns
Gabel et al.,2019	Some concerns	Some concerns	Some concerns	Low	Low	Some concerns

Figure S18. Subgroup analysis of intermittent fasting on body weight by intervention modality. The forest plot illustrates the pooled mean differences in body weight for Time-Restricted Feeding (TRF) and Intermittent Energy Restriction (IER, including ADF and 5:2 diet). The squares represent the mean difference (MD) for individual study comparisons, and the horizontal lines represent the 95% confidence intervals (CIs). The diamonds represent the pooled MD for each subgroup and the overall population. The test for subgroup differences ($P_{interaction}$ = 0.29) indicates no significant difference in weight loss efficacy between the TRF and IER protocols.

	Experimental			Control				Mean Difference
Study or Subgroup	Mean	SD	Total	Mean	SD	Total	Weight	IV, Random, 95% CI
**1.1.1 TRF**								
Akasheh 2024	-3.6	1.77	35	0.2	1.87	14	5.3%	-3.80 [-4.94, -2.66]
Betts 2014	-0.4	0.74	17	-0.2	1.22	16	6.1%	-0.20 [-0.89, 0.49]
Chow 2020	-3.6	13.98	11	-1.5	17.77	9	0.2%	-2.10 [-16.35, 12.15]
Cui 2025	-2.6	1.6	15	0.3	1.3	12	5.4%	-2.90 [-3.99, -1.81]
Domaszewski 2022a	-1.3	6.24	29	-0.5	5.76	28	2.3%	-0.80 [-3.92, 2.32]
Domaszewski 2022b	-1.8	6.01	25	0.7	5.19	26	2.3%	-2.50 [-5.59, 0.59]
Isenmann 2021	-3.8	2.1	18	-4	2.3	17	4.7%	0.20 [-1.26, 1.66]
Lin 2023	-3.49	9.69	77	1.12	8.13	77	2.6%	-4.61 [-7.44, -1.78]
Liu 2023	-3.03	2.89	19	-0.74	3.6	19	3.6%	-2.29 [-4.37, -0.21]
Lowe 2020	-1.7	2.07	22	-0.57	2.075	24	5.2%	-1.13 [-2.33, 0.07]
Manoogian 2022	-0.94	2.16	66	-0.43	2.18	70	6.1%	-0.51 [-1.24, 0.22]
Mena-Hernández 2024	-0.6	12.36	17	-0.1	12.27	17	0.5%	-0.50 [-8.78, 7.78]
Moro 2020	-1.26	0.71	8	0.22	1.05	8	5.8%	-1.48 [-2.36, -0.60]
Oldenburg 2025	-3	4.89	30	-1.6	5.36	29	2.8%	-1.40 [-4.02, 1.22]
Sampieril 2024a	-2.25	2.18	8	-0.43	0.68	3	4.3%	-1.82 [-3.52, -0.12]
Sampieril 2024b	-0.59	1.69	5	-0.43	0.68	3	4.3%	-0.16 [-1.83, 1.51]
Sampieril 2024c	-0.49	1.61	9	-0.43	0.68	4	5.1%	-0.06 [-1.31, 1.19]
Stote 2007	-0.6	7.72	15	0.8	7.72	15	0.9%	-1.40 [-6.93, 4.13]
Xie 2022a	-1.6	1.4	30	0.3	1.2	15	6.0%	-1.90 [-2.69, -1.11]
Xie 2022b	-0.2	2.2	30	0.3	1.2	15	5.6%	-0.50 [-1.49, 0.49]
Zhang 2022a	-3.5	1.52	21	-0.2	1.56	9	5.2%	-3.30 [-4.51, -2.09]
Zhang 2022b	-2.9	1.48	20	-0.2	1.56	10	5.3%	-2.70 [-3.86, -1.54]
**Subtotal (95% CI)**			**527**			**440**	**89.7%**	**-1.58 [-2.16, -0.99]**
Heterogeneity: Tau² = 1.15; Chi² = 78.36, df = 21 (P < 0.00001); I² = 73%								
Test for overall effect: Z = 5.28 (P < 0.00001)								
**1.1.2 ADF / 5: 2diet**								
Cho 2019	-3.9	1.97	8	-0.2	2.01	5	3.4%	-3.70 [-5.93, -1.47]
Gabel 2019	-8	6.6	11	0	3.87	15	1.4%	-8.00 [-12.36, -3.64]
Oh 2017	-2.4	3.1	13	-0.6	1.3	10	4.0%	-1.80 [-3.67, 0.07]
Witjaksono 2022	-1.27	5.73	25	-1.91	8.4	25	1.6%	0.64 [-3.35, 4.63]
**Subtotal (95% CI)**			**57**			**55**	**10.3%**	**-3.02 [-5.63, -0.40]**
Heterogeneity: Tau² = 4.70; Chi² = 10.03, df = 3 (P = 0.02); I² = 70%								
Test for overall effect: Z = 2.26 (P = 0.02)								
**Total (95% CI)**			**584**			**495**	**100.0%**	**-1.72 [-2.29, -1.14]**
Heterogeneity: Tau² = 1.29; Chi² = 92.36, df = 25 (P < 0.00001); I² = 73%								
Test for overall effect: Z = 5.82 (P < 0.00001)								
Test for subgroup differences: Chi² = 1.11, df = 1 (P = 0.29), I² = 9.6%								

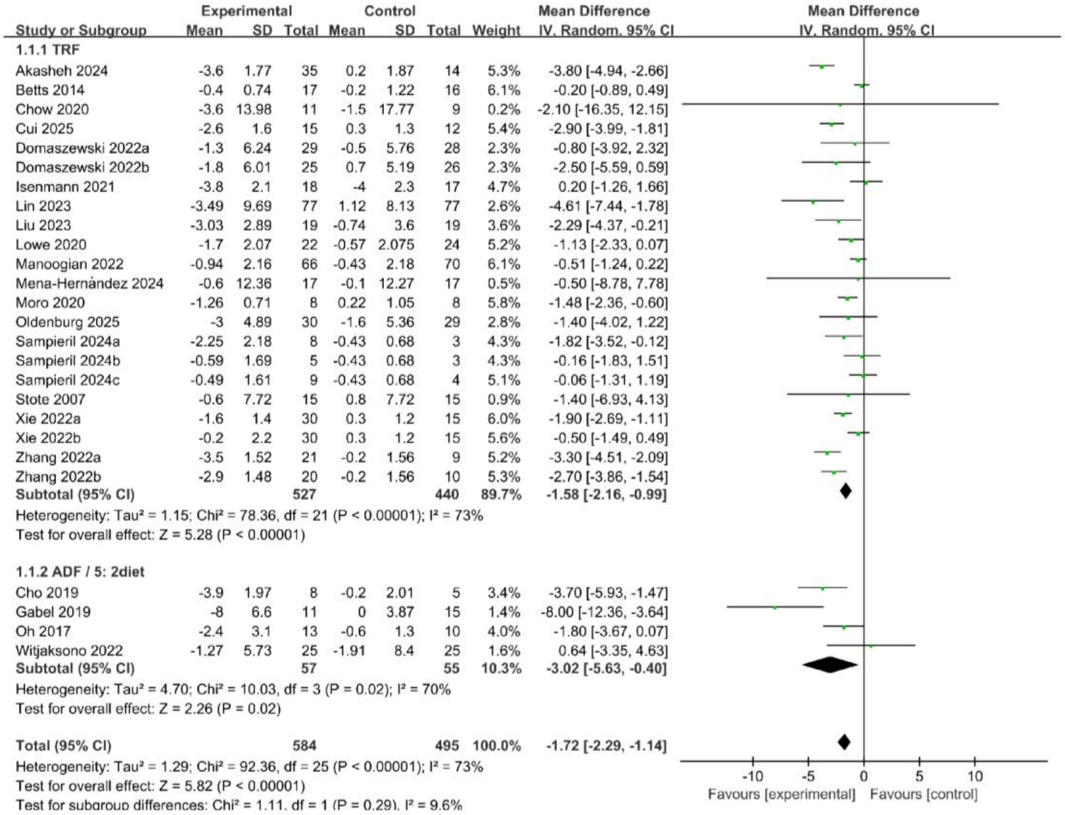

Figure S19. Subgroup analysis of intermittent fasting on fat mass (FM) by intervention modality. The forest plot displays the reduction in fat mass across TRF and IER subgroups. While both strategies effectively reduced adiposity, the IER subgroup showed a numerically larger point estimate. However, the test for subgroup differences ($P_{interaction}$ = 0.06) confirms that there was no statistically significant difference in fat-loss efficacy between the two fasting modalities.

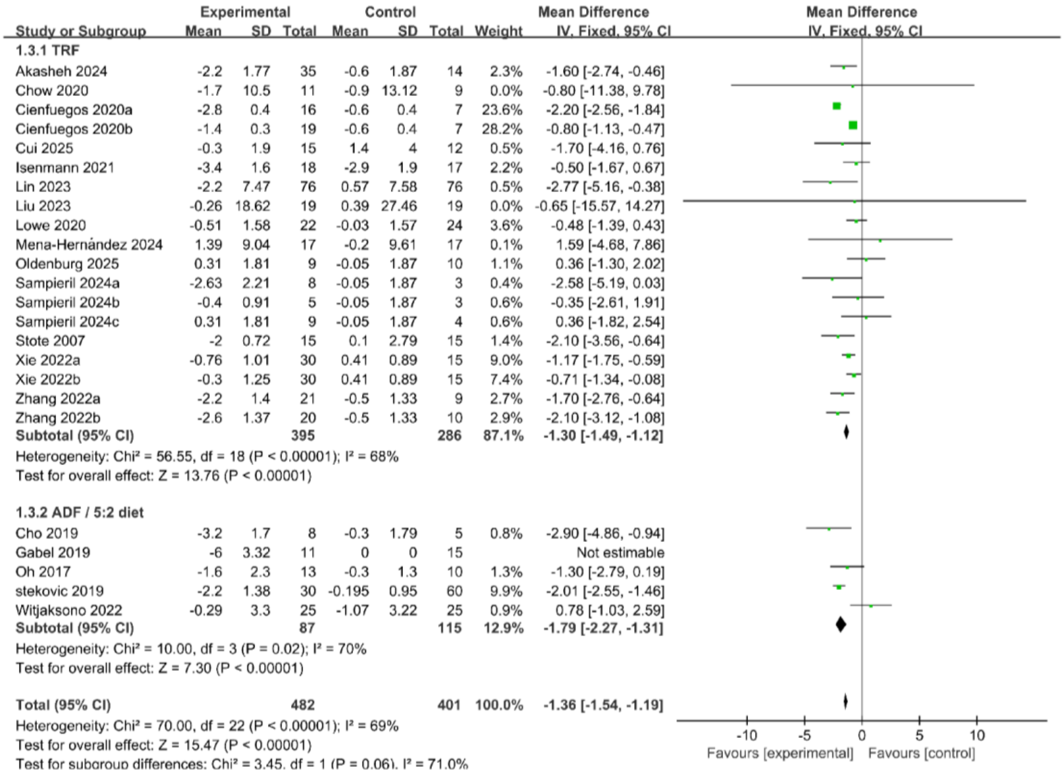

Figure S20. Subgroup analysis of intermittent fasting on fat-free mass (FFM) by intervention modality. This analysis evaluates the impact of TRF and IER on lean mass preservation. Significant reductions in FFM were observed in both subgroups. The test for subgroup differences ($P_{interaction}$ = 0.08) indicates that the risk of muscle mass depletion is comparable between TRF and IER, reinforcing the need for adjunctive muscle-protective strategies across all intermittent fasting types.

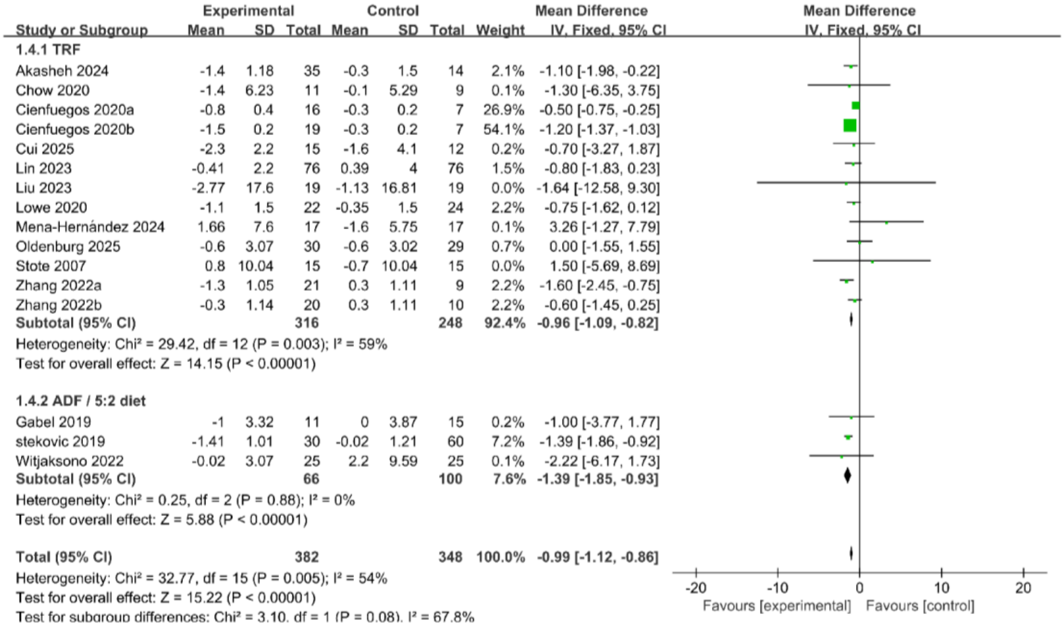

	Experimental			Control				Mean Difference
Study or Subgroup	Mean	SD	Total	Mean	SD	Total	Weight	IV, Fixed, 95% CI
**1.4.1 TRF**								
Akasheh 2024	-1.4	1.18	35	-0.3	1.5	14	2.1%	-1.10 [-1.98, -0.22]
Chow 2020	-1.4	6.23	11	-0.1	5.29	9	0.1%	-1.30 [-6.35, 3.75]
Cienfuegos 2020a	-0.8	0.4	16	-0.3	0.2	7	26.9%	-0.50 [-0.75, -0.25]
Cienfuegos 2020b	-1.5	0.2	19	-0.3	0.2	7	54.1%	-1.20 [-1.37, -1.03]
Cui 2025	-2.3	2.2	15	-1.6	4.1	12	0.2%	-0.70 [-3.27, 1.87]
Lin 2023	-0.41	2.2	76	0.39	4	76	1.5%	-0.80 [-1.83, 0.23]
Liu 2023	-2.77	17.6	19	-1.13	16.81	19	0.0%	-1.64 [-12.58, 9.30]
Lowe 2020	-1.1	1.5	22	-0.35	1.5	24	2.2%	-0.75 [-1.62, 0.12]
Mena-Hernández 2024	1.66	7.6	17	-1.6	5.75	17	0.1%	3.26 [-1.27, 7.79]
Oldenburg 2025	-0.6	3.07	30	-0.6	3.02	29	0.7%	0.00 [-1.55, 1.55]
Stote 2007	0.8	10.04	15	-0.7	10.04	15	0.0%	1.50 [-5.69, 8.69]
Zhang 2022a	-1.3	1.05	21	0.3	1.11	9	2.2%	-1.60 [-2.45, -0.75]
Zhang 2022b	-0.3	1.14	20	0.3	1.11	10	2.2%	-0.60 [-1.45, 0.25]
**Subtotal (95% CI)**			**316**			**248**	**92.4%**	**-0.96 [-1.09, -0.82]**
Heterogeneity: Chi² = 29.42, df = 12 (P = 0.003); I² = 59%								
Test for overall effect: Z = 14.15 (P < 0.00001)								
**1.4.2 ADF / 5:2 diet**								
Gabel 2019	-1	3.32	11	0	3.87	15	0.2%	-1.00 [-3.77, 1.77]
stekovic 2019	-1.41	1.01	30	-0.02	1.21	60	7.2%	-1.39 [-1.86, -0.92]
Witjaksono 2022	-0.02	3.07	25	2.2	9.59	25	0.1%	-2.22 [-6.17, 1.73]
**Subtotal (95% CI)**			**66**			**100**	**7.6%**	**-1.39 [-1.85, -0.93]**
Heterogeneity: Chi² = 0.25, df = 2 (P = 0.88); I² = 0%								
Test for overall effect: Z = 5.88 (P < 0.00001)								
**Total (95% CI)**			**382**			**348**	**100.0%**	**-0.99 [-1.12, -0.86]**
Heterogeneity: Chi² = 32.77, df = 15 (P = 0.005); I² = 54%								
Test for overall effect: Z = 15.22 (P < 0.00001)								
Test for subgroup differences: Chi² = 3.10, df = 1 (P = 0.08), I² = 67.8%								

Figure S21. Subgroup analysis of intermittent fasting on low-density lipoprotein cholesterol (LDL-C) by intervention modality. This plot compares the impact of TRF and IER on LDL-C levels. Both modalities were associated with significant elevations in LDL-C, as indicated by the pooled estimates. The statistical test for subgroup differences ($P_{interaction}$ = 0.28) suggests that the lipid-elevating effect is a generalized response to fasting interventions regardless of the specific dietary protocol employed.

	Experimental			Control				Mean Difference
Study or Subgroup	Mean	SD	Total	Mean	SD	Total	Weight	IV, Fixed, 95% CI
**1.2.1 TRF**								
Betts 2014	3.9	20.3	17	0.4	23.67	16	0.3%	3.50 [-11.59, 18.59]
Chow 2020	9	18.62	11	1	12.02	9	0.4%	8.00 [-5.52, 21.52]
Cienfuegos 2020a	2.6	5.7	16	-2	3.7	7	4.8%	4.60 [0.69, 8.51]
Cienfuegos 2020b	-4.8	5.1	19	-2	3.7	7	5.7%	-2.80 [-6.37, 0.77]
Lin 2023	-0.86	36.34	74	2.37	30.17	74	0.6%	-3.23 [-13.99, 7.53]
Liu 2023	17.79	17.32	19	5.41	18.5	19	0.6%	12.38 [0.98, 23.78]
Manoogian 2022	0.75	18.2	69	-2.61	19.54	66	1.8%	3.36 [-3.02, 9.74]
Mena-Hernández 2024	4.7	18.65	15	-6.1	19.32	15	0.4%	10.80 [-2.79, 24.39]
Oldenburg 2025	-0.1	17.72	29	-4.2	17.56	26	0.8%	4.10 [-5.24, 13.44]
Rizvi 2024	-9.9	16.53	30	10.5	30.2	30	0.5%	-20.40 [-32.72, -8.08]
Sampieril 2024a	9.2	28.19	8	-1.1	4.7	3	0.2%	10.30 [-9.95, 30.55]
Sampieril 2024b	-2.1	16.57	5	-1.1	4.7	3	0.3%	-1.00 [-16.47, 14.47]
Sampieril 2024c	-4.9	13.02	9	-1.1	4.7	4	0.8%	-3.80 [-13.47, 5.87]
Stote 2007	27.1	22.89	15	4.2	22.89	15	0.3%	22.90 [6.52, 39.28]
Xie 2022a	0	18.95	30	-3.09	13.92	15	0.8%	3.09 [-6.69, 12.87]
Xie 2022b	0.39	17.79	30	-3.09	13.92	15	0.8%	3.48 [-6.01, 12.97]
Zhang 2022a	19.2	8.88	21	12.5	8.9	9	1.5%	6.70 [-0.25, 13.65]
Zhang 2022b	19.7	8.78	20	12.5	8.9	10	1.6%	7.20 [0.47, 13.93]
**Subtotal (95% CI)**			**437**			**343**	**22.2%**	**2.30 [0.48, 4.11]**
Heterogeneity: Chi² = 40.71, df = 17 (P = 0.001); I² = 58%								
Test for overall effect: Z = 2.48 (P = 0.01)								
**1.2.2 ADF / 5: 2diet**								
Cho 2019	0	28	8	16.9	28.17	5	0.1%	-16.90 [-48.30, 14.50]
Gabel 2019	7	13.27	11	0	15.49	15	0.6%	7.00 [-4.09, 18.09]
Hirsh 2019	5.2	18.3	10	9.3	14.1	12	0.4%	-4.10 [-17.97, 9.77]
Schübel 2018	-7.5	2.6	49	-8.7	2.4	52	76.4%	1.20 [0.22, 2.18]
Varady 2013	-18	23.24	15	-9	15.49	15	0.4%	-9.00 [-23.13, 5.13]
**Subtotal (95% CI)**			**93**			**99**	**77.8%**	**1.15 [0.18, 2.12]**
Heterogeneity: Chi² = 4.88, df = 4 (P = 0.30); I² = 18%								
Test for overall effect: Z = 2.33 (P = 0.02)								
**Total (95% CI)**			**530**			**442**	**100.0%**	**1.41 [0.55, 2.26]**
Heterogeneity: Chi² = 46.78, df = 22 (P = 0.002); I² = 53%								
Test for overall effect: Z = 3.23 (P = 0.001)								
Test for subgroup differences: Chi² = 1.18, df = 1 (P = 0.28), I² = 15.5%								

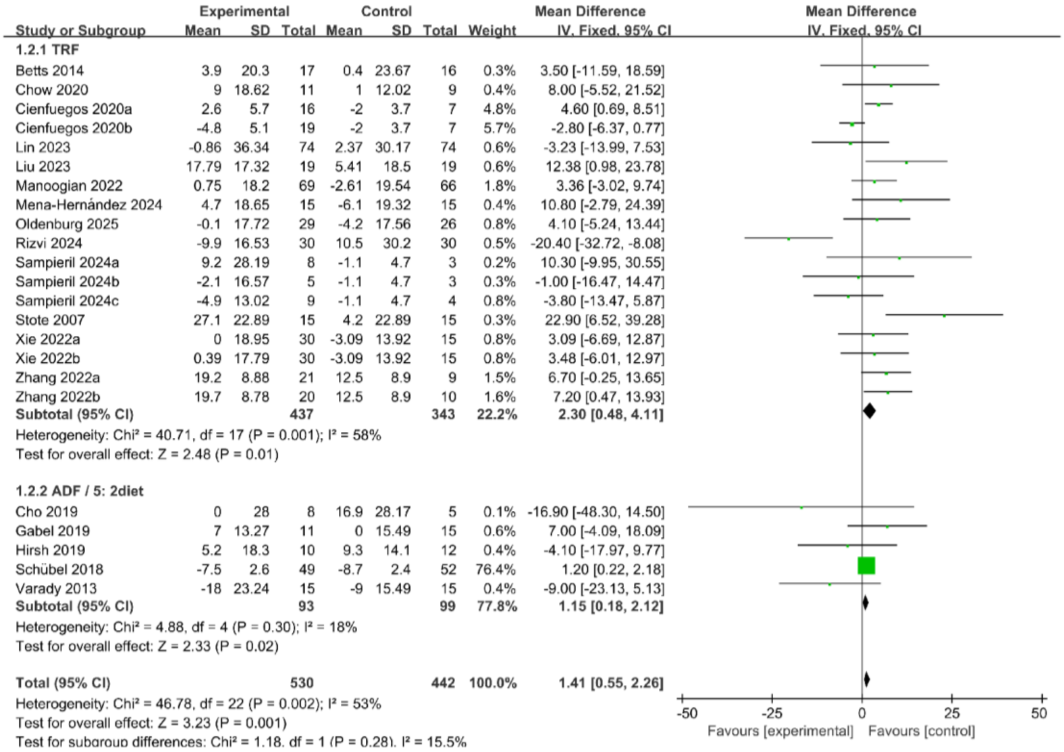

Table S5: Methodological sensitivity analysis comparing standard versus conservative correlation coefficient assumptions.

Outcome	Age Group	Original Pooled MD (r=0.8)	Sensitivity Pooled MD (r=0.5)	Conclusion Changed?
Body Weight	<30y	–1.80 [–2.57, –1.03] P<0.001	–1.88 [–2.28, –1.47] P<0.001	No
	30–44y	–1.47 [–2.44, –0.49] P=0.003	–0.69 [–1.11, –0.28] P=0.001	No
	≥45y	–2.16 [–3.67, –0.65] P=0.005	–2.47 [–3.27, –1.67] P<0.001	No
FM	<30y	–1.09 [–1.54, –0.64] P<0.001	–1.08 [–1.42, –0.75] P<0.001	No
	30–44y	–1.22 [–2.55, 0.10] P=0.07	–1.09 [–1.95, –0.23] P=0.01	Yes
	≥45y	–1.49 [–2.19, –0.79] P<0.001	–1.48 [–1.69, –1.27] P<0.001	No
FFM	<30y	–0.98 [–1.80, –0.16] P=0.02	–1.03 [–1.61, –0.46] P<0.001	No
	30–44y	–0.60 [–1.41, 0.22] P=0.15	–0.60 [–1.41, 0.22] P=0.15	No
	≥45y	–0.98 [–1.39, –0.57] P<0.001	–1.00 [–1.13, –0.87] P<0.001	No
LDL-C	<30y	6.75 [3.15, 10.34] P<0.001	6.22 [2.45, 10.00] P=0.001	No
	30–44y	1.44 [–2.08, 4.96] P=0.42	1.44 [–2.08, 4.96] P=0.42	No
	≥45y	0.19 [–4.32, 4.70] P=0.93	1.09 [0.18, 2.00] P=0.02	Yes

Figure S22. Age-stratified forest plot of the effect of intermittent fasting on body weight. The forest plot illustrates the mean differences (MD) and 95% confidence intervals (CIs) comparing intermittent fasting interventions with control diets across three distinct age cohorts: young (<30 years), early middle-aged (30–44 years), and middle-aged and older (≥45 years) adults. The squares represent the point estimate of each individual trial, with the size of the square proportional to the study's assigned weight in the random-effects meta-analysis. The horizontal lines denote the 95% CIs. The diamonds represent the pooled effect estimates for each age subgroup and the overall analysis. The solid vertical line indicates the line of no effect (MD = 0).

Study or Subgroup	IF Group Mean	SD	Total	Control Mean	SD	Total	Weight (common)	Weight (random)	Mean Difference IV, Fixed + Random, 95% CI
**Young adults (<30 years)**									
Cui 2025	-2.60	1.6000	15	0.30	1.3000	12	6.2%	5.6%	-2.90 [-3.99; -1.81]
Isenmann 2021	-3.80	2.1000	18	-4.00	2.3000	17	3.4%	4.9%	0.20 [-1.26; 1.66]
Liu 2023	-3.03	2.8900	19	-0.74	3.6000	19	1.7%	3.8%	-2.29 [-4.37; -0.21]
Mena-Hernandez 2024	-0.60	12.3600	17	-0.10	12.2700	17	0.1%	0.5%	-0.50 [-8.78; 7.78]
Moro 2020	-1.26	3.1500	8	0.22	4.0200	8	0.6%	2.1%	-1.48 [-5.02; 2.06]
Oh 2017	-2.40	3.1000	13	-0.60	1.3000	10	2.1%	4.1%	-1.80 [-3.67; 0.07]
Witjaksono 2022	-1.27	5.7300	25	-1.91	8.4000	25	0.5%	1.7%	0.64 [-3.35; 4.63]
Xie 2022a	-1.60	1.4000	30	0.30	1.2000	15	11.9%	6.1%	-1.90 [-2.69; -1.11]
Xie 2022b	-0.20	2.2000	30	0.30	1.2000	15	7.5%	5.8%	-0.50 [-1.49; 0.49]
Zhang 2022a	-3.50	1.5200	21	-0.20	1.5600	9	5.0%	5.4%	-3.30 [-4.51; -2.09]
Zhang 2022b	-2.90	1.4800	20	-0.20	1.5600	10	5.4%	5.5%	-2.70 [-3.86; -1.54]
**Total (common effect, 95% CI)**			**216**			**157**	**44.4%**	.	**-1.87 [-2.28; -1.46]**
**Total (random effect, 95% CI)**							.	**45.4%**	**-1.80 [-2.57; -1.03]**
Heterogeneity: $Tau^2 = 0.9005$; $Chi^2 = 27.57$, df = 10 (P = 0.0021); $I^2 = 63.7\%$									
Test for overall effect (common effect): Z = -9.01 (P < 0.0001)									
Test for overall effect (random effects): Z = -4.57 (P < 0.0001)									
**Early middle-aged adults (30-44 years)**									
Betts 2014	-0.40	0.7400	17	-0.20	1.2200	16	15.3%	6.3%	-0.20 [-0.89; 0.49]
Cho 2019	-3.90	1.9800	8	-0.20	2.0100	5	1.5%	3.6%	-3.70 [-5.93; -1.47]
Gabel 2019	-8.00	6.6300	11	0.00	3.8700	15	0.4%	1.5%	-8.00 [-12.38; -3.62]
Lin 2023	-3.49	9.6900	77	1.12	8.1300	77	0.9%	2.8%	-4.61 [-7.44; -1.78]
Manoogian 2022	-0.94	2.1600	66	-0.43	2.1800	70	13.8%	6.2%	-0.51 [-1.24; 0.22]
Oldenburg 2025	-3.00	4.8900	30	-1.60	5.3600	29	1.1%	3.0%	-1.40 [-4.02; 1.22]
Sampieril 2024a	-2.25	2.1800	8	-0.43	0.6800	3	2.6%	4.5%	-1.82 [-3.52; -0.12]
Sampieril 2024b	-0.59	1.6900	5	-0.43	0.6800	3	2.6%	4.5%	-0.16 [-1.83; 1.51]
Sampieril 2024c	-0.49	1.6100	9	-0.43	0.6800	4	4.8%	5.3%	-0.06 [-1.31; 1.19]
**Total (common effect, 95% CI)**			**231**			**222**	**43.0%**	.	**-0.69 [-1.11; -0.28]**
**Total (random effect, 95% CI)**							.	**37.6%**	**-1.47 [-2.44; -0.49]**
Heterogeneity: $Tau^2 = 1.3488$; $Chi^2 = 30.58$, df = 8 (P = 0.0002); $I^2 = 73.8\%$									
Test for overall effect (common effect): Z = -3.28 (P = 0.0010)									
Test for overall effect (random effects): Z = -2.94 (P = 0.0032)									
**Middle-aged and older adults (≥45 years)**									
Akasheh 2024	-3.60	1.7700	35	0.20	1.8700	14	5.7%	5.5%	-3.80 [-4.94; -2.66]
Chow 2020	-3.60	13.9800	11	-1.50	17.7700	9	0.0%	0.2%	-2.10 [-16.35; 12.15]
Domaszewski 2022a	-1.30	6.2400	29	-0.50	5.7600	28	0.8%	2.4%	-0.80 [-3.92; 2.32]
Domaszewski 2022b	-1.80	6.0100	25	0.70	5.1900	26	0.8%	2.5%	-2.50 [-5.59; 0.59]
Lowe 2020	-1.70	2.0700	22	-0.57	2.0750	24	5.1%	5.4%	-1.13 [-2.33; 0.07]
Stote 2007	-0.60	7.7200	15	0.80	7.7200	15	0.2%	1.0%	-1.40 [-6.93; 4.13]
**Total (common effect, 95% CI)**			**137**			**116**	**12.6%**	.	**-2.40 [-3.17; -1.64]**
**Total (random effect, 95% CI)**							.	**17.0%**	**-2.16 [-3.67; -0.65]**
Heterogeneity: $Tau^2 = 1.5197$; $Chi^2 = 11.23$, df = 5 (P = 0.0470); $I^2 = 55.5\%$									
Test for overall effect (common effect): Z = -6.15 (P < 0.0001)									
Test for overall effect (random effects): Z = -2.81 (P = 0.0050)									
**Total (common effect, 95% CI)**			**584**			**495**	**100.0%**	.	**-1.43 [-1.70; -1.16]**
**Total (random effect, 95% CI)**							.	**100.0%**	**-1.73 [-2.34; -1.12]**
Heterogeneity: $Tau^2 = 1.4084$; $Chi^2 = 92.27$, df = 25 (P < 0.0001); $I^2 = 72.9\%$									
Test for subgroup differences (common effect): $Chi^2 = 22.88$, df = 2 (P < 0.0001)									
Test for subgroup differences (random effects): $Chi^2 = 0.63$, df = 2 (P = 0.7314)									

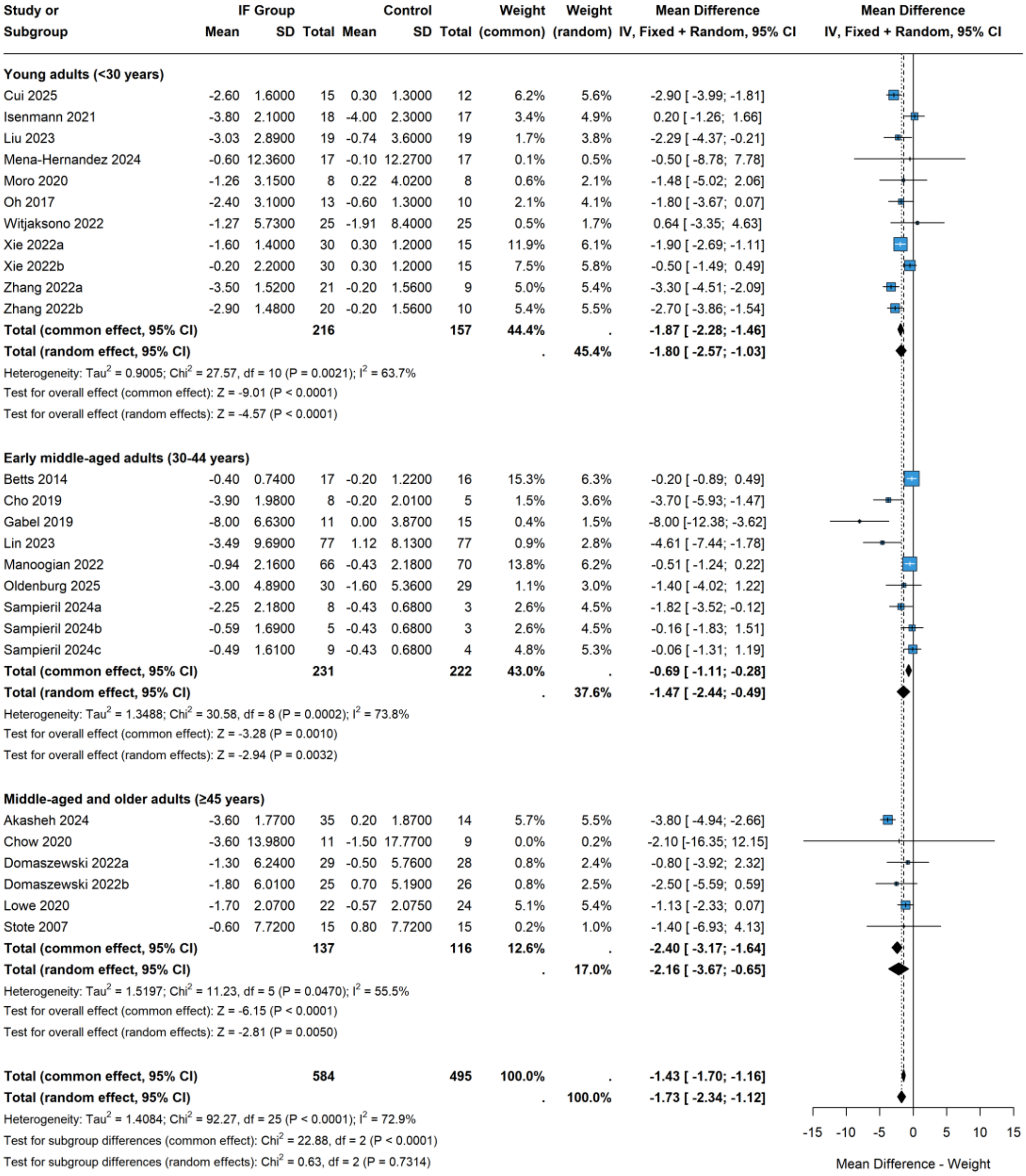

Figure S23. Age-stratified forest plot of the effect of intermittent fasting on body mass index (BMI).The forest plot illustrates the mean differences (MD) and 95% confidence intervals (CIs) comparing intermittent fasting interventions with control diets across three distinct age cohorts: young (<30 years), early middle-aged (30–44 years), and middle-aged and older (≥45 years) adults. The squares represent the point estimate of each individual trial, with the size of the square proportional to the study's assigned weight in the random-effects meta-analysis. The horizontal lines denote the 95% CIs. The diamonds represent the pooled effect estimates for each age subgroup and the overall analysis. The solid vertical line indicates the line of no effect (MD = 0).

Study or	IF Group			Control			Weight	Weight	Mean Difference
**Subgroup**	**Mean**	**SD**	**Total**	**Mean**	**SD**	**Total**	**(common)**	**(random)**	**IV, Fixed + Random, 95% CI**
**Young adults (<30 years)**									
Cui 2025	-1.00	0.6000	15	0.10	0.5000	12	6.4%	7.6%	-1.10 [-1.52; -0.68]
Isenmann 2021	-1.30	0.7000	18	-1.30	0.7000	17	5.1%	7.3%	0.00 [-0.46; 0.46]
Liu 2023	-1.58	0.7800	19	-0.58	0.7400	19	4.7%	7.2%	-1.00 [-1.48; -0.52]
Mena-Hernandez 2024	-0.20	4.0200	17	0.00	3.9500	17	0.2%	1.0%	-0.20 [-2.88; 2.48]
Oh 2017	-0.90	1.3000	13	-0.10	0.5000	10	1.8%	5.4%	-0.80 [-1.57; -0.03]
Witjaksono 2022	-0.55	2.1200	25	-0.64	2.9000	25	0.6%	2.7%	0.09 [-1.32; 1.50]
Zhang 2022a	-1.20	0.5800	21	-0.10	0.5600	9	5.6%	7.4%	-1.10 [-1.54; -0.66]
Zhang 2022b	-1.00	0.5700	20	-0.10	0.5600	10	6.0%	7.5%	-0.90 [-1.33; -0.47]
**Total (common effect, 95% CI)**			**148**			**119**	**30.3%**	**.**	**-0.82 [-1.01; -0.63]**
**Total (random effect, 95% CI)**							**.**	**46.1%**	**-0.77 [-1.11; -0.44]**
Heterogeneity: $Tau^2 = 0.1227$; $Chi^2 = 17.76$, df = 7 (P = 0.0131); $I^2 = 60.6\%$									
Test for overall effect (common effect): Z = -8.40 (P < 0.0001)									
Test for overall effect (random effects): Z = -4.52 (P < 0.0001)									
**Early middle-aged adults (30-44 years)**									
Betts 2014	-0.15	0.2600	17	-0.04	0.4100	16	19.7%	8.6%	-0.11 [-0.35; 0.13]
Cho 2019	-1.40	0.8400	8	-0.10	0.8900	5	1.2%	4.3%	-1.30 [-2.27; -0.33]
Gabel 2019	-2.00	0.0000	11	0.00	0.0000	15	0.0%	0.0%	-2.00
Lin 2023	-1.29	3.5400	76	0.40	3.0500	76	1.0%	4.0%	-1.69 [-2.74; -0.64]
Manoogian 2022	-0.26	0.8100	70	-0.09	0.8100	67	14.9%	8.4%	-0.17 [-0.44; 0.10]
Oldenburg 2025	-1.00	1.1000	29	-0.30	1.0400	26	3.4%	6.6%	-0.70 [-1.27; -0.13]
**Total (common effect, 95% CI)**			**211**			**205**	**40.1%**	**.**	**-0.26 [-0.42; -0.09]**
**Total (random effect, 95% CI)**							**.**	**31.9%**	**-0.55 [-0.96; -0.14]**
Heterogeneity: $Tau^2 = 0.1367$; $Chi^2 = 15.8$, df = 4 (P = 0.0033); $I^2 = 74.7\%$									
Test for overall effect (common effect): Z = -3.04 (P = 0.0024)									
Test for overall effect (random effects): Z = -2.61 (P = 0.0091)									
**Middle-aged and older adults (≥45 years)**									
Akasheh 2024	-1.30	1.1800	35	-0.20	1.1200	14	2.2%	5.8%	-1.10 [-1.80; -0.40]
Domaszewski 2022a	-1.40	7.2000	29	-0.30	2.3600	28	0.1%	0.9%	-1.10 [-3.86; 1.66]
Domaszewski 2022b	-0.50	1.1500	25	0.20	1.0800	26	2.9%	6.3%	-0.70 [-1.31; -0.09]
Rizvi 2024	-3.02	2.1400	30	-0.14	17.0900	30	0.0%	0.2%	-2.88 [-9.04; 3.28]
stekovic 2019	-1.21	0.5000	30	-0.03	0.4500	60	24.3%	8.7%	-1.18 [-1.39; -0.97]
**Total (common effect, 95% CI)**			**149**			**158**	**29.6%**	**.**	**-1.13 [-1.32; -0.94]**
**Total (random effect, 95% CI)**							**.**	**21.9%**	**-1.13 [-1.32; -0.94]**
Heterogeneity: $Tau^2 = 0$; $Chi^2 = 2.42$, df = 4 (P = 0.6587); $I^2 = 0\%$									
Test for overall effect (common effect): Z = -11.50 (P < 0.0001)									
Test for overall effect (random effects): Z = -11.50 (P < 0.0001)									
**Total (common effect, 95% CI)**			**508**			**482**	**100.0%**	**.**	**-0.68 [-0.79; -0.58]**
**Total (random effect, 95% CI)**							**.**	**100.0%**	**-0.77 [-1.05; -0.49]**
Heterogeneity: $Tau^2 = 0.2221$; $Chi^2 = 84.13$, df = 17 (P < 0.0001); $I^2 = 79.8\%$									
Test for subgroup differences (common effect): $Chi^2 = 48.14$, df = 2 (P < 0.0001)									
Test for subgroup differences (random effects): $Chi^2 = 7.94$, df = 2 (P = 0.0188)									

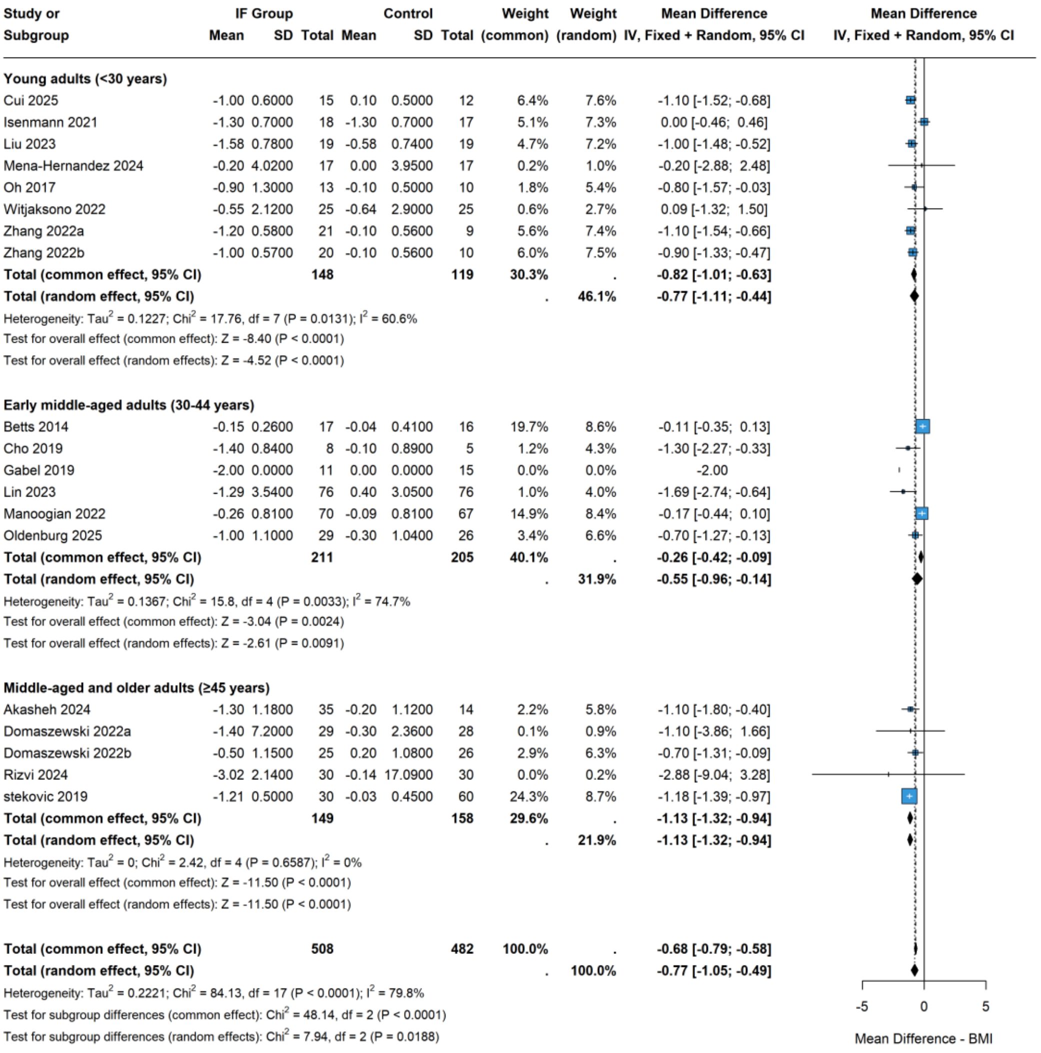

Figure S24. Age-stratified forest plot of the effect of intermittent fasting on fat mass (FM).The forest plot illustrates the mean differences (MD) and 95% confidence intervals (CIs) comparing intermittent fasting interventions with control diets across three distinct age cohorts: young (<30 years), early middle-aged (30–44 years), and middle-aged and older (≥45 years) adults. The squares represent the point estimate of each individual trial, with the size of the square proportional to the study's assigned weight in the random-effects meta-analysis. The horizontal lines denote the 95% CIs. The diamonds represent the pooled effect estimates for each age subgroup and the overall analysis. The solid vertical line indicates the line of no effect (MD = 0).

Study or	IF Group			Control			Weight	Weight	Mean Difference
Subgroup	Mean	SD	Total	Mean	SD	Total	(common)	(random)	IV, Fixed + Random, 95% CI
**Young adults (<30 years)**									
Cui 2025	-0.30	1.9000	15	1.40	4.0000	12	0.5%	2.1%	-1.70 [-4.16; 0.76]
Isenmann 2021	-3.40	1.6000	18	-2.90	1.9000	17	2.2%	5.4%	-0.50 [-1.67; 0.67]
Liu 2023	-0.26	18.6200	19	0.39	27.4600	19	0.0%	0.1%	-0.65 [-15.57; 14.27]
Mena-Hernandez 2024	1.39	9.0400	17	-0.20	9.6100	17	0.1%	0.4%	1.59 [-4.68; 7.86]
Oh 2017	-1.60	2.3000	13	-0.30	1.3000	10	1.4%	4.2%	-1.30 [-2.79; 0.19]
Witjaksono 2022	-0.29	3.3000	25	-1.07	3.2200	25	0.9%	3.3%	0.78 [-1.03; 2.59]
Xie 2022a	-0.76	1.0100	30	0.41	0.8900	15	9.0%	8.0%	-1.17 [-1.75; -0.59]
Xie 2022b	-0.30	1.2500	30	0.41	0.8900	15	7.5%	7.8%	-0.71 [-1.34; -0.08]
Zhang 2022a	-2.20	1.4000	21	-0.50	1.3300	9	2.7%	5.8%	-1.70 [-2.76; -0.64]
Zhang 2022b	-2.60	1.3700	20	-0.50	1.3300	10	2.9%	6.0%	-2.10 [-3.12; -1.08]
**Total (common effect, 95% CI)**			**208**			**149**	**27.1%**	.	**-1.08 [-1.42; -0.75]**
**Total (random effect, 95% CI)**							.	**43.1%**	**-1.09 [-1.54; -0.64]**
Heterogeneity: $Tau^2 = 0.1332$; $Chi^2 = 12.61$, df = 9 (P = 0.1810); $I^2 = 28.6\%$									
Test for overall effect (common effect): Z = -6.38 (P < 0.0001)									
Test for overall effect (random effects): Z = -4.72 (P < 0.0001)									
**Early middle-aged adults (30-44 years)**									
Cho 2019	-3.20	1.7000	8	-0.30	1.7900	5	0.8%	3.0%	-2.90 [-4.86; -0.94]
Gabel 2019	-6.00	3.3200	11	0.00	0.0000	15	0.0%	0.0%	-6.00
Lin 2023	-2.20	7.4700	76	0.57	7.5800	76	0.5%	2.2%	-2.77 [-5.16; -0.38]
Oldenburg 2025	0.31	1.8100	9	-0.05	1.8700	10	1.1%	3.7%	0.36 [-1.30; 2.02]
Sampieril 2024a	-2.63	2.2100	8	-0.05	1.8700	3	0.4%	1.9%	-2.58 [-5.19; 0.03]
Sampieril 2024b	-0.40	0.9100	5	-0.05	1.8700	3	0.6%	2.4%	-0.35 [-2.61; 1.91]
Sampieril 2024c	0.31	1.8100	9	-0.05	1.8700	4	0.6%	2.5%	0.36 [-1.82; 2.54]
**Total (common effect, 95% CI)**			**126**			**116**	**4.1%**	.	**-1.09 [-1.95; -0.23]**
**Total (random effect, 95% CI)**							.	**15.8%**	**-1.22 [-2.55; 0.10]**
Heterogeneity: $Tau^2 = 1.5256$; $Chi^2 = 11.47$, df = 5 (P = 0.0428); $I^2 = 56.4\%$									
Test for overall effect (common effect): Z = -2.49 (P = 0.0128)									
Test for overall effect (random effects): Z = -1.81 (P = 0.0702)									
**Middle-aged and older adults (≥45 years)**									
Akasheh 2024	-2.20	1.7700	35	-0.60	1.8700	14	2.3%	5.5%	-1.60 [-2.74; -0.46]
Chow 2020	-1.70	10.5000	11	-0.90	13.1200	9	0.0%	0.1%	-0.80 [-11.38; 9.78]
Cienfuegos 2020a	-2.80	0.4000	16	-0.60	0.4000	7	23.8%	9.0%	-2.20 [-2.56; -1.84]
Cienfuegos 2020b	-1.40	0.3000	19	-0.60	0.4000	7	28.3%	9.1%	-0.80 [-1.13; -0.47]
Lowe 2020	-0.51	1.5800	22	-0.03	1.5700	24	3.6%	6.5%	-0.48 [-1.39; 0.43]
stekovic 2019	-2.20	1.3800	30	-0.20	0.9500	60	10.0%	8.2%	-2.01 [-2.55; -1.46]
Stote 2007	-2.00	2.7900	15	0.10	2.7900	15	0.8%	2.9%	-2.10 [-4.10; -0.10]
**Total (common effect, 95% CI)**			**148**			**136**	**68.8%**	.	**-1.48 [-1.69; -1.27]**
**Total (random effect, 95% CI)**							.	**41.2%**	**-1.49 [-2.19; -0.79]**
Heterogeneity: $Tau^2 = 0.5818$; $Chi^2 = 41.1$, df = 6 (P < 0.0001); $I^2 = 85.4\%$									
Test for overall effect (common effect): Z = -13.91 (P < 0.0001)									
Test for overall effect (random effects): Z = -4.16 (P < 0.0001)									
**Total (common effect, 95% CI)**			**482**			**401**	**100.0%**	.	**-1.36 [-1.53; -1.19]**
**Total (random effect, 95% CI)**							.	**100.0%**	**-1.26 [-1.66; -0.85]**
Heterogeneity: $Tau^2 = 0.4454$; $Chi^2 = 69.54$, df = 22 (P < 0.0001); $I^2 = 68.4\%$									
Test for subgroup differences (common effect): $Chi^2 = 4.36$, df = 2 (P = 0.1129)									
Test for subgroup differences (random effects): $Chi^2 = 0.89$, df = 2 (P = 0.6412)									

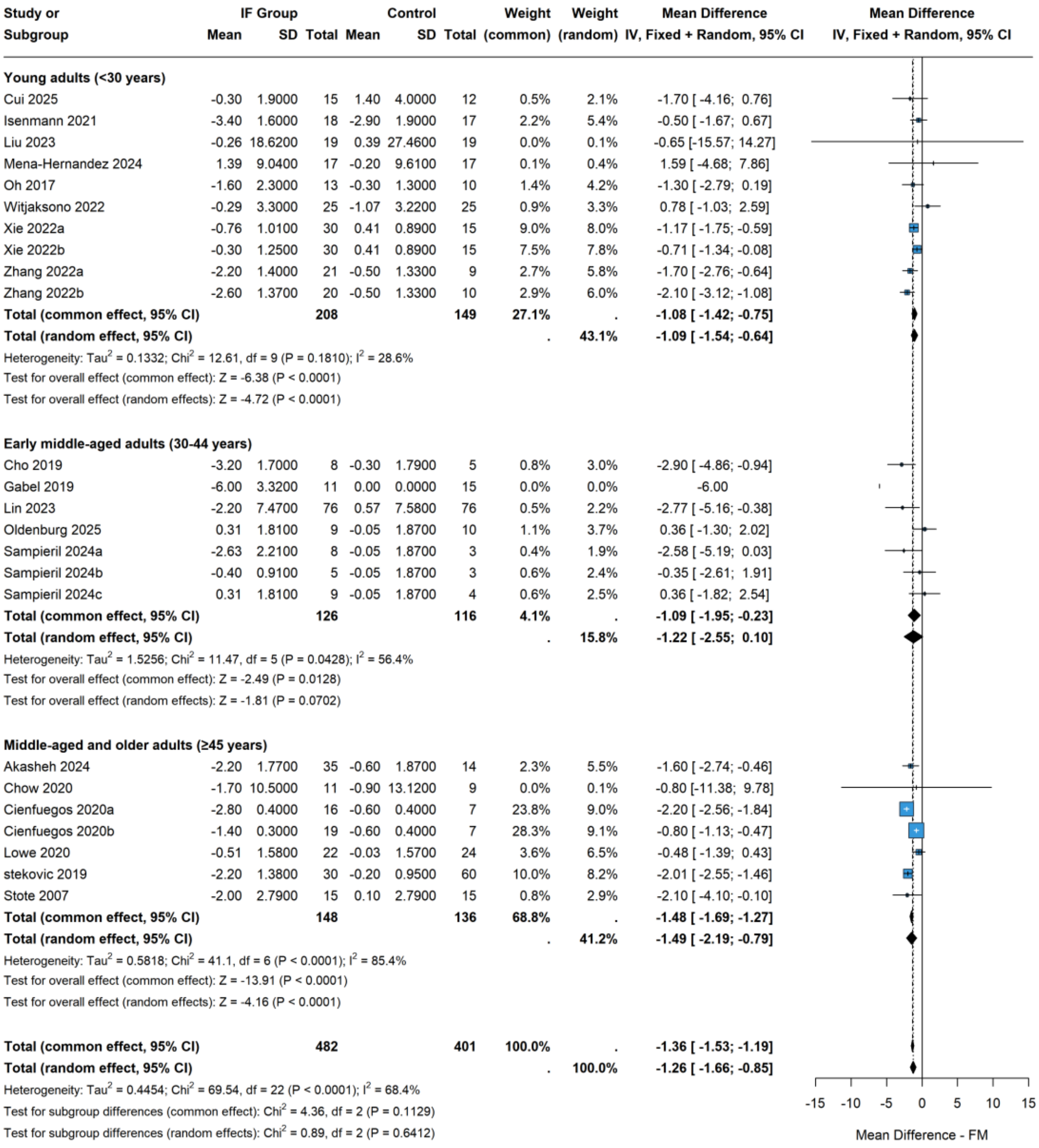

Figure S25. Age-stratified forest plot of the effect of intermittent fasting on fat-free mass (FFM).The forest plot illustrates the mean differences (MD) and 95% confidence intervals (CIs) comparing intermittent fasting interventions with control diets across three distinct age cohorts: young (<30 years), early middle-aged (30–44 years), and middle-aged and older (≥45 years) adults. The squares represent the point estimate of each individual trial, with the size of the square proportional to the study's assigned weight in the random-effects meta-analysis. The horizontal lines denote the 95% CIs. The diamonds represent the pooled effect estimates for each age subgroup and the overall analysis. The solid vertical line indicates the line of no effect (MD = 0).

Study or Subgroup	IF Group Mean	IF Group SD	IF Group Total	Control Mean	Control SD	Control Total	Weight (common)	Weight (random)	Mean Difference IV, Fixed + Random, 95% CI
**Young adults (<30 years)**									
Cui 2025	-2.30	2.2000	15	-1.60	4.1000	12	0.2%	1.4%	-0.70 [-3.27; 1.87]
Liu 2023	-2.77	17.6000	19	-1.13	16.8100	19	0.0%	0.1%	-1.64 [-12.58; 9.30]
Mena-Hernandez 2024	1.66	7.6000	17	-1.60	5.7500	17	0.1%	0.5%	3.26 [-1.27; 7.79]
Witjaksono 2022	-0.02	3.0700	25	2.20	9.5900	25	0.1%	0.6%	-2.22 [-6.17; 1.73]
Zhang 2022a	-1.30	1.0500	21	0.30	1.1100	9	2.2%	8.3%	-1.60 [-2.45; -0.75]
Zhang 2022b	-0.30	1.1400	20	0.30	1.1100	10	2.2%	8.3%	-0.60 [-1.45; 0.25]
**Total (common effect, 95% CI)**			**117**			**92**	**4.9%**	**.**	**-1.03 [-1.61; -0.46]**
**Total (random effect, 95% CI)**							**.**	**19.1%**	**-0.98 [-1.80; -0.16]**
Heterogeneity: $Tau^2 = 0.2311$; $Chi^2 = 6.57$, df = 5 (P = 0.2549); $I^2 = 23.9\%$									
Test for overall effect (common effect): Z = -3.53 (P = 0.0004)									
Test for overall effect (random effects): Z = -2.33 (P = 0.0196)									
**Early middle-aged adults (30-44 years)**									
Gabel 2019	-1.00	3.3200	11	0.00	3.8700	15	0.2%	1.2%	-1.00 [-3.77; 1.77]
Lin 2023	-0.41	2.2000	76	0.39	4.0000	76	1.5%	6.5%	-0.80 [-1.83; 0.23]
Oldenburg 2025	-0.60	3.0700	30	-0.60	3.0200	29	0.7%	3.4%	0.00 [-1.55; 1.55]
**Total (common effect, 95% CI)**			**117**			**120**	**2.4%**	**.**	**-0.60 [-1.41; 0.22]**
**Total (random effect, 95% CI)**							**.**	**11.1%**	**-0.60 [-1.41; 0.22]**
Heterogeneity: $Tau^2 = 0$; $Chi^2 = 0.8$, df = 2 (P = 0.6709); $I^2 = 0\%$									
Test for overall effect (common effect): Z = -1.43 (P = 0.1537)									
Test for overall effect (random effects): Z = -1.43 (P = 0.1537)									
**Middle-aged and older adults (≥45 years)**									
Akasheh 2024	-1.40	1.1800	35	-0.30	1.5000	14	2.1%	8.0%	-1.10 [-1.98; -0.22]
Chow 2020	-1.40	6.2300	11	-0.10	5.2900	9	0.1%	0.4%	-1.30 [-6.35; 3.75]
Cienfuegos 2020a	-0.80	0.4000	16	-0.30	0.2000	7	26.9%	18.8%	-0.50 [-0.75; -0.25]
Cienfuegos 2020b	-1.50	0.2000	19	-0.30	0.2000	7	54.1%	20.0%	-1.20 [-1.37; -1.03]
Lowe 2020	-1.10	1.5000	22	-0.35	1.5000	24	2.2%	8.1%	-0.75 [-1.62; 0.12]
stekovic 2019	-1.41	1.0100	30	-0.02	1.2100	60	7.2%	14.3%	-1.39 [-1.86; -0.92]
Stote 2007	0.80	10.0400	15	-0.70	10.0400	15	0.0%	0.2%	1.50 [-5.69; 8.69]
**Total (common effect, 95% CI)**			**148**			**136**	**92.6%**	**.**	**-1.00 [-1.13; -0.87]**
**Total (random effect, 95% CI)**							**.**	**69.8%**	**-0.98 [-1.39; -0.57]**
Heterogeneity: $Tau^2 = 0.1488$; $Chi^2 = 24.47$, df = 6 (P = 0.0004); $I^2 = 75.5\%$									
Test for overall effect (common effect): Z = -14.76 (P < 0.0001)									
Test for overall effect (random effects): Z = -4.73 (P < 0.0001)									
**Total (common effect, 95% CI)**			**382**			**348**	**100.0%**	**.**	**-0.99 [-1.12; -0.86]**
**Total (random effect, 95% CI)**							**.**	**100.0%**	**-0.94 [-1.25; -0.63]**
Heterogeneity: $Tau^2 = 0.1199$; $Chi^2 = 32.77$, df = 15 (P = 0.0051); $I^2 = 54.2\%$									
Test for subgroup differences (common effect): $Chi^2 = 0.93$, df = 2 (P = 0.6288)									
Test for subgroup differences (random effects): $Chi^2 = 0.71$, df = 2 (P = 0.7028)									

Mean Difference IV, Fixed + Random, 95% CI

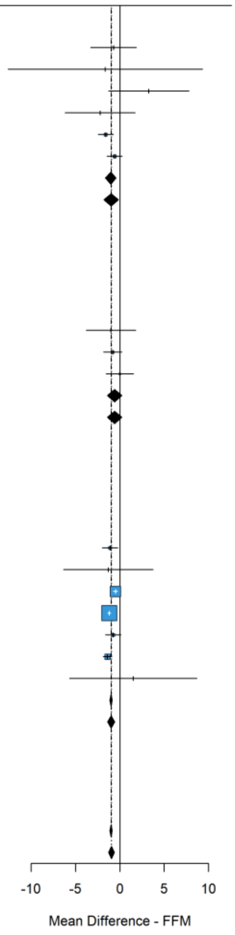

Figure S26. Age-stratified forest plot of the effect of intermittent fasting on total cholesterol (TC).The forest plot illustrates the mean differences (MD) and 95% confidence intervals (CIs) comparing intermittent fasting interventions with control diets across three distinct age cohorts: young (<30 years), early middle-aged (30–44 years), and middle-aged and older (≥45 years) adults. The squares represent the point estimate of each individual trial, with the size of the square proportional to the study's assigned weight in the random-effects meta-analysis. The horizontal lines denote the 95% CIs. The diamonds represent the pooled effect estimates for each age subgroup and the overall analysis. The solid vertical line indicates the line of no effect (MD = 0).

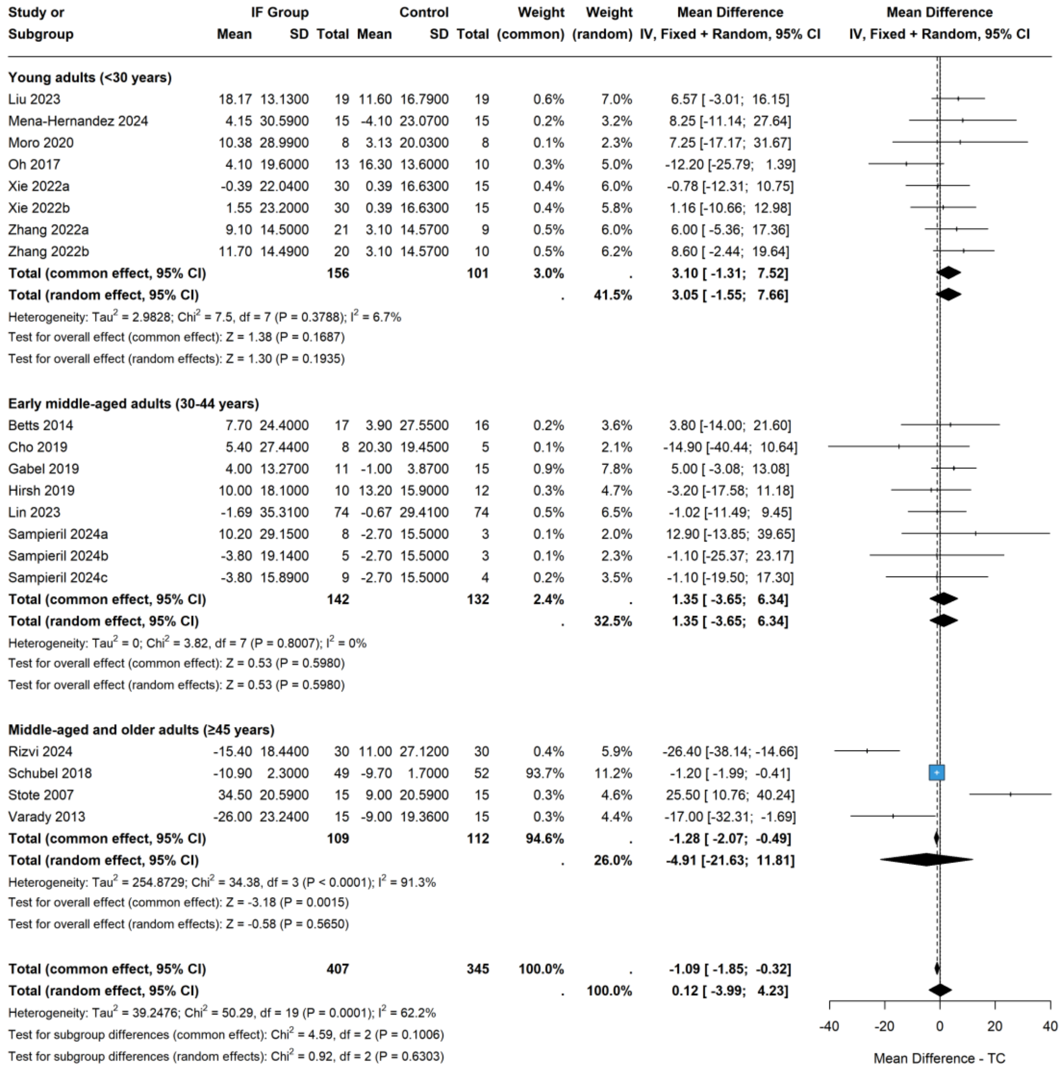

Figure S27. Age-stratified forest plot of the effect of intermittent fasting on triglycerides (TG).The forest plot illustrates the mean differences (MD) and 95% confidence intervals (CIs) comparing intermittent fasting interventions with control diets across three distinct age cohorts: young (<30 years), early middle-aged (30–44 years), and middle-aged and older (≥45 years) adults. The squares represent the point estimate of each individual trial, with the size of the square proportional to the study's assigned weight in the random-effects meta-analysis. The horizontal lines denote the 95% CIs. The diamonds represent the pooled effect estimates for each age subgroup and the overall analysis. The solid vertical line indicates the line of no effect (MD = 0).

Study or	IF Group			Control			Weight	Weight	Mean Difference
Subgroup	Mean	SD	Total	Mean	SD	Total	(common)	(random)	IV, Fixed + Random, 95% CI
**Young adults (<30 years)**									
Liu 2023	23.03	20.0800	19	5.31	27.1500	19	1.1%	4.3%	17.72 [2.54; 32.90]
Mena-Hernandez 2024	18.14	64.9200	15	0.27	46.8200	15	0.2%	0.7%	17.87 [-22.64; 58.38]
Moro 2020	-15.62	15.6900	8	-0.12	35.3000	8	0.3%	1.6%	-15.50 [-42.27; 11.27]
Oh 2017	12.00	56.2000	13	21.20	31.0000	10	0.2%	0.9%	-9.20 [-45.29; 26.89]
Xie 2022a	1.77	42.5100	30	6.20	33.6600	15	0.5%	2.1%	-4.43 [-27.27; 18.41]
Xie 2022b	9.74	38.3400	30	6.20	33.6600	15	0.5%	2.3%	3.54 [-18.33; 25.41]
Zhang 2022a	13.60	30.9800	21	10.90	31.0200	9	0.4%	1.9%	2.70 [-21.51; 26.91]
Zhang 2022b	2.50	31.0300	20	10.90	31.0200	10	0.4%	2.0%	-8.40 [-31.95; 15.15]
**Total (common effect, 95% CI)**			**156**			**101**	**3.6%**	.	**3.24 [-5.03; 11.51]**
**Total (random effect, 95% CI)**							.	**15.7%**	**2.69 [-6.13; 11.51]**
Heterogeneity: Tau^2 = 15.1478; Chi^2 = 7.71, df = 7 (P = 0.3590); I^2 = 9.2%									
Test for overall effect (common effect): Z = 0.77 (P = 0.4422)									
Test for overall effect (random effects): Z = 0.60 (P = 0.5502)									
**Early middle-aged adults (30-44 years)**									
Betts 2014	-5.30	22.3000	17	-0.88	22.5500	16	1.1%	4.2%	-4.42 [-19.73; 10.89]
Cho 2019	12.60	98.1500	8	53.20	69.3200	5	0.0%	0.1%	-40.60 [-131.80; 50.60]
Gabel 2019	-27.00	59.7000	11	-8.00	19.3600	15	0.2%	0.9%	-19.00 [-55.61; 17.61]
Lin 2023	3.60	60.6600	74	-0.08	78.4100	74	0.5%	2.1%	3.68 [-18.91; 26.27]
Manoogian 2022	-1.96	39.1900	70	-3.12	37.4300	66	1.5%	5.5%	1.16 [-11.72; 14.04]
Oldenburg 2025	3.70	47.1200	29	3.00	47.0900	26	0.4%	1.8%	0.70 [-24.23; 25.63]
Sampieril 2024a	-1.90	18.4100	8	9.70	24.1100	3	0.3%	1.3%	-11.60 [-41.72; 18.52]
Sampieril 2024b	10.40	54.5800	5	9.70	24.1100	3	0.1%	0.4%	0.70 [-54.37; 55.77]
Sampieril 2024c	13.10	33.7900	9	9.70	24.1100	4	0.2%	1.1%	3.40 [-28.94; 35.74]
**Total (common effect, 95% CI)**			**231**			**212**	**4.3%**	.	**-1.86 [-9.51; 5.78]**
**Total (random effect, 95% CI)**							.	**17.5%**	**-1.86 [-9.51; 5.78]**
Heterogeneity: Tau^2 = 0; Chi^2 = 2.64, df = 8 (P = 0.9550); I^2 = 0%									
Test for overall effect (common effect): Z = -0.48 (P = 0.6330)									
Test for overall effect (random effects): Z = -0.48 (P = 0.6330)									
**Middle-aged and older adults (≥45 years)**									
Chow 2020	-38.00	32.6700	11	0.00	16.2000	9	0.5%	2.2%	-38.00 [-60.02; -15.98]
Cienfuegos 2020a	-1.90	6.7000	16	4.50	3.2000	7	15.2%	17.7%	-6.40 [-10.45; -2.35]
Cienfuegos 2020b	2.60	5.7000	19	4.50	3.2000	7	20.5%	18.8%	-1.90 [-5.39; 1.59]
Rizvi 2024	-7.50	90.6800	30	21.50	132.7600	30	0.1%	0.4%	-29.00 [-86.53; 28.53]
Schubel 2018	-20.70	6.0000	49	-13.20	4.9000	52	54.3%	21.4%	-7.50 [-9.64; -5.36]
Stote 2007	-3.90	20.2400	15	5.10	20.2400	15	1.2%	4.6%	-9.00 [-23.49; 5.49]
Varady 2013	-22.00	42.6000	15	10.00	27.1100	15	0.4%	1.7%	-32.00 [-57.55; -6.45]
**Total (common effect, 95% CI)**			**155**			**135**	**92.1%**	.	**-6.38 [-8.03; -4.74]**
**Total (random effect, 95% CI)**							.	**66.8%**	**-7.83 [-12.47; -3.20]**
Heterogeneity: Tau^2 = 16.9860; Chi^2 = 19.88, df = 6 (P = 0.0029); I^2 = 69.8%									
Test for overall effect (common effect): Z = -7.60 (P < 0.0001)									
Test for overall effect (random effects): Z = -3.32 (P = 0.0009)									
**Total (common effect, 95% CI)**			**542**			**448**	**100.0%**	.	**-5.84 [-7.42; -4.26]**
**Total (random effect, 95% CI)**							.	**100.0%**	**-4.94 [-8.42; -1.47]**
Heterogeneity: Tau^2 = 13.5107; Chi^2 = 36.32, df = 23 (P = 0.0383); I^2 = 36.7%									
Test for subgroup differences (common effect): Chi^2 = 6.09, df = 2 (P = 0.0475)									
Test for subgroup differences (random effects): Chi^2 = 4.98, df = 2 (P = 0.0827)									

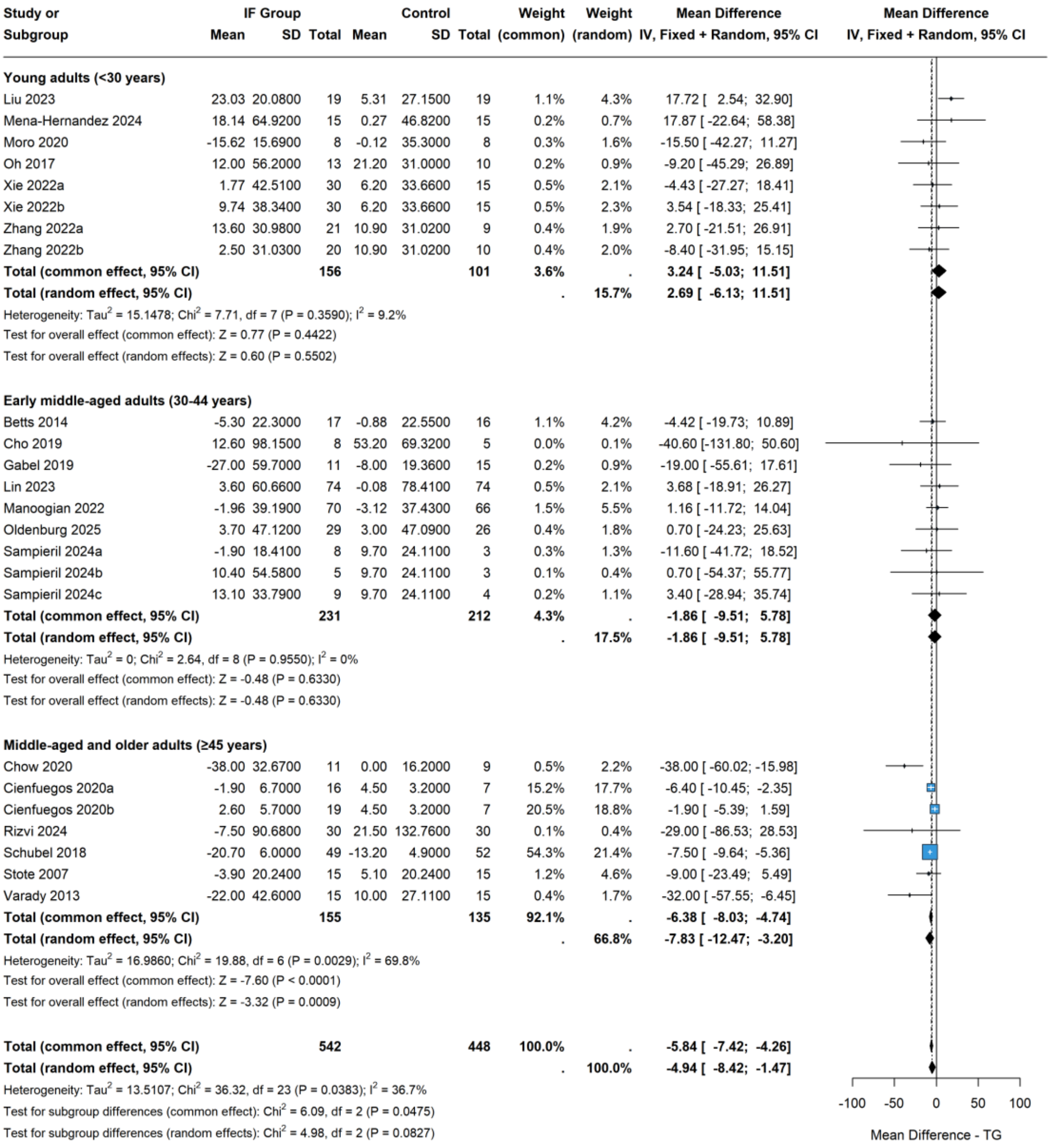

Figure S28. Age-stratified forest plot of the effect of intermittent fasting on high-density lipoprotein cholesterol (HDL-C).The forest plot illustrates the mean differences (MD) and 95% confidence intervals (CIs) comparing intermittent fasting interventions with control diets across three distinct age cohorts: young (<30 years), early middle-aged (30–44 years), and middle-aged and older (≥45 years) adults. The squares represent the point estimate of each individual trial, with the size of the square proportional to the study's assigned weight in the random-effects meta-analysis. The horizontal lines denote the 95% CIs. The diamonds represent the pooled effect estimates for each age subgroup and the overall analysis. The solid vertical line indicates the line of no effect (MD = 0).

Study or Subgroup	IF Group Mean	SD	Total	Control Mean	SD	Total	Weight (common)	Weight (random)	Mean Difference IV, Fixed + Random, 95% CI
**Young adults (<30 years)**									
Liu 2023	8.12	8.8400	19	6.57	10.7200	19	0.7%	2.0%	1.55 [-4.70; 7.80]
Mena-Hernandez 2024	-3.20	5.1300	15	1.10	7.2900	15	1.4%	3.5%	-4.30 [-8.81; 0.21]
Oh 2017	2.60	8.3000	13	4.90	4.7000	10	1.0%	2.7%	-2.30 [-7.67; 3.07]
Xie 2022a	-1.16	7.7300	30	1.93	6.5700	15	1.5%	3.8%	-3.09 [-7.42; 1.24]
Xie 2022b	-0.01	0.1900	30	1.93	6.5700	15	2.6%	5.5%	-1.94 [-5.27; 1.39]
Zhang 2022a	2.80	2.6900	21	2.70	3.6700	9	4.0%	7.3%	0.10 [-2.56; 2.76]
Zhang 2022b	3.60	4.5600	20	2.70	3.6700	10	3.1%	6.2%	0.90 [-2.13; 3.93]
**Total (common effect, 95% CI)**			**148**			**93**	**14.4%**	.	**-0.95 [-2.36; 0.46]**
**Total (random effect, 95% CI)**							.	**31.0%**	**-0.98 [-2.43; 0.47]**
Heterogeneity: Tau^2 = 0.1842; Chi^2 = 6.29, df = 6 (P = 0.3915); I^2 = 4.6%									
Test for overall effect (common effect): Z = -1.32 (P = 0.1853)									
Test for overall effect (random effects): Z = -1.32 (P = 0.1868)									
**Early middle-aged adults (30-44 years)**									
Betts 2014	3.90	6.1000	17	3.90	6.6300	16	1.5%	3.7%	0.00 [-4.35; 4.35]
Cho 2019	2.90	8.4900	8	5.70	8.5000	5	0.3%	1.0%	-2.80 [-12.29; 6.69]
Gabel 2019	7.00	13.2700	14	0.00	15.4900	15	0.3%	0.8%	7.00 [-3.48; 17.48]
Hirsh 2019	8.00	10.0000	10	3.00	5.0000	12	0.6%	1.8%	5.00 [-1.81; 11.81]
Lin 2023	-1.53	13.0600	74	-2.88	11.0600	74	1.9%	4.4%	1.35 [-2.55; 5.25]
Manoogian 2022	0.13	6.9800	70	1.27	8.1900	66	4.3%	7.6%	-1.14 [-3.70; 1.42]
Oldenburg 2025	-1.80	13.8800	29	3.80	13.9200	26	0.5%	1.5%	-5.60 [-12.96; 1.76]
Sampieril 2024a	0.40	6.5100	8	-1.10	4.7000	3	0.6%	1.7%	1.50 [-5.47; 8.47]
Sampieril 2024b	-6.30	5.7200	5	-1.10	4.7000	3	0.5%	1.5%	-5.20 [-12.51; 2.11]
Sampieril 2024c	-1.40	6.6200	9	-1.10	4.7000	4	0.7%	2.0%	-0.30 [-6.62; 6.02]
**Total (common effect, 95% CI)**			**244**			**224**	**11.3%**	.	**-0.31 [-1.90; 1.28]**
**Total (random effect, 95% CI)**							.	**26.0%**	**-0.27 [-1.96; 1.43]**
Heterogeneity: Tau^2 = 0.4576; Chi^2 = 9.55, df = 9 (P = 0.3882); I^2 = 5.8%									
Test for overall effect (common effect): Z = -0.38 (P = 0.7047)									
Test for overall effect (random effects): Z = -0.31 (P = 0.7574)									
**Middle-aged and older adults (≥45 years)**									
Chow 2020	1.00	8.8500	11	7.00	11.7400	9	0.3%	1.0%	-6.00 [-15.28; 3.28]
Cienfuegos 2020a	-2.40	1.3000	16	-0.70	1.0000	7	29.8%	14.2%	-1.70 [-2.68; -0.72]
Cienfuegos 2020b	0.80	1.4000	19	4.50	3.2000	7	4.7%	7.9%	-3.70 [-6.15; -1.25]
Rizvi 2024	2.50	10.5700	30	1.50	13.6200	30	0.7%	2.1%	1.00 [-5.17; 7.17]
Schubel 2018	-8.70	2.5000	49	-9.20	1.9000	52	37.6%	14.6%	0.50 [-0.37; 1.37]
Stote 2007	8.40	10.4100	15	3.20	10.4100	15	0.5%	1.5%	5.20 [-2.25; 12.65]
Varady 2013	-2.00	11.6200	15	1.00	7.7500	15	0.6%	1.6%	-3.00 [-10.07; 4.07]
**Total (common effect, 95% CI)**			**155**			**135**	**74.4%**	.	**-0.67 [-1.29; -0.05]**
**Total (random effect, 95% CI)**							.	**43.0%**	**-1.14 [-2.95; 0.67]**
Heterogeneity: Tau^2 = 2.6585; Chi^2 = 21.43, df = 6 (P = 0.0015); I^2 = 72%									
Test for overall effect (common effect): Z = -2.12 (P = 0.0343)									
Test for overall effect (random effects): Z = -1.23 (P = 0.2185)									
**Total (common effect, 95% CI)**			**547**			**452**	**100.0%**	.	**-0.67 [-1.20; -0.13]**
**Total (random effect, 95% CI)**							.	**100.0%**	**-0.87 [-1.83; 0.08]**
Heterogeneity: Tau^2 = 1.4264; Chi^2 = 37.62, df = 23 (P = 0.0279); I^2 = 38.9%									
Test for subgroup differences (common effect): Chi^2 = 0.35, df = 2 (P = 0.8381)									
Test for subgroup differences (random effects): Chi^2 = 0.57, df = 2 (P = 0.7520)									

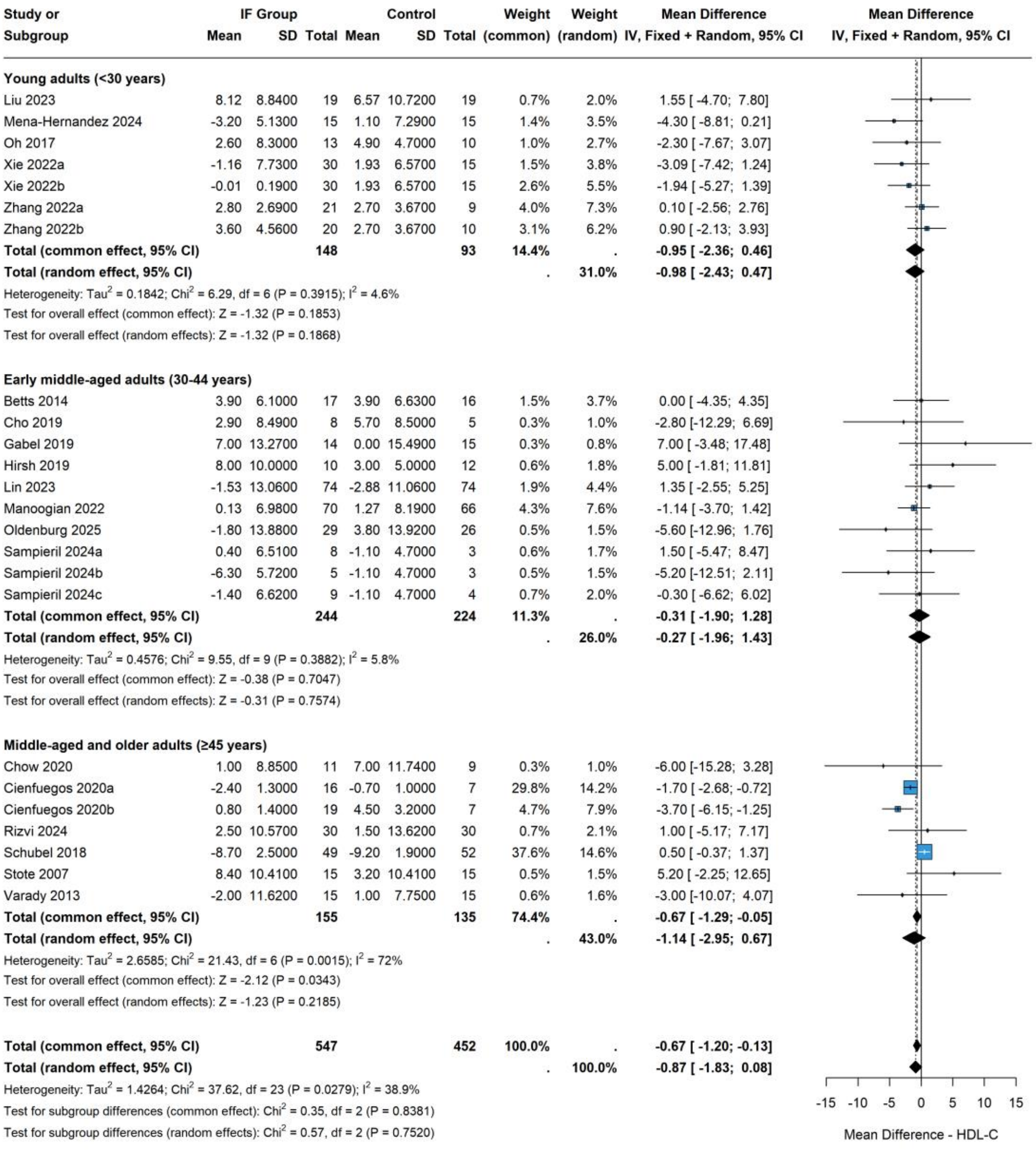

Figure S29. Age-stratified forest plot of the effect of intermittent fasting on low-density lipoprotein cholesterol (LDL-C).The forest plot illustrates the mean differences (MD) and 95% confidence intervals (CIs) comparing intermittent fasting interventions with control diets across three distinct age cohorts: young (<30 years), early middle-aged (30–44 years), and middle-aged and older (≥45 years) adults. The squares represent the point estimate of each individual trial, with the size of the square proportional to the study's assigned weight in the random-effects meta-analysis. The horizontal lines denote the 95% CIs. The diamonds represent the pooled effect estimates for each age subgroup and the overall analysis. The solid vertical line indicates the line of no effect (MD = 0).

Study or Subgroup	IF Group Mean	IF Group SD	IF Group Total	Control Mean	Control SD	Control Total	Weight (common)	Weight (random)	Mean Difference IV, Fixed + Random, 95% CI
**Young adults (<30 years)**									
Liu 2023	17.79	17.3200	19	5.41	18.5000	19	0.6%	3.3%	12.38 [0.98; 23.78]
Mena-Hernandez 2024	4.70	18.6500	15	-6.10	19.3200	15	0.4%	2.5%	10.80 [-2.79; 24.39]
Xie 2022a	0.00	18.9500	30	-3.09	13.9200	15	0.8%	4.1%	3.09 [-6.69; 12.87]
Xie 2022b	0.39	17.7900	30	-3.09	13.9200	15	0.8%	4.3%	3.48 [-6.01; 12.97]
Zhang 2022a	19.20	8.8800	21	12.50	8.9000	9	1.5%	6.2%	6.70 [-0.25; 13.65]
Zhang 2022b	19.70	8.7800	20	12.50	8.9000	10	1.6%	6.4%	7.20 [0.47; 13.93]
**Total (common effect, 95% CI)**			**135**			**83**	**5.7%**	.	**6.75 [3.15; 10.34]**
**Total (random effect, 95% CI)**							.	**26.9%**	**6.75 [3.15; 10.34]**
Heterogeneity: $Tau^2 = 0$; $Chi^2 = 2.29$, df = 5 (P = 0.8077); $I^2 = 0\%$									
Test for overall effect (common effect): Z = 3.68 (P = 0.0002)									
Test for overall effect (random effects): Z = 3.68 (P = 0.0002)									
**Early middle-aged adults (30-44 years)**									
Betts 2014	3.90	20.3000	17	0.40	23.6700	16	0.3%	2.1%	3.50 [-11.59; 18.59]
Cho 2019	0.00	28.0000	8	16.90	28.1700	5	0.1%	0.6%	-16.90 [-48.30; 14.50]
Gabel 2019	7.00	13.2700	11	0.00	15.4900	15	0.6%	3.4%	7.00 [-4.09; 18.09]
Hirsh 2019	5.20	18.3000	10	9.30	14.1000	12	0.4%	2.4%	-4.10 [-17.97; 9.77]
Lin 2023	-0.86	36.3400	74	2.37	30.1700	74	0.6%	3.6%	-3.23 [-13.99; 7.53]
Manoogian 2022	0.75	18.2000	69	-2.61	19.5400	66	1.8%	6.8%	3.36 [-3.02; 9.74]
Oldenburg 2025	-0.10	17.7200	29	-4.20	17.5600	26	0.8%	4.4%	4.10 [-5.24; 13.44]
Sampieril 2024a	9.20	28.1900	8	-1.10	4.7000	3	0.2%	1.3%	10.30 [-9.95; 30.55]
Sampieril 2024b	-2.10	16.5700	5	-1.10	4.7000	3	0.3%	2.0%	-1.00 [-16.47; 14.47]
Sampieril 2024c	-4.90	13.0200	9	-1.10	4.7000	4	0.8%	4.2%	-3.80 [-13.47; 5.87]
**Total (common effect, 95% CI)**			**240**			**224**	**5.9%**	.	**1.44 [-2.08; 4.96]**
**Total (random effect, 95% CI)**							.	**30.8%**	**1.44 [-2.08; 4.96]**
Heterogeneity: $Tau^2 = 0$; $Chi^2 = 6.3$, df = 9 (P = 0.7092); $I^2 = 0\%$									
Test for overall effect (common effect): Z = 0.80 (P = 0.4238)									
Test for overall effect (random effects): Z = 0.80 (P = 0.4238)									
**Middle-aged and older adults (≥45 years)**									
Chow 2020	9.00	18.6200	11	1.00	12.0200	9	0.4%	2.5%	8.00 [-5.52; 21.52]
Cienfuegos 2020a	2.60	5.7000	16	-2.00	3.7000	7	4.8%	9.7%	4.60 [0.69; 8.51]
Cienfuegos 2020b	-4.80	5.1000	19	-2.00	3.7000	7	5.7%	10.1%	-2.80 [-6.37; 0.77]
Rizvi 2024	-9.90	16.5300	30	10.50	30.2000	30	0.5%	2.9%	-20.40 [-32.72; -8.08]
Schubel 2018	-7.50	2.6000	49	-8.70	2.4000	52	76.4%	12.8%	1.20 [0.22; 2.18]
Stote 2007	27.10	22.8900	15	4.20	22.8900	15	0.3%	1.8%	22.90 [6.52; 39.28]
Varady 2013	-18.00	23.2400	15	-9.00	15.4900	15	0.4%	2.4%	-9.00 [-23.13; 5.13]
**Total (common effect, 95% CI)**			**155**			**135**	**88.4%**	.	**1.06 [0.15; 1.97]**
**Total (random effect, 95% CI)**							.	**42.2%**	**0.19 [-4.32; 4.70]**
Heterogeneity: $Tau^2 = 20.2335$; $Chi^2 = 29.15$, df = 6 (P < 0.0001); $I^2 = 79.4\%$									
Test for overall effect (common effect): Z = 2.29 (P = 0.0219)									
Test for overall effect (random effects): Z = 0.08 (P = 0.9344)									
**Total (common effect, 95% CI)**			**530**			**442**	**100.0%**	.	**1.41 [0.55; 2.26]**
**Total (random effect, 95% CI)**							.	**100.0%**	**2.33 [-0.07; 4.73]**
Heterogeneity: $Tau^2 = 11.5195$; $Chi^2 = 46.78$, df = 22 (P = 0.0016); $I^2 = 53.0\%$									
Test for subgroup differences (common effect): $Chi^2 = 9.04$, df = 2 (P = 0.0109)									
Test for subgroup differences (random effects): $Chi^2 = 6.42$, df = 2 (P = 0.0404)									

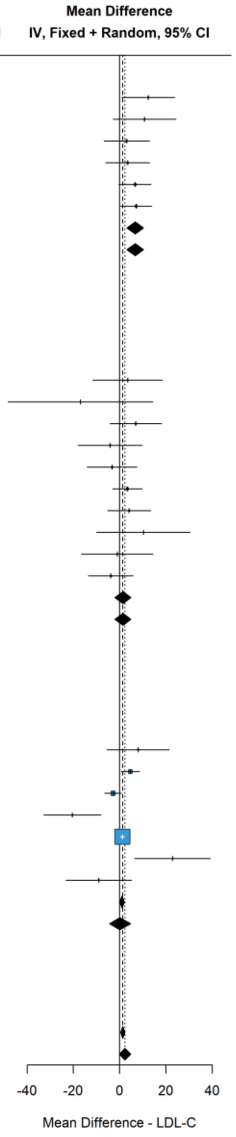

Figure S30. Age-stratified forest plot of the effect of intermittent fasting on fasting insulin (FINS).The forest plot illustrates the mean differences (MD) and 95% confidence intervals (CIs) comparing intermittent fasting interventions with control diets across three distinct age cohorts: young (<30 years), early middle-aged (30–44 years), and middle-aged and older (≥45 years) adults. The squares represent the point estimate of each individual trial, with the size of the square proportional to the study's assigned weight in the random-effects meta-analysis. The horizontal lines denote the 95% CIs. The diamonds represent the pooled effect estimates for each age subgroup and the overall analysis. The solid vertical line indicates the line of no effect (MD = 0).

Study or Subgroup	IF Group Mean	IF Group SD	IF Group Total	Control Mean	Control SD	Control Total	Weight (common)	Weight (random)	Mean Difference IV, Fixed + Random, 95% CI
**Young adults (<30 years)**									
Mena-Hernandez 2024	5.22	6.9900	15	7.00	5.2800	15	1.0%	4.1%	-1.78 [-6.21; 2.65]
Moro 2020	-1.37	1.1900	8	-0.13	1.8700	8	8.0%	6.4%	-1.24 [-2.78; 0.30]
Oh 2017	2.60	11.5000	13	2.60	4.0000	10	0.4%	2.7%	0.00 [-6.73; 6.73]
Zhang 2022a	-3.40	3.8600	21	0.40	3.8900	9	2.1%	5.3%	-3.80 [-6.83; -0.77]
Zhang 2022b	-1.60	3.8800	20	0.40	3.8900	10	2.2%	5.3%	-2.00 [-4.95; 0.95]
**Total (common effect, 95% CI)**			**77**			**52**	**13.6%**	.	**-1.75 [-2.93; -0.57]**
**Total (random effect, 95% CI)**							.	**23.8%**	**-1.75 [-2.93; -0.57]**
Heterogeneity: $Tau^2 = 0$; $Chi^2 = 2.47$, df = 4 (P = 0.6502); $I^2 = 0\%$									
Test for overall effect (common effect): Z = -2.91 (P = 0.0036)									
Test for overall effect (random effects): Z = -2.91 (P = 0.0036)									
**Early middle-aged adults (30-44 years)**									
Betts 2014	0.32	2.0700	17	0.35	1.3500	16	13.4%	6.6%	-0.03 [-1.22; 1.16]
Cho 2019	3.21	8.8000	8	5.19	8.8800	5	0.2%	1.6%	-1.98 [-11.87; 7.91]
Gabel 2019	-12.00	13.2700	11	-3.00	19.3600	15	0.1%	1.1%	-9.00 [-21.55; 3.55]
Hirsh 2019	-0.50	2.2000	10	3.00	4.0000	12	2.7%	5.6%	-3.50 [-6.14; -0.86]
Lin 2023	-2.83	11.0000	74	1.44	16.5200	74	0.9%	4.1%	-4.27 [-8.79; 0.25]
Manoogian 2022	-0.06	2.6700	70	0.14	2.1300	66	28.8%	6.8%	-0.20 [-1.01; 0.61]
Oldenburg 2025	-4.60	13.3300	29	-6.40	13.2700	26	0.4%	2.6%	1.80 [-5.24; 8.84]
Sampieril 2024a	0.75	2.5300	8	-0.90	1.3800	3	3.4%	5.8%	1.65 [-0.70; 4.00]
Sampieril 2024b	-0.98	4.6000	5	-0.90	1.3800	3	1.0%	4.2%	-0.08 [-4.40; 4.24]
Sampieril 2024c	1.80	3.3900	9	-0.90	1.3800	4	2.8%	5.6%	2.70 [0.10; 5.30]
Trepanowski 2019	-4.90	11.6500	35	-0.70	7.7400	34	0.9%	4.0%	-4.20 [-8.85; 0.45]
**Total (common effect, 95% CI)**			**276**			**258**	**54.6%**	.	**-0.20 [-0.79; 0.39]**
**Total (random effect, 95% CI)**							.	**47.9%**	**-0.46 [-1.68; 0.77]**
Heterogeneity: $Tau^2 = 1.5896$; $Chi^2 = 21.53$, df = 10 (P = 0.0177); $I^2 = 53.6\%$									
Test for overall effect (common effect): Z = -0.66 (P = 0.5073)									
Test for overall effect (random effects): Z = -0.73 (P = 0.4638)									
**Middle-aged and older adults (≥45 years)**									
Akasheh 2024	-2.24	5.8000	35	4.75	6.2100	14	1.3%	4.6%	-6.99 [-10.77; -3.21]
Chow 2020	0.00	4.2200	11	0.00	4.2200	9	1.4%	4.7%	0.00 [-3.72; 3.72]
Cienfuegos 2020a	-2.30	1.5000	16	3.50	1.4000	7	11.7%	6.6%	-5.80 [-7.07; -4.53]
Cienfuegos 2020b	-1.90	1.1000	19	3.50	1.4000	7	14.3%	6.6%	-5.40 [-6.55; -4.25]
Schubel 2018	-6.30	6.6000	49	-9.10	5.7000	52	3.2%	5.8%	2.80 [0.39; 5.21]
**Total (common effect, 95% CI)**			**130**			**89**	**31.9%**	.	**-4.55 [-5.32; -3.78]**
**Total (random effect, 95% CI)**							.	**28.3%**	**-3.15 [-6.23; -0.07]**
Heterogeneity: $Tau^2 = 10.6035$; $Chi^2 = 48.86$, df = 4 (P < 0.0001); $I^2 = 91.8\%$									
Test for overall effect (common effect): Z = -11.59 (P < 0.0001)									
Test for overall effect (random effects): Z = -2.01 (P = 0.0448)									
**Total (common effect, 95% CI)**			**483**			**399**	**100.0%**	.	**-1.79 [-2.23; -1.36]**
**Total (random effect, 95% CI)**							.	**100.0%**	**-1.75 [-3.16; -0.34]**
Heterogeneity: $Tau^2 = 7.4929$; $Chi^2 = 150.40$, df = 20 (P < 0.0001); $I^2 = 86.7\%$									
Test for subgroup differences (common effect): $Chi^2 = 77.54$, df = 2 (P < 0.0001)									
Test for subgroup differences (random effects): $Chi^2 = 3.77$, df = 2 (P = 0.1520)									

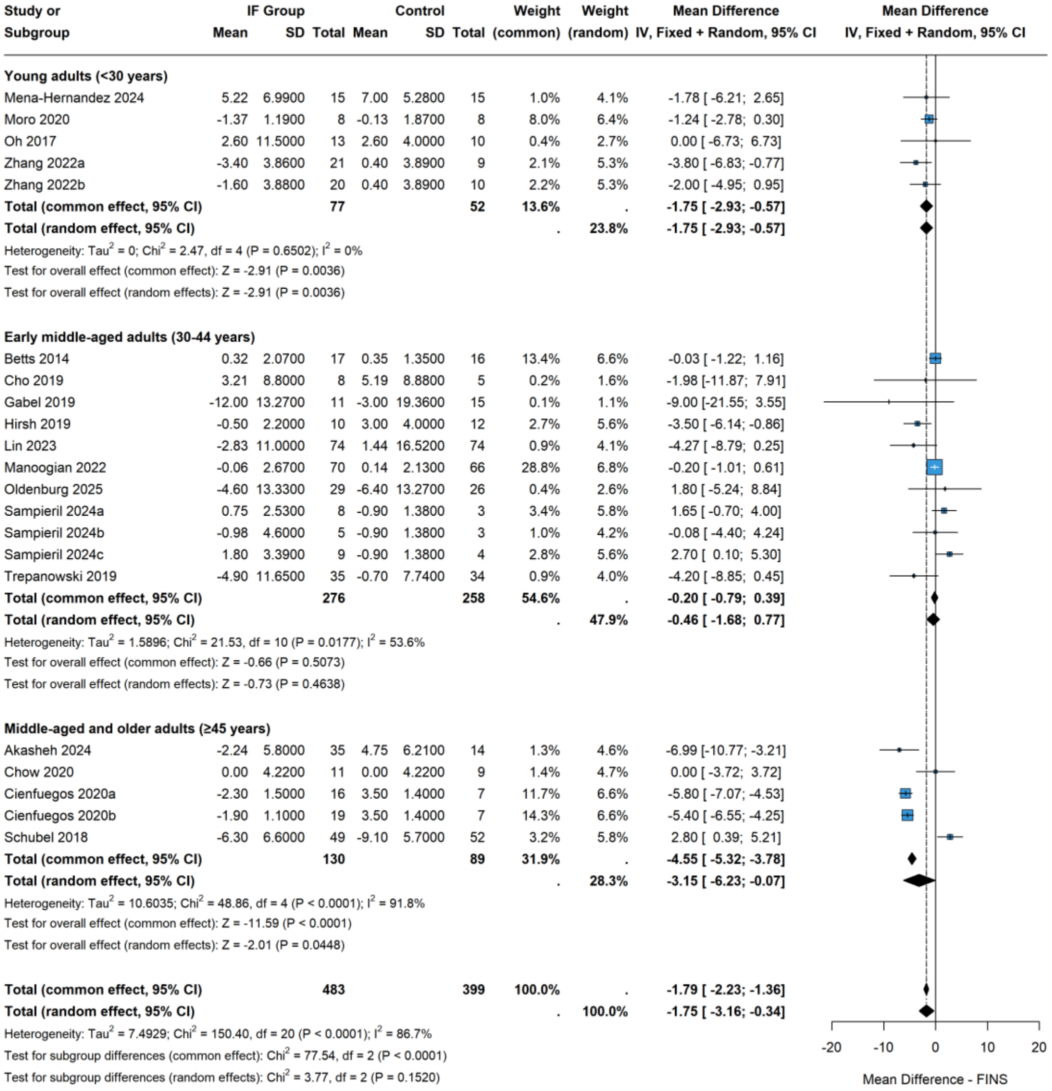

Figure S31. Age-stratified forest plot of the effect of intermittent fasting on fasting blood glucose (FBG).The forest plot illustrates the mean differences (MD) and 95% confidence intervals (CIs) comparing intermittent fasting interventions with control diets across three distinct age cohorts: young (<30 years), early middle-aged (30–44 years), and middle-aged and older (≥45 years) adults. The squares represent the point estimate of each individual trial, with the size of the square proportional to the study's assigned weight in the random-effects meta-analysis. The horizontal lines denote the 95% CIs. The diamonds represent the pooled effect estimates for each age subgroup and the overall analysis. The solid vertical line indicates the line of no effect (MD = 0).

Study or	IF Group			Control			Weight	Weight	Mean Difference
Subgroup	Mean	SD	Total	Mean	SD	Total	(common)	(random)	IV, Fixed + Random, 95% CI
**Young adults (<30 years)**									
Mena-Hernandez 2024	5.00	5.6700	15	-2.00	8.7000	15	0.7%	5.3%	7.00 [1.74; 12.26]
Moro 2020	-4.38	3.9300	8	0.25	4.1800	8	1.3%	6.1%	-4.63 [-8.61; -0.65]
Oh 2017	-3.00	9.4000	13	-1.00	5.0000	10	0.6%	4.8%	-2.00 [-7.98; 3.98]
Zhang 2022a	1.50	5.0300	21	2.50	5.0000	9	1.3%	6.2%	-1.00 [-4.91; 2.91]
Zhang 2022b	3.40	5.0200	20	2.50	5.0000	10	1.4%	6.3%	0.90 [-2.90; 4.70]
**Total (common effect, 95% CI)**			**77**			**52**	**5.3%**	.	**-0.38 [-2.33; 1.58]**
**Total (random effect, 95% CI)**							.	**28.6%**	**-0.08 [-3.65; 3.49]**
Heterogeneity: $Tau^2 = 11.1923$; $Chi^2 = 12.78$, df = 4 (P = 0.0124); $I^2 = 68.7\%$									
Test for overall effect (common effect): Z = -0.38 (P = 0.7061)									
Test for overall effect (random effects): Z = -0.05 (P = 0.9637)									
**Early middle-aged adults (30-44 years)**									
Betts 2014	1.30	5.6800	17	1.10	9.1800	16	0.7%	5.3%	0.20 [-5.05; 5.45]
Cho 2019	-9.70	11.3100	8	-1.30	11.1800	5	0.1%	2.0%	-8.40 [-20.95; 4.15]
Gabel 2019	-3.00	9.9500	11	4.00	15.4900	15	0.2%	2.9%	-7.00 [-16.80; 2.80]
Lin 2023	-1.65	17.4200	74	2.88	24.6000	74	0.4%	4.3%	-4.53 [-11.40; 2.34]
Manoogian 2022	0.19	7.0600	70	0.14	7.0300	66	3.6%	7.2%	0.05 [-2.32; 2.42]
Oldenburg 2025	-0.70	8.3800	29	-2.20	8.4600	26	1.0%	5.8%	1.50 [-2.96; 5.96]
Sampieril 2024a	-0.20	7.3300	8	-6.20	8.1600	3	0.2%	2.6%	6.00 [-4.54; 16.54]
Sampieril 2024b	-2.80	4.9600	5	-6.20	8.1600	3	0.2%	2.7%	3.40 [-6.81; 13.61]
Sampieril 2024c	-0.90	9.1700	9	-6.20	8.1600	4	0.2%	2.8%	5.30 [-4.69; 15.29]
Trepanowski 2019	-0.90	8.5300	35	-0.20	6.4800	34	1.6%	6.4%	-0.70 [-4.27; 2.87]
**Total (common effect, 95% CI)**			**266**			**246**	**8.3%**	.	**-0.11 [-1.67; 1.45]**
**Total (random effect, 95% CI)**							.	**42.0%**	**-0.11 [-1.67; 1.45]**
Heterogeneity: $Tau^2 = 0$; $Chi^2 = 8.68$, df = 9 (P = 0.4677); $I^2 = 0\%$									
Test for overall effect (common effect): Z = -0.14 (P = 0.8883)									
Test for overall effect (random effects): Z = -0.14 (P = 0.8883)									
**Middle-aged and older adults (≥45 years)**									
Akasheh 2024	-3.78	12.5400	35	3.63	13.4700	14	0.3%	3.6%	-7.41 [-15.60; 0.78]
Chow 2020	-8.00	6.0800	11	-7.00	10.7500	9	0.3%	3.7%	-1.00 [-8.89; 6.89]
Cienfuegos 2020a	-5.00	3.8000	16	2.60	2.6000	7	2.8%	7.0%	-7.60 [-10.28; -4.92]
Cienfuegos 2020b	-2.30	2.0000	19	2.60	2.6000	7	4.5%	7.3%	-4.90 [-7.03; -2.77]
Schubel 2018	-2.90	1.2000	49	-5.20	1.4000	52	78.5%	7.9%	2.30 [1.79; 2.81]
**Total (common effect, 95% CI)**			**130**			**89**	**86.4%**	.	**1.56 [1.07; 2.04]**
**Total (random effect, 95% CI)**							.	**29.4%**	**-3.57 [-8.96; 1.82]**
Heterogeneity: $Tau^2 = 31.8856$; $Chi^2 = 93.57$, df = 4 (P < 0.0001); $I^2 = 95.7\%$									
Test for overall effect (common effect): Z = 6.31 (P < 0.0001)									
Test for overall effect (random effects): Z = -1.30 (P = 0.1939)									
**Total (common effect, 95% CI)**			**473**			**387**	**100.0%**	.	**1.32 [0.87; 1.77]**
**Total (random effect, 95% CI)**							.	**100.0%**	**-1.14 [-3.22; 0.95]**
Heterogeneity: $Tau^2 = 14.3268$; $Chi^2 = 122.08$, df = 19 (P < 0.0001); $I^2 = 84.4\%$									
Test for subgroup differences (common effect): $Chi^2 = 7.05$, df = 2 (P = 0.0294)									
Test for subgroup differences (random effects): $Chi^2 = 1.48$, df = 2 (P = 0.4763)									

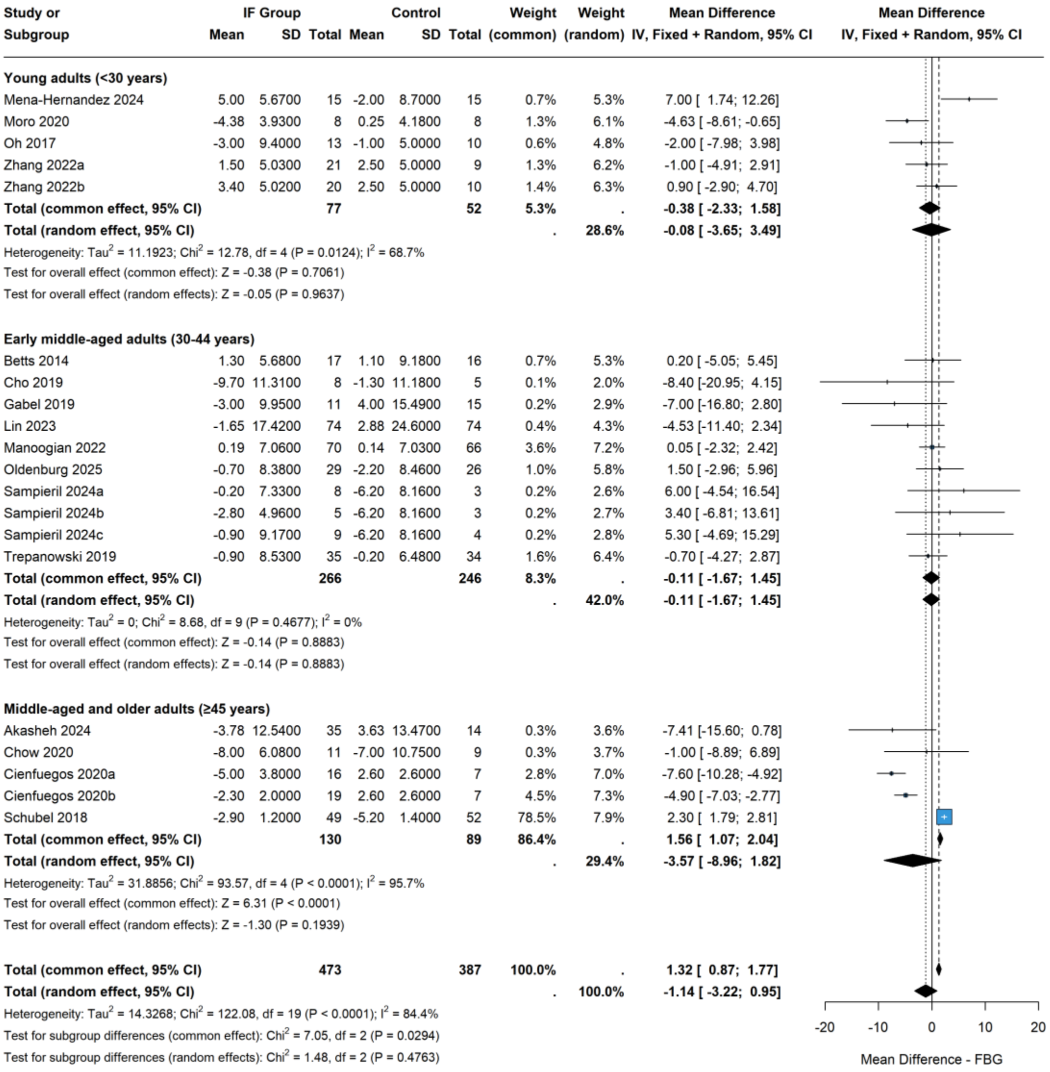

Figure S32. Age-stratified forest plot of the effect of intermittent fasting on homeostatic model assessment of insulin resistance (HOMA-IR).The forest plot illustrates the mean differences (MD) and 95% confidence intervals (CIs) comparing intermittent fasting interventions with control diets across three distinct age cohorts: young (<30 years), early middle-aged (30–44 years), and middle-aged and older (≥45 years) adults. The squares represent the point estimate of each individual trial, with the size of the square proportional to the study's assigned weight in the random-effects meta-analysis. The horizontal lines denote the 95% CIs. The diamonds represent the pooled effect estimates for each age subgroup and the overall analysis. The solid vertical line indicates the line of no effect (MD = 0).

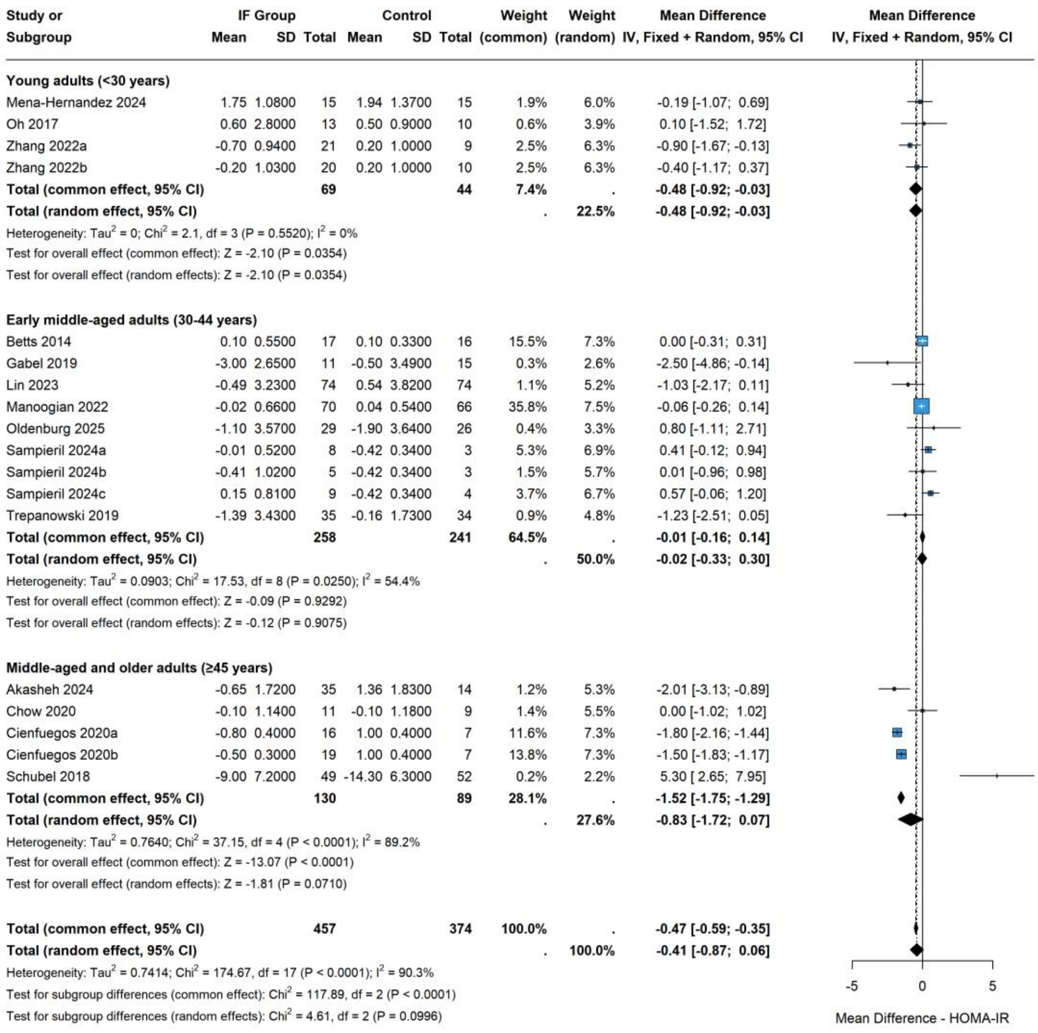

Figure S33. Age-stratified forest plot of the effect of intermittent fasting on systolic blood pressure (SBP).The forest plot illustrates the mean differences (MD) and 95% confidence intervals (CIs) comparing intermittent fasting interventions with control diets across three distinct age cohorts: young (<30 years), early middle-aged (30–44 years), and middle-aged and older (≥45 years) adults. The squares represent the point estimate of each individual trial, with the size of the square proportional to the study's assigned weight in the random-effects meta-analysis. The horizontal lines denote the 95% CIs. The diamonds represent the pooled effect estimates for each age subgroup and the overall analysis. The solid vertical line indicates the line of no effect (MD = 0).

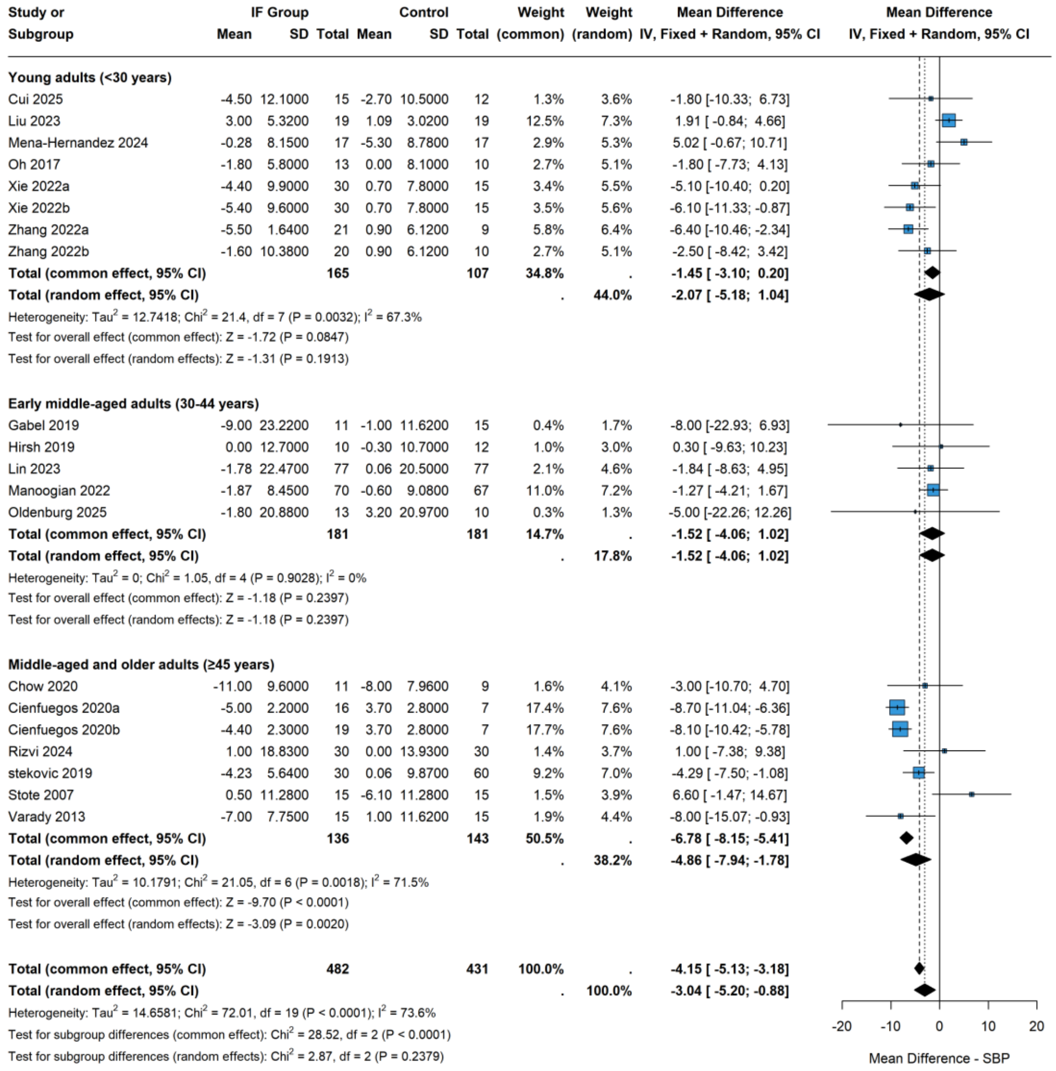

Figure S34. Age-stratified forest plot of the effect of intermittent fasting on diastolic blood pressure (DBP).The forest plot illustrates the mean differences (MD) and 95% confidence intervals (CIs) comparing intermittent fasting interventions with control diets across three distinct age cohorts: young (<30 years), early middle-aged (30–44 years), and middle-aged and older (≥45 years) adults. The squares represent the point estimate of each individual trial, with the size of the square proportional to the study's assigned weight in the random-effects meta-analysis. The horizontal lines denote the 95% CIs. The diamonds represent the pooled effect estimates for each age subgroup and the overall analysis. The solid vertical line indicates the line of no effect (MD = 0).

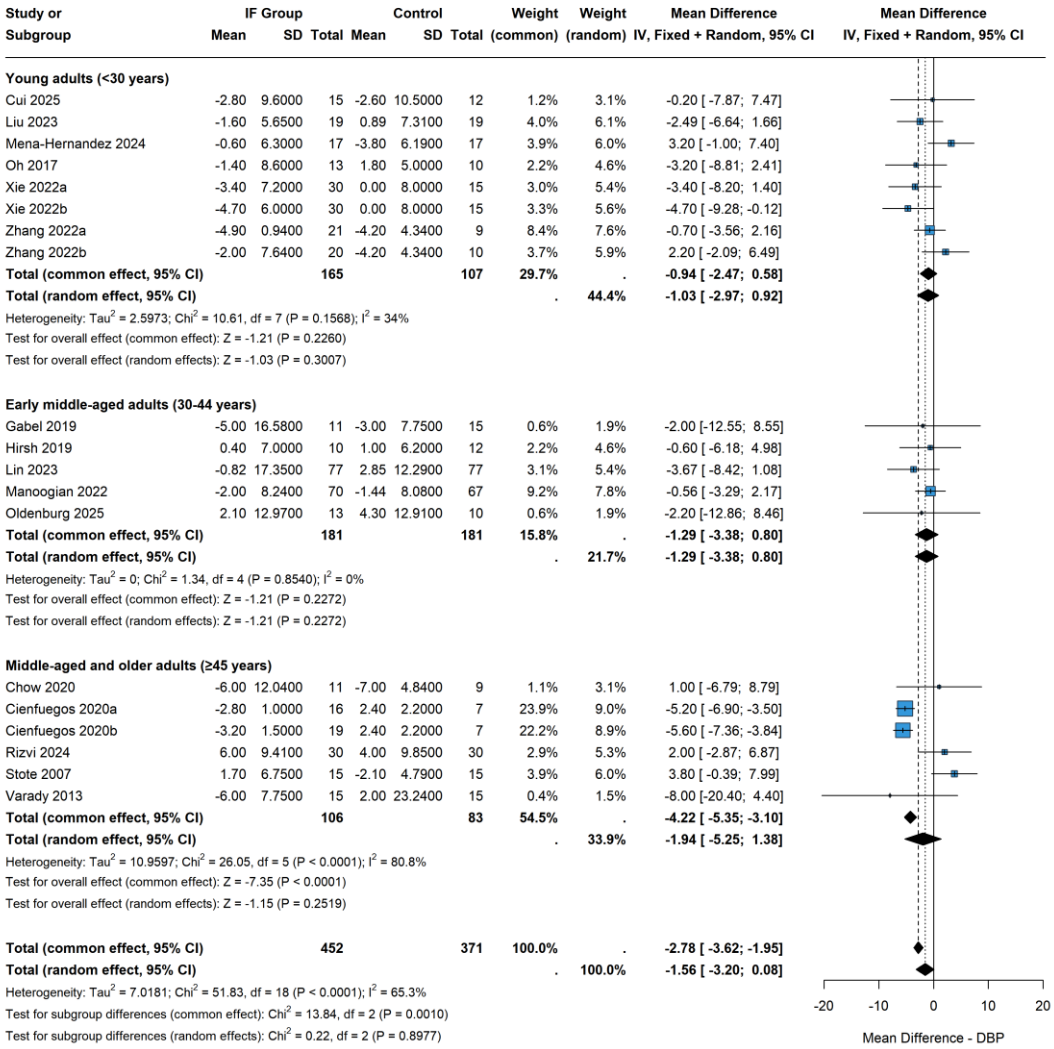

Supplement: Supplementary file 1 [file nutrients-18-01799-s001.zip › nutrients-4319700-supplementary.pdf]
